# Construction of sulfur-containing *N*-vinylimides: *N*-addition of imides to propargyl sulfonium salts†

**DOI:** 10.1039/d2ra01117d

**Published:** 2022-04-26

**Authors:** Shou-Jie Shen, Le-Mei Wang, Guo-Mei Gong, Yan-Jiao Wang, Jin-Yan Liang, Jun-Wen Wang

**Affiliations:** Key Laboratory of Magnetic Molecules, Magnetic Information Materials Ministry of Education, The School of Chemical and Material Science, Shanxi Normal University Linfen 041004 China shoujie_shen@outlook.com; College of Life Science, Shanxi Normal University Linfen 041004 China

## Abstract

An *N*-addition reaction between imides and propargyl sulfonium salts was developed to afford sulfur-containing *N*-vinylimides with moderate to excellent yields. Under the activation of NaOAc·3H_2_O, imides could undergo deprotonation and propargyl sulfonium salts could isomerize to allenic sulfonium salts. The *N*-nucleophilic attack initiates the reaction and gives the desired products. Various imides, including arylimides, aliphatic imides and *N*-(arylsulfonyl) alkyl acylamides, and even bioactive saccharin, thalidomide and pomalidomide could provide organosulfur *N*-vinylimides compounds. The simple, mild and metal-free reaction conditions, the broad scope of substrates, gram-scale synthesis and convenient transformation embody the synthetic superiority of this process.

## Abstract 2

An *N*-addition reaction between imides and propargyl sulfonium salts was developed to afford sulfur-containing *N*-vinylimides with moderate to excellent yields.

## Graphical abstract

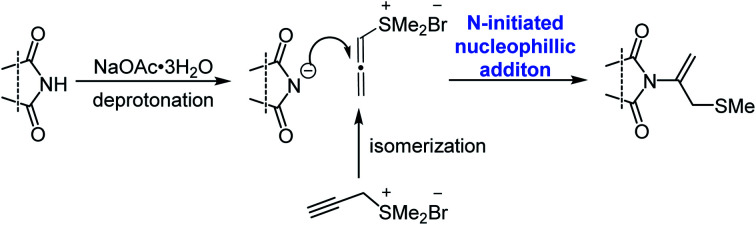

*N*-vinylimides represent crucial structural motifs due to the importance of these frameworks (Fig. 1), which are the core structure found in biologically active structures,^1^ functional materials^2^ and natural products such as the parazoanthines A–E.^3^ They can also serve as versatile synthetic intermediates in the synthesis of β-2-amino acid derivatives^4^ and other complex structures.^5^

**Fig. 1:**
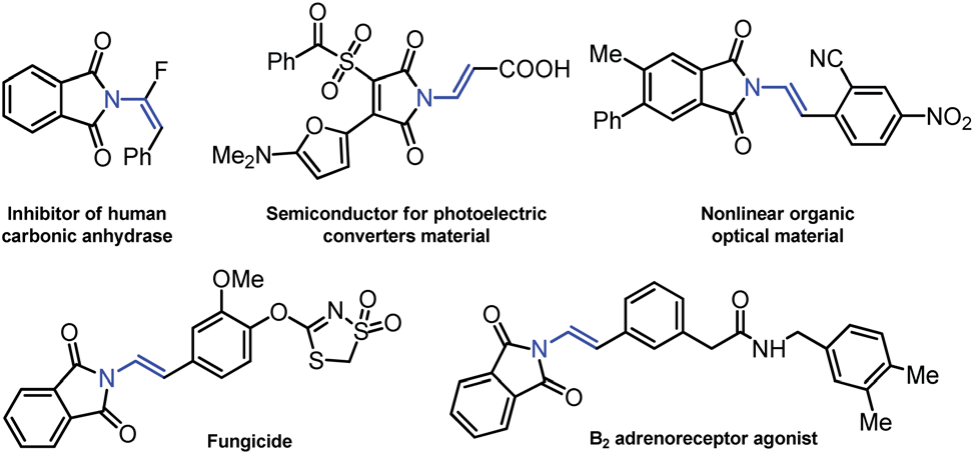
Representative functional *N*-vinylimides scaffolds.

Due to the importance of these *N*-vinylimides frameworks, the growing interest in this featured moiety has catalyzed a recent spurt of attention for methodology appropriate for its construction. Conventionally, protocols for the synthesis of this important substrate class included transition-metal-catalyzed en-imidic C(sp^2^)–N bond formation reactions of imides with vinyl halides, pseudohalides or alkynes. The main strategies involved Cu-catalyzed Chan–Lam–Evans reactions,^6^ Ru-catalyzed hydroimidation reaction of imide with alkyne^7^ and Pd-catalyzed oxidative amination of alkenes.^8^ In 2021, Sandtorv's group reported Cu-catalyzed Chan–Lam–Evans reaction for coupling cyclic imides and alkenylboronic acids by forming C(sp^2^)–N-bonds, enables the practical and mild preparation of (*E*)-enimides (Scheme 1a).^6*a*^ In 2020, Schaub's group reported Ru-phosphine catalyzed hydroimidation reaction of cyclic amides with acetylene under low pressure, affording new method for synthesis of *N*-vinylimides (Scheme 1b).^7*a*^ Hull's group reported Pd-catalyzed anti-Markovnikov oxidative amination reaction, alkenes are shown to react with imides in the presence of a palladate catalyst to generate the terminal imide, providing mild and robust complementary routes (Scheme 1c).^8*a*^ Besides, rarely examples of organo-catalytic conjugate additions of imides to acetylene can also provide methods for the synthesis of *N*-vinylimides.^9^

**Scheme 1:**
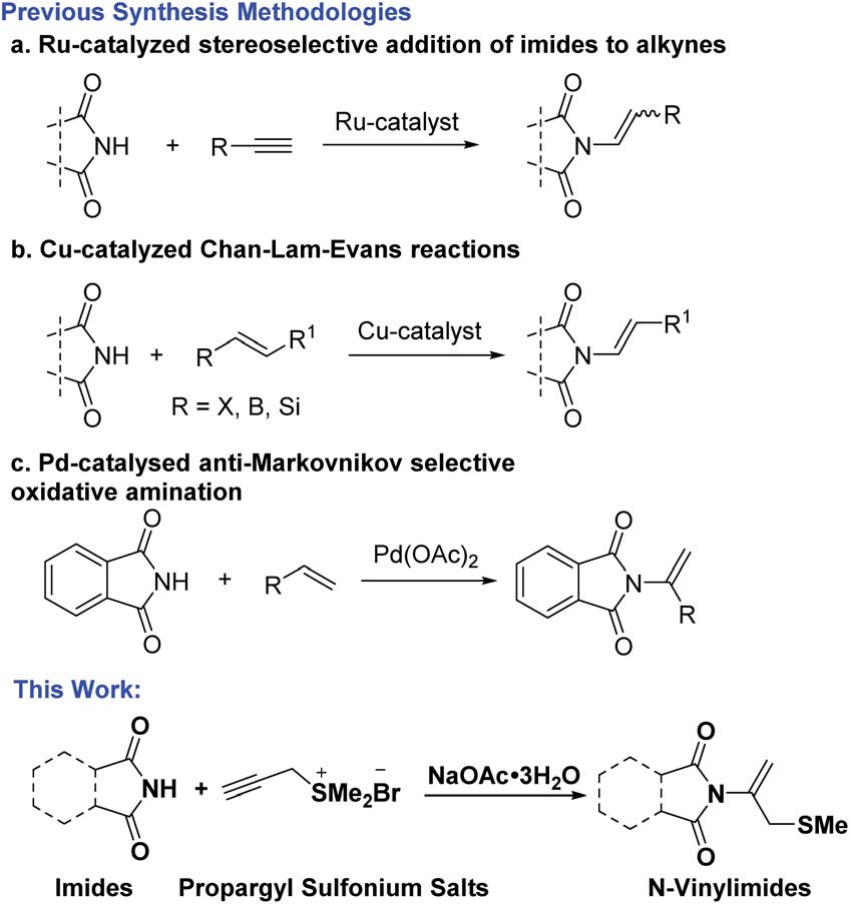
Approaches for vinylation of imides.

The previously reported synthesis strategies mainly involved the use of expensive Ru and Pd-catalysts, otherwise toxic copper catalyst, and the structural limitations imposed to phthalimide and therefore specialized. Considering the limitation in generality, the harsh reaction condition, and the use of metal-catalysis decreased the attractiveness for synthetic applications, the development of new kind of vinylation reagents and their application of building *N*-vinylimides in a simple, mild, metal-free and efficient manner are highly desirable.

Our earlier work inspired our interest in synthesis of *N*-vinylimides by employing propargyl sulfonium salts as vinylation reagent. We have been exploring new reaction patterns of sulfonium salts and developed propargyl sulfonium salts involved [3 + 2] annulation/substitution reaction and *N*-addition/[2,3]-sigmatropic rearrangement reaction in an acyclic model.^10^ Based on our processive interests on constructing *N*-functionalized vinylation reaction and exploring the diverse reactive pathway of propargyl sulfonium salts, we herein report the realization of inorganic base promoted *N*-addition reaction of imides and propargyl sulfonium salts, delivering potential bioactive sulfur-containing *N*-vinylimides in moderate to excellent yields (Scheme 1).

We began our investigation by selecting phthalimide 1a and propargyl sulfonium salt 2a as model substrates (Table 1). When phthalimide 1a (0.3 mmol, 1.0 equiv.), NaOAc·3H_2_O (0.45 mmol, 1.5 equiv.) in CH_3_CN (3.0 mL, *c* = 0.1 M) were mixed, the reaction mixture was stirred for 10 min at 22 °C and propargyl sulfonium salt 2a (0.45 mmol, 1.5 equiv.) was added in one portion. The reaction was stirred for 6 h continuously, phthalimide 1a was consumed completely and afforded *N*-addition product 3a with 71% yield (Table 1). Extensive exploration of a range of bases indicated that NaOAc·3H_2_O was the most efficient to promote the process. Replacement of NaOAc·3H_2_O with anhydrous NaOAc, Na_2_CO_3,_ K_2_CO_3_, Cs_2_CO_3_, KOH and lithium carbonate gave related product with 24–46% yields (Table 1, entries 1–7). Replacement of either stronger inorganic bases such as NaH and KO^*t*^Bu, or organic base Et_3_N and DBU could not promote the reaction efficiently (Table 1, entries 8–11, yields from 33 to 40%). Screening of solvents including THF, CHCl_3_, DCE, DCM, and toluene did not give better yield (Table 1, entries 12–16, yields from trace to 37%). Temperature affected the reaction greatly and the desired product was obtained only in 11% yield when the reaction was conducted at 22 °C (entry 17 in Table 1). As the temperature was risen to 30 and 60 °C, the product 3a was obtained gradually to 23 and 61%, respectively (Table 1, entries 18 and 19). When the temperature was risen to 80 and 90 °C, the reaction efficiency was slightly decreased to 51 and 49%, respectively (entries 20 and 21 in Table 1). We also probed the influence of the ratio of sulfonium salts 2a and NaOAc·3H_2_O (for reaction details, see ESI†).

**Table 1:** Optimization of the reaction conditions^a^

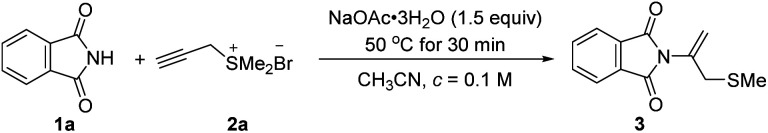				
Entry	Base	Solvent	Temp. (°C)	Yield^b^ (%)
1	NaOAc	CH_3_CN	50	45
2	Na_2_CO_3_	CH_3_CN	50	42
3	K_2_CO_3_	CH_3_CN	50	46
4	Cs_2_CO_3_	CH_3_CN	50	39
5	KOH	CH_3_CN	50	24
6	LiOAc·2H_2_O	CH_3_CN	50	33
7	LiOAc	CH_3_CN	50	42
8	NaH	CH_3_CN	50	40
9	KO^*t*^Bu	CH_3_CN	50	37
10	Et_3_N	CH_3_CN	50	36
11	DBU	CH_3_CN	50	33
12	NaOAc·3H_2_O	THF	50	37
13	NaOAc·3H_2_O	CHCl_3_	50	34
14	NaOAc·3H_2_O	DCE	50	28
15	NaOAc·3H_2_O	DCM	50	35
16	NaOAc·3H_2_O	Toluene	50	Trace
17^c^	NaOAc·3H_2_O	CH_3_CN	22	11
18^c^	NaOAc·3H_2_O	CH_3_CN	30	23
19^c^	NaOAc·3H_2_O	CH_3_CN	60	61
20^c^	NaOAc·3H_2_O	CH_3_CN	80	51
21^c^	NaOAc·3H_2_O	CH_3_CN	90	49

a Unless otherwise noted, the reactions were performed under air and imide 1a (0.3 mmol, 1.0 equiv.), base (0.45 mmol, 1.5 equiv.) in solvent (3.0 mL, *c* = 0.1 M) were mixed, the reaction mixture was stirred for 10 min at 22 °C. Then propargyl sulfonium salt 2a (0.45 mmol, 1.5 equiv.) was added in one portion. The reaction was stirred at 50 °C for 6 h until starting material 1a was fully consumed (monitored by TLC).

b Isolated yield. DCE: 1,2-dichloroethane; DCM: dichloromethane.

c With the ratio of 1a : 2a : NaOAc·3H_2_O = 1 : 1.5 : 1.5.

Having established the optimized conditions, we commenced to explore the substrate scope of the reaction. A selection of arylimides and aliphatic imides was next investigated with propargyl sulfonium salt 2a in Scheme 2. Generally, arylimides containing electron-withdrawing group such as tetrachloro-, 4-bromo-, 4-nitro- and 3-nitrophthalimide provided desired *N*-vinylimides products 3b–3e with moderate yields (Scheme 2, 3b–3e, with yields of 27–52%), probably due to the electron withdrawing effect of substituents. 1,8-Naphthalimide and 2,3-naphthalimide were well-tolerated to provide *N*-vinylimide products 3f and 3g with 62 and 65% yields, respectively. 3,4-Pyridinedicarboximide could also be engaged in the reaction to obtain 3h with yield of 43%. Subsequently, we went on to evaluate the reactivity of aliphatic imides. Unexpected, maleimide could not provide the desired *N*-nucleophilic addition product under the optimized conditions with recovering of the starting material. Oppositely, the method was high yielding and tolerable to succinimide and substituted succinimides. Succinimide and substituted succinimides worked well to deliver *N*-vinylimides products 3j–3o with moderate to excellent yields of 52–93%.^11^ Continuously, we evaluated the reactive effectiveness of glutarimide and substituted glutarimides. Under optimized conditions, glutarimide and substituted glutarimides could also react with 2a and give desired products 3p, 3q and 3r with yields of 32, 24 and 24%, respectively.

**Scheme 2:**
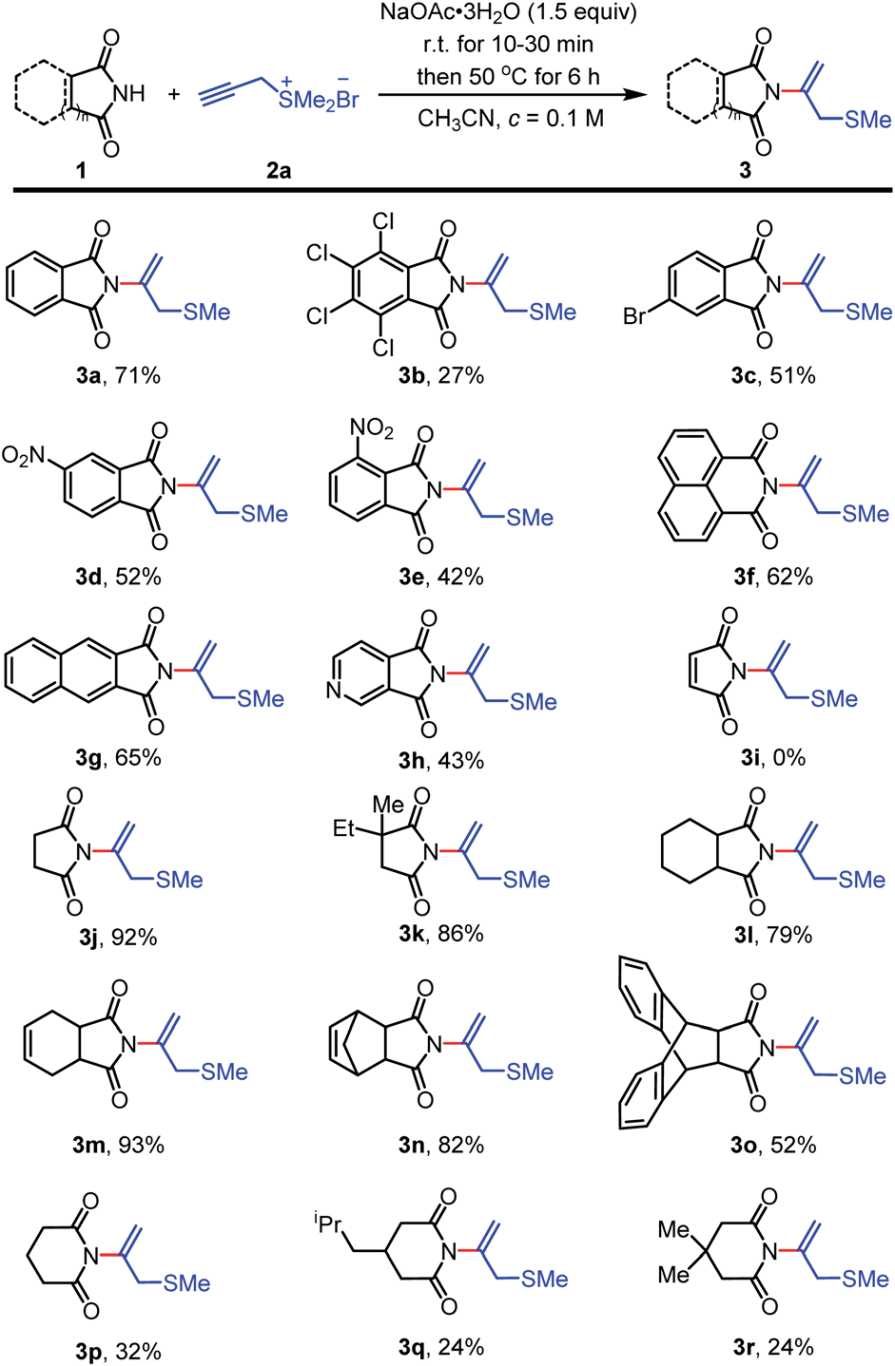
Scope of imides^*a*^. ^*a*^Unless otherwise noted, the reactions were performed under air and imide 1 (0.3 mmol, 1.0 equiv.), NaOAc·3H_2_O (0.45 mmol, 1.5 equiv.) in CH_3_CN (3.0 mL, *c* = 0.1 M) were mixed, the reaction mixture was stirred for 10 min at 22 °C. Then propargyl sulfonium salt 2a (0.45 mmol, 1.5 equiv.) was added in one portion. The reaction was stirred at 50 °C for 6 h until starting material 1 was fully consumed (monitored by TLC). ^*b*^Isolated yield.

Surprising reaction appeared when we explored the reaction of tetrahydro-1*H*-4,7-epoxyisoindole-1,3(2*H*)-dione 1s and 1t (Scheme 3).^12^ Under the optimized conditions, 1s worked well with propargyl sulfonium salt 2a to deliver the corresponding product 3s with yield of 65%. Under the same conditions, compound 1t gave the desired *N*-vinylimide product 3t with yield of 35%, meanwhile with the unexpected *N*-vinylimide product 3i with yield of 16%, which unavailable in the reaction of maleimide with propargyl sulfonium salt 2a, probably due to the retro-Diels–Alder reaction of 1t with generation of maleimide intermediate.

**Scheme 3:**
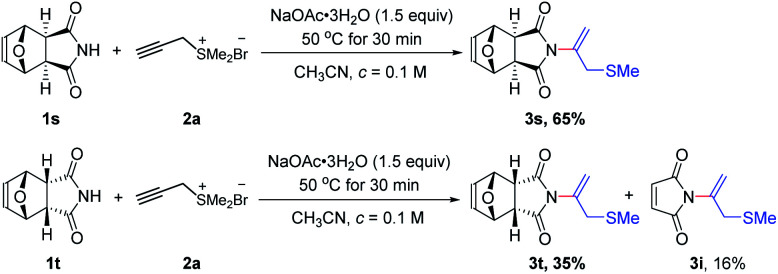
Scope of imides.

The reaction performance could also be adapted to *N*-(arylsulfonyl) alkyl acylamides (Scheme 4). The method smoothly transferred electron-deficient aryl sulfonyl acylamides to form *N*-vinylimide products such as 5a–5e in moderate yields. In contrast, when *N*-(arylsulfonyl) aryl acylamides 5i and *N*-(arylacyl) alkyl acylamides 5j–5l were involved, the reaction was sluggish and no desired *N*-vinylimide products could be obtained probably due to its low nucleophilicity (Scheme 4, 5i–5l).

**Scheme 4:**
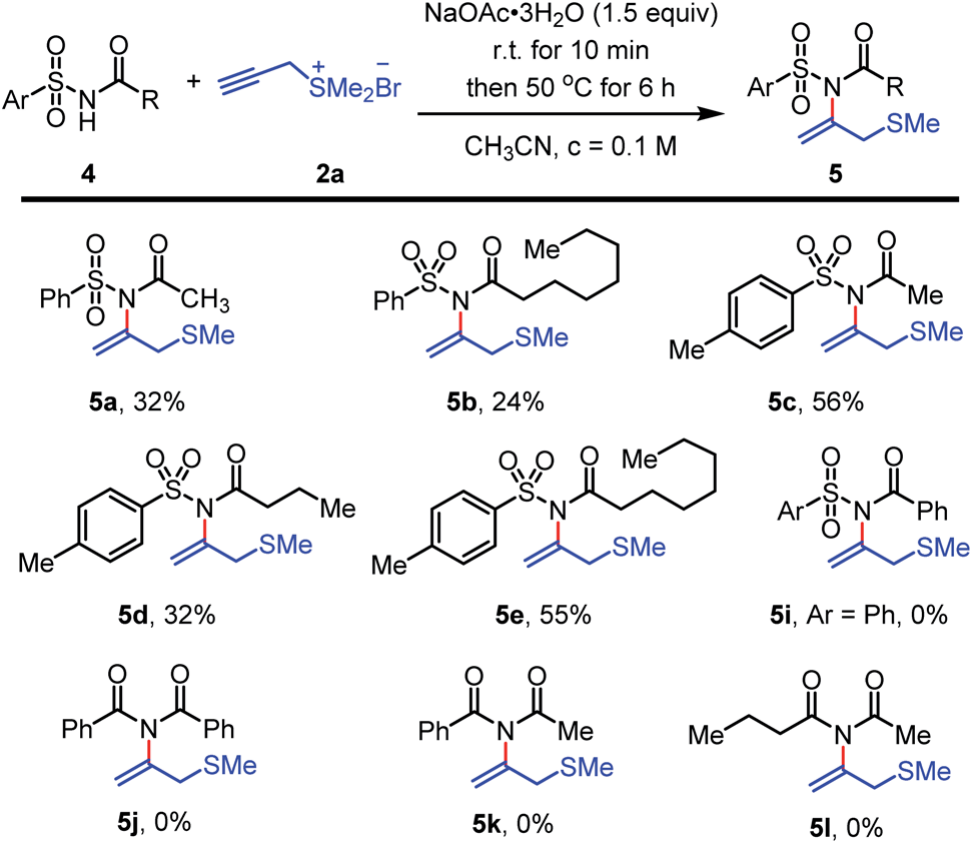
Scope of aryl sulfonyl amides and carbonimides^*a*^, ^*a*^Unless otherwise noted, the reactions were performed under air and imide 4 (0.3 mmol, 1.0 equiv.), NaOAc·3H_2_O (0.45 mmol, 1.5 equiv.) in CH_3_CN (3.0 mL, *c* = 0.1 M) were mixed, the reaction mixture was stirred for 10 min at 22 °C. Then propargyl sulfonium salt 2a (0.45 mmol, 1.5 equiv.) was added in one portion. The reaction was stirred at 50 °C for 6 h until starting material 4 was fully consumed (monitored by TLC). ^*b*^Isolated yield.

To further broaden the scope of the reaction, other representative propargyl sulfonium salts were also investigated (Scheme 5). Trimethylsilyl contained propargyl sulfonium salt 2b could be applied to the reaction and the desilylation product 3a was obtained with a yield of 63%. The method was high yielding and tolerable to diverse bioactive molecules, such as saccharin, thalidomide and pomalidomide. Saccharin derivatives have been reported as good hCAs inhibitors,^13^ and thalidomide and pomalidomide belongs to an important class of molecules known as immunomodulatory imide drugs (IMiDs).^14^ We found that under optimized conditions, saccharin, thalidomide and pomalidomide were also compatible with propargyl sulfonium salt 2a and provided the corresponding products 7, 9 and 11 with 52, 87, and 80% yields, respectively (Scheme 5).

**Scheme 5:**
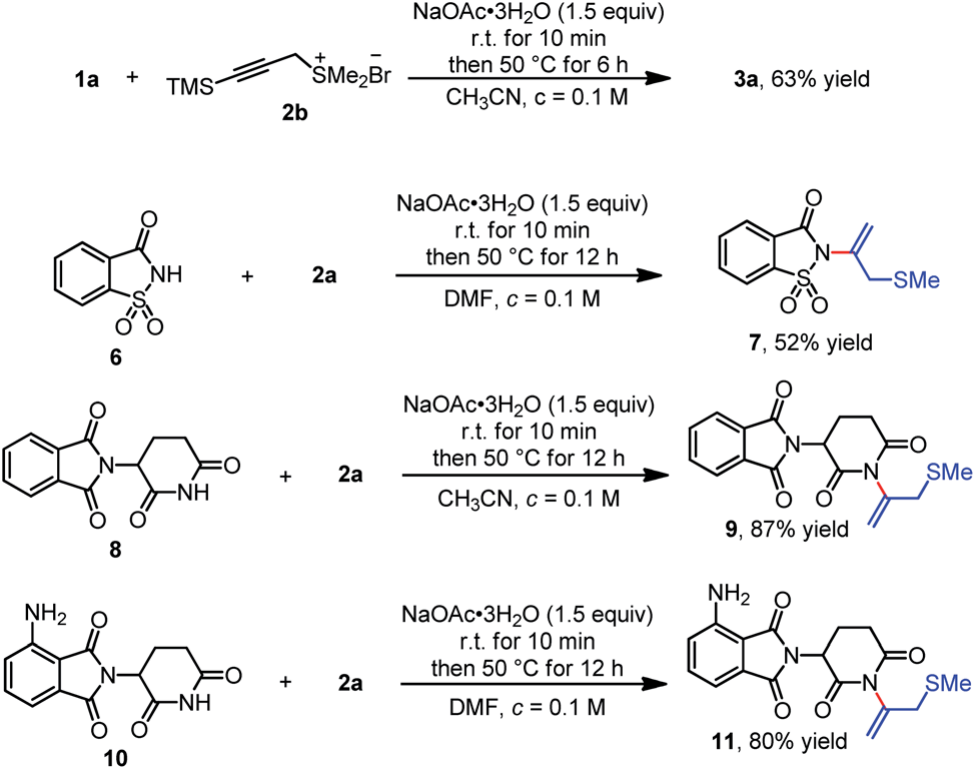
Scope of propargyl sulfonium salts and bioactive molecules.

To demonstrate the synthetic utility of this protocol, we performed the gram-scale operation using phthalimide 1a (1.01 g, 6.8 mmol) and propargyl sulfonium salt 2a (1.5 equiv.) as the representative substrates under the optimized conditions, providing the related product 3a (1.03 g) with 65% yield (Scheme 6). The typical transformation was also conducted by oxidation of compound 3a with *m*-chloro peroxybenzoic acid (3.0 equiv.) and sulfonyl product 12 was obtained with 94% yield.

**Scheme 6:**
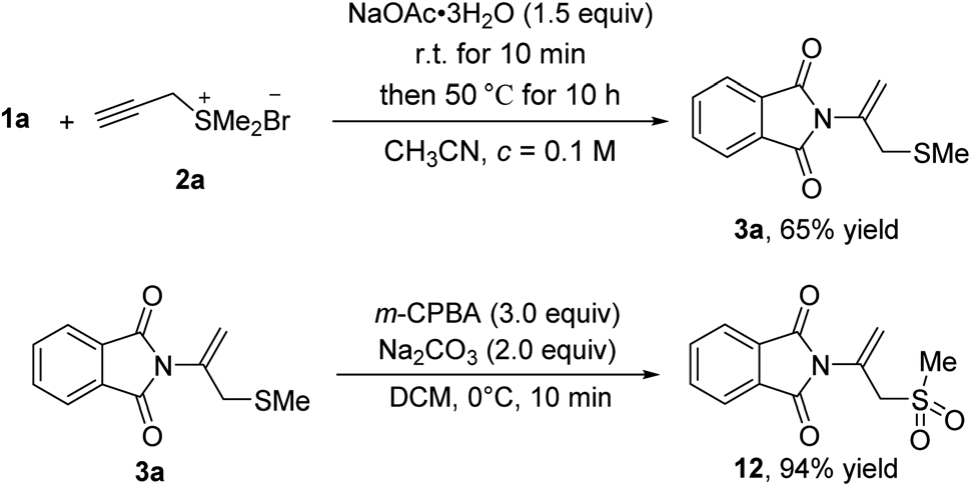
Gram-scale synthesis and further transformation.

According to the previous reports on α-alkylidene pyrazolinones and propargyl sulfonium ylides,^10*b*,*c*^ a possible mechanism is proposed to account for the formation of *N*-vinylimides 3 (Scheme 7). Under the activation of inorganic base NaOAc·3H_2_O, the imides 1 may undergo deprotonation to form intermediate I and propargyl sulfonium salt 2a can isomerize to allenic sulfonium salts II. The *N*-nucleophilic attack of I to allenic sulfonium salts II initiates the reaction and gives intermediate III. Subsequently, protonation of the species III and release of MeBr provided the desired product 3.

**Scheme 7:**
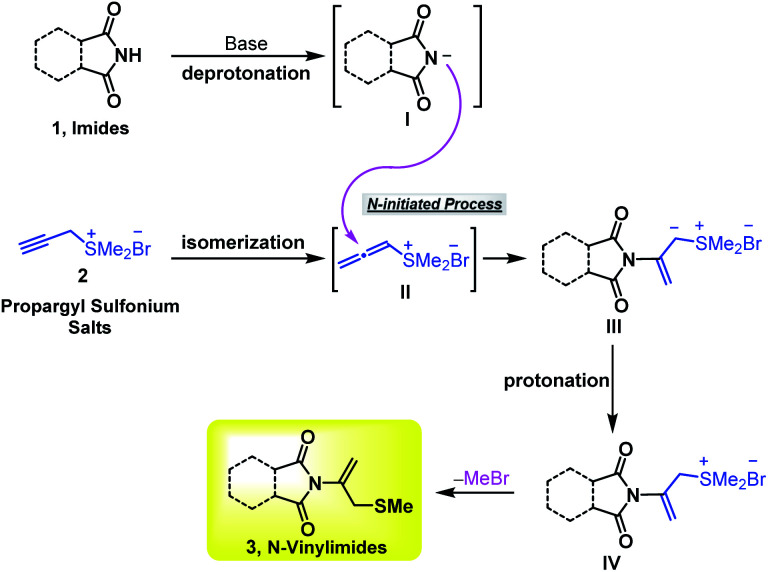
Plausible reaction mechanism.

In summary, we have developed NaOAc·3H_2_O promoted *N*-addition reaction between imides and propargyl sulfonium salts, delivering potentially bioactive *N*-vinylimides in moderate to excellent yields. Various imides, including arylimides, aliphatic imides and *N*-(arylsulfonyl) alkyl acylamides, even bioactive saccharin, thalidomide and pomalidomide could tolerate and function to provide organosulfur *N*-vinylimides compounds. Gram-scale synthesis and convenient transformations are also furnished. The simple, mild, metal-free and efficient reaction condition, the broad scope of substrates, gram-scale synthesis and convenient transformation embody the synthetic superiority of this reaction process.

## Experimental

### General information

All reactions were performed in oven-dried or flame-dried round-bottom flasks and vials. Stainless steel syringes and cannula were used to transfer air- and moisture-sensitive liquids. Flash chromatography was performed using silica gel 60 (230–400 mesh) from Aladdin.

### General procedure for substrates

To a flame-dried sealable 3-dram vial equipped with a stir bar was added imides 1 (0.3 mmol, 1.0 equiv.), NaOAc·3H_2_O (0.45 mmol, 1.5 equiv.), subsequently treated CH_3_CN (3 mL, *c* = 0.1 M) was added to vial *via* syringe, the reaction mixture was stirred for 10–30 min at 22 °C. Then propargyl sulfonium salt 2a (0.45 mmol, 1.5 equiv.) was added in one portion. The reaction was stirred at 50 °C for 6–12 h until imides 1 was fully consumed (monitored by TLC). The organic solvent was removed under reduced pressure and purified through column chromatography (eluent: petroleum ether and EtOAc) to afford the desired product 3.

### Characterization data for the products

#### 2-(3-(Methylthio)prop-1-en-2-yl)isoindoline-1,3-dione (3a)

The reaction was carried out on a 0.3 mmol scale by following the general procedure A. The product 3a was purified through column chromatography (PE/EtOAc from 35 : 1 to 25 : 1) as colorless oil (33 mg, 71% yield). IR *ν*_max_ (neat)/cm^−1^: 3465, 3437, 2925, 2848, 1768, 1711, 1635, 1460, 1375, 1087, 717; ^1^H NMR (600 MHz, CDCl_3_): *δ* 7.82 (dd, *J* = 0, 0.6 Hz, 2H), 7.69 (dd, *J* = 0, 0.6 Hz, 2H), 5.39 (s, 1H), 5.24 (s, 1H), 3.53 (s, 2H), 1.97 (s, 3H); ^13^C NMR (150 MHz, CDCl_3_): *δ* 167.1, 134.3, 134.0, 131.7, 123.7, 116.8, 37.1, 11.5. HRMS (ESI, *m*/*z*): calcd for C_12_H_12_NO_2_S^+^, [M + H]^+^, 234.0583, found 234.0585.

#### 4,5,6,7-Tetrachloro-2-(3-(methylthio)prop-1-en-2-yl)isoindol-ine-1,3-dione (3b)

The reaction was carried out on a 0.3 mmol scale by following the general procedure A. The product 3b was purified through column chromatography (PE/EtOAc from 50 : 1 to 35 : 1) as colorless oil (20 mg, 27% yield). IR *ν*_max_ (neat)/cm^−1^: 3452, 3437, 2974, 2925, 2855, 1719, 1648, 1388, 1368, 1157, 1116, 730; ^1^H NMR (600 MHz, CDCl_3_): *δ* 5.53 (s, 1H), 5.35 (s, 1H), 3.57 (s, 2H), 2.05 (s, 3H); ^13^C NMR (150 MHz, CDCl_3_): *δ* 162.3, 140.5, 133.4, 130.1, 127.2, 117.9, 36.9, 14.6. HRMS (ESI, *m*/*z*): calcd for C_12_H_8_Cl_4_NO_2_S^+^, [M + H]^+^, 369.9024, found 369.9025.

#### 5-Bromo-2-(3-(methylthio)prop-1-en-2-yl)isoindoline-1,3-di-one (3c)

The reaction was carried out on a 0.3 mmol scale by following the general procedure A. The product 3c was purified through column chromatography (PE/EtOAc from 50 : 1 to 25 : 1) as colorless oil (32 mg, 51% yield). IR *ν*_max_ (neat)/cm^−1^: 3465, 3445, 2961, 2910, 2848, 1753, 1712, 1648, 1375, 1087, 892; ^1^H NMR (600 MHz, CDCl_3_): *δ* 8.02 (s, 1H), 7.89 (dd, *J* = 0.6, 7.8 Hz, 1H), 7.75 (d, *J* = 8.4 Hz, 1H), 5.47 (s, 1H), 5.30 (s, 1H), 3.57 (s, 2H), 2.02 (s, 3H); ^13^C NMR (150 MHz, CDCl_3_): *δ* 166.3, 165.7, 137.3, 133.8, 133.3, 130.2, 129.2, 127.0, 125.0, 117.0, 37.0, 14.5. HRMS (ESI, *m*/*z*): calcd for C_12_H_11_BrNO_2_S^+^, [M + H]^+^, 311.9688, found 311.9687.

#### 2-(3-(Methylthio)prop-1-en-2-yl)-5-nitroisoindoline-1,3-dione (3d)

The reaction was carried out on a 0.3 mmol scale by following the general procedure A. The product 3d was purified through column chromatography (PE/EtOAc from 15 : 1 to 5 : 1) as colorless oil (29 mg, 52% yield). IR *ν*_max_ (neat)/cm^−1^: 3465, 3437, 3108, 2939, 2910, 2856, 1782, 1720, 1648, 1537, 1375, 1339, 1087, 926, 722; ^1^H NMR (600 MHz, CDCl_3_): *δ* 8.73 (d, *J* = 1.8 Hz, 1H), 8.66 (dd, *J* = 1.8, 7.8 Hz, 1H), 8.11 (d, *J* = 7.8 Hz, 1H), 5.54 (s, 1H), 5.38 (s, 1H), 3.60 (s, 2H), 2.05 (s, 3H); ^13^C NMR (150 MHz, CDCl_3_): *δ* 165.0, 164.7, 151.9, 136.0, 133.6, 133.1, 129.5, 124.9, 119.1, 117.5, 36.9, 14.5. HRMS (ESI, *m*/*z*): calcd for C_12_H_11_N_2_O_4_S^+^, [M + H]^+^, 279.0434, found 279.0436.

#### 2-(3-(Methylthio)prop-1-en-2-yl)-4-nitroisoindoline-1,3-dione (3e)

The reaction was carried out on a 0.3 mmol scale by following the general procedure A. The product 3e was purified through column chromatography (PE/EtOAc from 15 : 1 to 6 : 1) as colorless oil (23 mg, 42% yield). IR *ν*_max_ (neat)/cm^−1^: 3470, 3430, 3111, 2942, 2915, 2858, 1789, 1727, 1653, 1543, 1381, 1343, 1092, 933, 726; ^1^H NMR (600 MHz, CDCl_3_): *δ* 8.16 (t, *J* = 9.0 Hz, 2H), 7.97 (t, *J* = 7.8 Hz, 1H), 5.54 (s, 1H), 5.37 (s, 1H), 3.58 (s, 2H), 2.05 (s, 3H); ^13^C NMR (150 MHz, CDCl_3_): *δ* 164.6, 161.6, 145.4, 135.6, 133.7, 133.5, 128.8, 127.4, 123.3, 117.8, 36.9, 14.5. HRMS (ESI, *m*/*z*): calcd for C_12_H_11_N_2_O_4_S^+^, [M + H]^+^, 279.0434, found 279.0437.

#### 2-(3-(Methylthio)prop-1-en-2-yl)-1*H*-benzo[de]isoquinoline-1,3(2*H*)-dione (3f)

The reaction was carried out on a 0.3 mmol scale by following the general procedure A. The product 3f was purified through column chromatography (PE/EtOAc from 15 : 1 to 4 : 1) as colorless oil (35 mg, 62% yield). IR *ν*_max_ (neat)/cm^−1^: 3430, 2910, 2897, 1704, 1663, 1593, 1347, 1242, 899, 779; ^1^H NMR (600 MHz, CDCl_3_): *δ* 8.63 (d, *J* = 7.2 Hz, 2H), 8.25 (d, *J* = 8.4 Hz, 2H), 7.78 (t, *J* = 7.8 Hz, 2H), 5.75 (s, 1H), 5.38 (s, 1H), 3.57 (s, 2H), 2.23 (s, 3H); ^13^C NMR (150 MHz, CDCl_3_): *δ* 163.7, 137.6, 134.2, 131.7, 131.5, 128.5, 126.9, 122.7, 118.4, 38.0, 15.6. HRMS (ESI, *m*/*z*): calcd for C_16_H_14_NO_2_S^+^, [M + H]^+^, 284.0740, found 284.0742.

#### 2-(3-(Methylthio)prop-1-en-2-yl)-1*H*-benzo[*f*]isoindole-1,3(2*H*)-dione (3g)

The reaction was carried out on a 0.3 mmol scale by following the general procedure A. The product 3g was purified through column chromatography (PE/EtOAc from 25 : 1 to 1 : 1) as colorless oil (37 mg, 65% yield). IR *ν*_max_ (neat)/cm^−1^: 3458, 3424, 2967, 2925, 2856, 1768, 1712, 1368, 1095, 758; ^1^H NMR (600 MHz, CDCl_3_): *δ* 8.41 (s, 2H), 8.09 (dd, *J* = 3.6, 6.0 Hz, 2H), 7.73 (dd, *J* = 3.6, 6.0 Hz, 2H), 5.54 (s, 1H), 5.40 (s, 1H), 3.66 (s, 2H), 2.09 (s, 3H); ^13^C NMR (150 MHz, CDCl_3_): *δ* 167.0, 135.6, 134.2, 130.3, 129.3, 127.4, 125.2, 117.0, 37.1, 14.6. HRMS (ESI, *m*/*z*): calcd for C_16_H_14_NO_2_S^+^, [M + H]^+^, 284.0740, found 284.0744.

#### 2-(3-(Methylthio)prop-1-en-2-yl)-1*H*-pyrrolo[3,4-*c*]pyridine-1,3(2*H*)-dione (3h)

The reaction was carried out on a 0.3 mmol scale by following the general procedure A. The product 3h was purified through column chromatography (PE/EtOAc from 15 : 1 to 5 : 1) as colorless oil (20 mg, 43% yield). IR *ν*_max_ (neat)/cm^−1^: 3437, 2918, 2848, 1720, 1648, 1607, 1417, 1362, 1095, 892; ^1^H NMR (400 MHz, CDCl_3_): *δ* 9.23 (s, 1H), 9.13 (d, *J* = 3.0 Hz, 1H), 7.82 (d, *J* = 4.8 Hz, 1H), 5.53 (s, 1H), 5.36 (s, 1H), 3.59 (s, 2H), 2.05 (s, 3H); ^13^C NMR (150 MHz, CDCl_3_): *δ* 165.9, 165.6, 155.8, 145.3, 139.0, 133.6, 125.6, 117.6, 117.1, 37.0, 14.5. HRMS (ESI, *m*/*z*): calcd for C_11_H_11_N_2_O_2_S^+^, [M + H]^+^, 235.0536, found 235.0537.

#### 1-(3-(Methylthio)prop-1-en-2-yl)-1*H*-pyrrole-2,5-dione (3i)

The product 3t was purified through column chromatography (PE/EtOAc from 8 : 1 to 1 : 1) as colorless oil (18 mg, 35% yield), 3i was also detected in the reaction colorless oil (6 mg, 16% yield). IR *ν*_max_ (neat)/cm^−1^: 3475, 3434, 2928, 2848, 1707, 1638, 1624, 1391, 1378, 1198; ^1^H NMR (600 MHz, CDCl_3_): *δ* 6.77 (s, 2H), 5.37 (s, 1H), 5.22 (s, 1H), 3.50 (s, 2H), 2.01 (s, 3H); ^13^C NMR (150 MHz, CDCl_3_): *δ* 169.3, 134.1, 133.4, 116.1, 36.9, 14.4. HRMS (ESI, *m*/*z*): calcd for C_8_H_10_NO_2_S^+^, [M + H]^+^, 184.0427, found 184.0429.

#### 1-(3-(Methylthio)prop-1-en-2-yl)pyrrolidine-2,5-dione (3j)

The reaction was carried out on a 0.3 mmol scale by following the general procedure A. The product 3j was purified through column chromatography (PE/EtOAc from 8 : 1 to 4 : 1) as colorless oil (34 mg, 92% yield). IR *ν*_max_ (neat)/cm^−1^: 3473, 3430, 2925, 1704, 1635, 1622, 1388, 1375, 1193; ^1^H NMR (600 MHz, CDCl_3_): *δ* 5.45 (s, 1H), 5.22 (s, 1H), 3.47 (s, 2H), 2.82 (s, 4H), 2.03 (s, 3H); ^13^C NMR (150 MHz, CDCl_3_): *δ* 175.9, 134.4, 117.2, 36.4, 28.3, 14.6. HRMS (ESI, *m*/*z*): calcd for C_8_H_12_NO_2_S^+^, [M + H]^+^, 186.0583, found 186.0584.

#### 3-Ethyl-3-methyl-1-(3-(methylthio)prop-1-en-2-yl)pyrrolidine-2,5-dione (3k)

The reaction was carried out on a 0.3 mmol scale by following the general procedure A. The product 3k was purified through column chromatography (PE/EtOAc from 15 : 1 to 2 : 1) as colorless oil (39 mg, 86% yield). IR *ν*_max_ (neat)/cm^−1^: 3478, 3435, 2929, 1710, 1637, 1652, 1381, 1370, 1198, 630; ^1^H NMR (600 MHz, CDCl_3_): *δ* 5.42 (s, 1H), 5.19 (s, 1H), 3.46 (s, 2H), 2.61 (ABQ, *J* = 18.6 Hz, 2H), 2.01 (s, 3H), 1.83–1.77 (m, 1H), 1.68–1.62 (m, 1H), 1.36 (s, 3H), 0.95 (t, *J* = 7.8 Hz, 3H); ^13^C NMR (150 MHz, CDCl_3_): *δ* 181.7, 174.9, 134.3, 117.0, 44.2, 40.5, 36.4, 31.0, 24.1, 14.5, 8.7. HRMS (ESI, *m*/*z*): calcd for C_11_H_18_NO_2_S^+^, [M + H]^+^, 228.1053, found 228.1055.

#### 2-(3-(Methylthio)prop-1-en-2-yl)hexahydro-1*H*-isoindole-1,3 (2*H*)-dione (3l)

The reaction was carried out on a 0.3 mmol scale by following the General Procedure A. The product 3l was purified through column chromatography (PE/EtOAc from 15 : 1 to 5 : 1) as colorless oil (38 mg, 79% yield). IR *ν*_max_ (neat)/cm^−1^: 3457, 3437, 2972, 2919, 1714, 1645, 1375, 1193, 684, 605; ^1^H NMR (600 MHz, CDCl_3_): *δ* 5.40 (s, 1H), 5.19 (s, 1H), 3.49 (s, 2H), 2.95 (s, 2H), 2.03 (s, 3H), 1.90 (s, 4H), 1.49 (d, *J* = 2.4 Hz, 4H); ^13^C NMR (150 MHz, CDCl_3_): *δ* 178.5, 134.3, 116.7, 40.1, 36.5, 23.9, 21.9, 14.5. HRMS (ESI, *m*/*z*): calcd for C_12_H_18_NO_2_S^+^, [M + H]^+^, 240.1053, found 240.1054.

#### 2-(3-(Methylthio)prop-1-en-2-yl)-3a,4,7,7a-tetrahydro-1*H*-iso-indole-1,3(2*H*)-dione (3m)

The reaction was carried out on a 0.3 mmol scale by following the general procedure A. The product 3m was purified through column chromatography (PE/EtOAc from 10 : 1 to 5 : 1) as colorless oil (44 mg, 93% yield). IR *ν*_max_ (neat)/cm^−1^: 3452, 3437, 2961, 2925, 2848, 1712, 1648, 1375, 1208, 694; ^1^H NMR (600 MHz, CDCl_3_): *δ* 5.93 (s, 2H), 5.39 (s, 1H), 5.15 (s, 1H), 3.41 (s, 2H), 3.15 (t, *J* = 3.0 Hz, 2H), 2.61 (d, *J* = 15.6 Hz, 2H), 2.29 (d, *J* = 13.8 Hz, 2H), 1.98 (s, 3H); ^13^C NMR (600 MHz, CDCl_3_): *δ* 179.0, 134.4, 117.0, 39.0, 36.3, 23.5, 14.5. HRMS (ESI, *m*/*z*): calcd for C_12_H_16_NO_2_S^+^, [M + H]^+^, 238.0896, found 238.0898.

#### 2-(3-(Methylthio)prop-1-en-2-yl)-3a,4,7,7a-tetrahydro-1*H*-4,7-methanoisoindole-1,3(2*H*)-dione (3n)

The reaction was carried out on a 0.3 mmol scale by following the general procedure A. The product 3n was purified through column chromatography (PE/EtOAc from 15 : 1 to 8 : 1) as colorless oil (41 mg, 82% yield). IR *ν*_max_ (neat)/cm^−1^: 3458, 3437, 2982, 2918, 1704, 1648, 1375, 1355, 1193, 604; ^1^H NMR (600 MHz, CDCl_3_): *δ* 6.20 (s, 2H), 5.35 (s, 1H), 5.05 (s, 1H), 3.45 (s, 2H), 3.35 (t, *J* = 1.2 Hz, 2H), 3.32 (s, 2H), 2.01 (s, 3H); ^13^C NMR (150 MHz, CDCl_3_): *δ* 176.6, 134.7, 134.3, 117.1, 52.1, 45.8, 45.2, 36.4, 14.5. HRMS (ESI, *m*/*z*): calcd for C_13_H_16_NO_2_S^+^, [M + H]^+^, 250.0896, found 250.0898.

#### 13-(3-(Methylthio)prop-1-en-2-yl)-11,15-dihydro-9*H*-9,10-[3,4] epipyrroloanthracene-12,14(10*H*,13*H*)-dione (3o)

The reaction was carried out on a 0.3 mmol scale by following the general procedure A. The product 3o was purified through column chromatography (PE/EtOAc from 13 : 1 to 4 : 1) as colorless oil (38 mg, 52% yield). IR *ν*_max_ (neat)/cm^−1^: 3458, 3429, 2961, 2925, 1768, 1712, 1648, 1473, 1375, 1208, 766; ^1^H NMR (600 MHz, CDCl_3_): *δ* 7.42 (dd, *J* = 3.6, 5.4 Hz, 2H), 7.32 (dd, *J* = 3.0, 5.4 Hz, 2H), 7.21 (dd, *J* = 3.0, 5.4 Hz, 2H), 7.18 (dd, *J* = 3.0, 5.4 Hz, 2H), 5.18 (s, 1H), 4.83 (s, 2H), 4.27 (s, 1H), 3.31 (s, 2H), 3.03 (s, 2H), 1.94 (s, 3H); ^13^C NMR (150 MHz, CDCl_3_): *δ* 175.7, 141.3, 138.7, 134.2, 127.0, 126.7, 125.0, 124.2, 116.9, 46.9, 45.7, 36.0, 14.6. HRMS (ESI, *m*/*z*): calcd for C_22_H_20_NO_2_S^+^, [M + H]^+^, 362.1209, found 362.1212.

#### 1-(3-(Methylthio)prop-1-en-2-yl)piperidine-2,6-dione (3p)

: The reaction was carried out on a 0.3 mmol scale by following the general procedure A. The product 3p was purified through column chromatography (PE/EtOAc from 8 : 1 to 5 : 1) as colorless oil (13 mg, 32% yield). IR *ν*_max_ (neat)/cm^−1^: 3465, 3360, 2988, 2925, 2904, 1671, 1586, 1285, 1213, 1102, 779; ^1^H NMR (600 MHz, CDCl_3_): *δ* 5.53 (s, 1H), 5.11 (s, 1H), 3.32 (s, 2H), 2.73 (t, *J* = 6.6 Hz, 4H), 2.09 (s, 3H), 2.05–2.00 (m, 2H); ^13^C NMR (150 MHz, CDCl_3_): *δ* 171.9, 137.0, 118.1, 37.8, 32.9, 17.2, 15.4. HRMS (ESI, *m*/*z*): calcd for C_9_H_14_NO_2_S^+^, [M + H]^+^, 200.0740, found 200.0743.

#### 4-Isobutyl-1-(3-(methylthio)prop-1-en-2-yl)piperidine-2,6-di-one (3q)

The reaction was carried out on a 0.3 mmol scale by following the general procedure A. The product 3q was purified through column chromatography (PE/EtOAc from 20 : 1 to 4 : 1) as colorless oil (12 mg, 24% yield). IR *ν*_max_ (neat)/cm^−1^: 3465, 3430, 2975, 2920, 2894, 1736, 1673, 1486, 1281, 1233, 1132, 734; ^1^H NMR (600 MHz, CDCl_3_): *δ* 5.53 (s, 1H), 5.11 (s, 1H), 3.36 (s, 2H), 2.84 (dd, *J* = 4.2, 16.8 Hz, 2H), 2.39 (dd, *J* = 10.2, 16.8 Hz, 2H), 2.27–2.23 (m, 1H), 2.09 (s, 3H), 1.72–1.68 (m, 1H), 1.28 (dd, *J* = 7.8, 15.0 Hz, 2H), 0.94 (s, 3H), 0.93 (s, 3H); ^13^C NMR (150 MHz, CDCl_3_): *δ* 171.7, 137.0, 118.2, 44.0, 39.2, 37.9, 27.1, 24.8, 22.5, 15.4. HRMS (ESI, *m*/*z*): calcd for C_13_H_22_NO_2_S^+^, [M + H]^+^, 256.1366, found 256.1369.

#### 4,4-Dimethyl-1-(3-(methylthio)prop-1-en-2-yl)piperidine-2,6-dione (3r)

The reaction was carried out on a 0.3 mmol scale by following the general procedure A. The product 3r was purified through column chromatography (PE/EtOAc from 10 : 1 to 5 : 1) as colorless oil (11 mg, 24% yield). IR *ν*_max_ (neat)/cm^−1^: 3465, 3437, 2961, 2918, 2856, 1725, 1676, 1355, 1270, 1242, 1131, 625; ^1^H NMR (600 MHz, CDCl_3_): *δ* 5.52 (s, 1H), 5.10 (s, 1H), 3.36 (s, 2H), 2.59 (s, 4H), 2.10 (s, 3H), 1.18 (s, 6H); ^13^C NMR (150 MHz, CDCl_3_): *δ* 171.4, 136.9, 118.2, 46.5, 37.9, 29.3, 27.8, 15.3. HRMS (ESI, *m*/*z*): calcd for C_11_H_18_NO_2_S^+^, [M + H]^+^, 228.1053, found 228.1056.

#### (3a*R*,4*S*,7*R*,7a*S*)-2-(3-(Methylthio)prop-1-en-2-yl)-3a,4,7,7a-tetrahydro-1*H*-4,7-epoxyisoindole-1,3(2H)-dione (3s)

The reaction was carried out on a 0.3 mmol scale by following the general procedure A. The product 3s was purified through column chromatography (PE/EtOAc from 8 : 1 to 2 : 1) as colorless oil (33 mg, 65% yield). IR *ν*_max_ (neat)/cm^−1^: 3465, 3424, 2918, 2910, 1776, 1712, 1648, 1375, 1193, 871; ^1^H NMR (600 MHz, CDCl_3_): *δ* 6.55 (s, 2H), 5.44 (s, 1H), 5.34 (s, 1H), 5.21 (s, 1H), 3.42 (s, 2H), 2.94 (s, 2H), 2.03 (s, 3H); ^13^C NMR (150 MHz, CDCl_3_): *δ* 175.0, 136.6, 134.2, 117.3, 47.4, 36.4, 14.7. HRMS (ESI, *m*/*z*): calcd for C_12_H_14_NO_3_S^+^, [M + H]^+^, 252.0689, found 252.0692.

#### (3a*R*,4*R*,7*S*,7a*S*)-2-(3-(Methylthio)prop-1-en-2-yl)-3a,4,7,7a-tetrahydro-1*H*-4,7-epoxyisoindole-1,3(2*H*)-dione (3t)

The reaction was carried out on a 0.3 mmol scale by following the general procedure A. The product 3t was purified through column chromatography (PE/EtOAc from 8 : 1 to 1 : 1) as colorless oil (18 mg, 35% yield), 3i was also detected in the reaction colorless oil (6 mg, 16% yield). IR *ν*_max_ (neat)/cm^−1^: 3456, 2443, 3031, 2984, 2908, 1762, 1701, 1654, 1372, 1153, 1132, 905, 865, 706, 618; ^1^H NMR (600 MHz, CDCl_3_): *δ* 6.51 (s, 2H), 5.39–5.38 (m, 2H), 5.36 (s, 1H), 5.06 (s, 1H), 3.61 (dd, *J* = 1.8, 3.6 Hz, 2H), 3.33 (s, 2H), 2.01 (s, 3H); ^13^C NMR (150 MHz, CDCl_3_): *δ* 173.7, 134.8, 134.0, 117.3, 79.7, 46.0, 36.3, 14.5. HRMS (ESI, *m*/*z*): calcd for C_12_H_14_NO_3_S^+^, [M + H]^+^, 252.0689, found 252.0687.

General procedure B: to a flame-dried sealable 3-dram vial equipped with a stir bar was added imides 4 (0.3 mmol, 1.0 equiv.), NaOAc·3H_2_O (0.45 mmol, 1.5 equiv.), subsequently treated CH_3_CN (3 mL, *c* = 0.1 M) was added to vial *via* syringe, the reaction mixture was stirred for 10 min at 22 °C. Then propargyl sulfonium salt 2a (0.45 mmol, 1.5 equiv.) was added in one portion. The reaction was stirred at 50 °C for 6–12 h until imides 4 was fully consumed (monitored by TLC). The organic solvent was removed under reduced pressure and purified through column chromatography (eluent: petroleum ether and EtOAc) to afford the desired product 5.

#### *N*-(3-(Methylthio)prop-1-en-2-yl)-*N*-(phenylsulfonyl)acet-amide (5a)

The reaction was carried out on a 0.3 mmol scale by following the general procedure B. The product 5a was purified through column chromatography (PE/EtOAc from 10 : 1 to 5 : 1) as colorless oil (18 mg, 32% yield). IR *ν*_max_ (neat)/cm^−1^: 3478, 2430, 2925, 2918, 1704, 1635, 1362, 1339, 1249, 1172, 1080, 722, 623; ^1^H NMR (600 MHz, CDCl_3_): *δ* 8.06 (d, *J* = 7.8 Hz, 2H), 7.66 (t, *J* = 7.2 Hz, 1H), 7.56 (t, *J* = 7.8 Hz, 1H), 5.72 (s, 1H), 5.26 (s, 1H), 3.54 (s, 2H), 2.23 (s, 3H), 2.20 (s, 3H); ^13^C NMR (150 MHz, CDCl_3_): *δ* 169.9, 141.1, 138.7, 133.9, 129.0, 128.7, 120.6, 39.8, 23.8, 15.9. HRMS (ESI, *m*/*z*): calcd for C_12_H_16_NO_3_S_2_^+^, [M + H]^+^, 286.0566, found 286.0569.

#### *N*-(3-(methylthio)prop-1-en-2-yl)-*N*-(phenylsulfonyl)octan-amide (5b)

The reaction was carried out on a 0.3 mmol scale by following the general procedure B. The product 5b was purified through column chromatography (PE/EtOAc from 35 : 1 to 18 : 1) as colorless oil (18 mg, 24% yield). IR *ν*_max_ (neat)/cm^−1^: 3474, 2454, 2925, 2895, 1705, 1635, 1352, 1339, 1270, 1172, 1080, 823, 722, 623; ^1^H NMR (600 MHz, CDCl_3_): *δ* 8.07 (d, *J* = 8.4 Hz, 2H), 7.65 (t, *J* = 7.2 Hz, 1H), 7.56 (t, *J* = 7.8 Hz, 2H), 5.74 (s, 1H), 5.26 (s, 1H), 3.54 (s, 2H), 2.46 (t, *J* = 7.8 Hz, 2H), 2.01 (s, 3H), 1.56 (dd, *J* = 7.2, 13.8 Hz, 2H), 1.27–1.23 (m, 8H), 0.86 (t, *J* = 6.6 Hz, 3H); ^13^C NMR (150 MHz, CDCl_3_): *δ* 172.7, 140.6, 139.0, 133.8, 129.0, 128.7, 120.6, 39.9, 35.4, 31.5, 28.9, 28.8, 24.5, 22.5, 16.0, 14.0. HRMS (ESI, *m*/*z*): calcd for C_18_H_28_NO_3_S_2_^+^, [M + H]^+^, 370.1505, found 370.1506.

#### *N*-(3-(Methylthio)prop-1-en-2-yl)-*N*-(phenylsulfonyl)acet-amide (5c)

The reaction was carried out on a 0.3 mmol scale by following the general procedure B. The product 5c was purified through column chromatography (PE/EtOAc from 10 : 1 to 4 : 1) as colorless oil (34 mg, 56% yield). IR *ν*_max_ (neat)/cm^−1^: 3445, 3416, 2954, 2925, 2869, 1691, 1642, 1339, 1265, 1157, 1136, 1087, 941, 799, 681, 617; ^1^H NMR (600 MHz, CDCl_3_): *δ* 7.95 (d, *J* = 8.4 Hz, 2H), 7.35 (d, *J* = 8.4 Hz, 2H), 5.71 (s, 1H), 5.25 (s, 1H), 3.53 (s, 2H), 2.46 (s, 3H), 2.22 (s, 3H), 2.20 (s, 3H); ^13^C NMR (150 MHz, CDCl_3_): *δ* 169.9, 145.1, 141.2, 135.8, 130.0, 129.3, 129.1, 127.3, 120.4, 39.8, 23.9, 21.7, 15.9. HRMS (ESI, *m*/*z*): calcd for C_13_H_18_NO_3_S_2_^+^, [M + H]^+^, 300.0723, found 300.0726.

#### *N*-(3-(Methylthio)prop-1-en-2-yl)-*N*-(phenylsulfonyl)butyr-amide (5d)

The reaction was carried out on a 0.3 mmol scale by following the general procedure B. The product 5d was purified through column chromatography (PE/EtOAc from 15 : 1 to 4 : 1) as colorless oil (21 mg, 32% yield). IR *ν*_max_ (neat)/cm^−1^: 3445, 2954, 2925, 2869, 1699, 1642, 1339, 1270, 1172, 1157, 1087, 807, 681, 617; ^1^H NMR (600 MHz, CDCl_3_): *δ* 7.95 (d, *J* = 8.4 Hz, 2H), 7.34 (d, *J* = 7.8 Hz, 2H), 5.73 (s, 1H), 5.25 (s, 1H), 3.53 (s, 2H), 2.45 (t, *J* = 6.6 Hz, 2H), 2.21 (s, 3H), 2.07 (s, 3H), 1.61 (dd, *J* = 7.2, 15.0 Hz, 2H), 0.87 (t, *J* = 7.8 Hz, 3H); ^13^C NMR (150 MHz, CDCl_3_): *δ* 172.5, 145.0, 140.7, 136.0, 129.3, 129.1, 120.5, 39.9, 37.2, 21.7, 18.0, 16.0, 13.5. HRMS (ESI, *m*/*z*): calcd for C_15_H_22_NO_3_S_2_^+^, [M + H]^+^, 328.1036, found 328.1038.

#### *N*-(3-(Methylthio)prop-1-en-2-yl)-*N*-tosyloctanamide (5e)

The reaction was carried out on a 0.3 mmol scale by following the general procedure B. The product 5e was purified through column chromatography (PE/EtOAc from 15 : 1 to 4 : 1) as colorless oil (42 mg, 55% yield). IR *ν*_max_ (neat)/cm^−1^: 3474, 2454, 2923, 2895, 1705, 1635, 1352, 1339, 1270, 1172, 1080, 813, 728, 625; ^1^H NMR (600 MHz, CDCl_3_): *δ* 7.94 (d, *J* = 7.8 Hz, 2H), 7.33 (d, *J* = 7.8 Hz, 2H), 5.72 (s, 1H), 5.25 (s, 1H), 3.52 (s, 2H), 2.45 (t, *J* = 7.2 Hz, 5H), 2.20 (s, 3H), 1.55 (t, *J* = 7.2 Hz, 2H), 1.28–1.22 (m, 8H), 0.86 (t, *J* = 6.6 Hz, 3H); ^13^C NMR (150 MHz, CDCl_3_): *δ* 172.7, 144.9, 140.6, 136.0, 129.3, 129.0, 120.5, 39.9, 35.3, 31.5, 28.9, 28.8, 24.5, 22.5, 21.6, 15.9, 14.0. HRMS (ESI, *m*/*z*): calcd for C_19_H_30_NO_3_S_2_^+^, [M + H]^+^, 384.1662, found 384.1665.

To a flame-dried sealable 3-dram vial equipped with a stir bar was added imides 1a (0.3 mmol, 1.0 equiv.), NaOAc·3H_2_O (0.45 mmol, 1.5 equiv.), subsequently treated CH_3_CN (3 mL, *c* = 0.1 M) was added to vial *via* syringe, the reaction mixture was stirred for 10 min at 22 °C. Then propargyl sulfonium salt 2b (0.45 mmol, 1.5 equiv.) was added in one portion. The reaction was stirred at 50 °C for 6 h until imide 1a was fully consumed (monitored by TLC). The organic solvent was removed under reduced pressure and purified through column chromatography (eluent: petroleum ether and EtOAc) to afford the desired product 3a.

To a flame-dried sealable 3-dram vial equipped with a stir bar was added saccharin 6 (0.3 mmol, 1.0 equiv.), NaOAc·3H_2_O (0.45 mmol, 1.5 equiv.), anhydrous DMF (3 mL, *c* = 0.1 M) was added to vial *via* syringe, the reaction mixture was stirred for 10 min at 22 °C. Then 2a (0.45 mmol, 1.5 equiv.) was added in one portion. The reaction was stirred at 50 °C for 12 h until saccharin 6 was fully consumed (monitored by TLC). The organic solvent was removed under reduced pressure and purified through column chromatography (eluent: petroleum ether and EtOAc) to afford the desired product 7.

#### 2-(3-(Methylthio)prop-1-en-2-yl)benzo[*d*]isothiazol-3(2*H*)-one 1,1-dioxide (7)

The reaction was carried out on a 0.3 mmol scale by following the general procedure. The product 7 was purified through column chromatography (PE/EtOAc from 15 : 1 to 4 : 1) as colorless oil (28 mg, 52% yield). IR *ν*_max_ (neat)/cm^−1^: 3452, 3093, 3072, 3038, 2925, 1733, 1607, 1473, 1319, 1172, 982, 751, 591; ^1^H NMR (600 MHz, CDCl_3_): *δ* 7.91 (dd, *J* = 3.0, 5.4 Hz, 2H), 7.77 (dd, *J* = 2.4, 5.4 Hz, 2H), 5.49 (s, 1H), 5.33 (s, 1H), 3.61 (s, 2H), 2.06 (s, 3H); ^13^C NMR (150 MHz, CDCl_3_): *δ* 158.0, 137.7, 135.0, 134.4, 133.5, 126.8, 125.5, 121.1, 117.2, 36.9, 14.8. HRMS (ESI, *m*/*z*): calcd for C_11_H_12_NO_3_S_2_^+^, [M + H]^+^, 270.0253, found 270.0255.

To a flame-dried sealable 3-dram vial equipped with a stir bar was added thalidomide 8 (0.3 mmol, 1.0 equiv.), NaOAc·3H_2_O (0.45 mmol, 1.5 equiv.), subsequently treated CH_3_CN (3 mL, *c* = 0.1 M) was added to vial *via* syringe, the reaction mixture was stirred for 10 min at 22 °C. Then propargyl sulfonium salt 2a (0.45 mmol, 1.5 equiv.) was added in one portion. The reaction was stirred at 50 °C for 12 h until thalidomide 8 was fully consumed (monitored by TLC). The organic solvent was removed under reduced pressure and purified through column chromatography (eluent: petroleum ether and EtOAc) to afford the desired product 9.

#### 2-(1-(3-(Methylthio)prop-1-en-2-yl)-2,6-dioxopiperidin-3-yl)isoindoline-1,3-dione (9)

The reaction was carried out on a 0.3 mmol scale by following the general procedure. The product 9 was purified through column chromatography (PE/EtOAc from 8 : 1 to 1 : 1) as colorless oil (60 mg, 87% yield). IR *ν*_max_ (neat)/cm^−1^: 3445, 3407, 2961, 2925, 2848, 1720, 1684, 1388, 1262, 1193, 1116, 722; ^1^H NMR (600 MHz, CDCl_3_): *δ* 7.90 (dd, *J* = 3.6, 5.4 Hz, 2H), 7.79–7.78 (m, 2H), 5.57 (s, 1H), 5.23 (s, 1H), 5.99 (dd, *J* = 6.0, 12.6 Hz, 1H), 3.33 (dd, *J* = 15.0, 22.8 Hz, 2H), 3.04 (dd, *J* = 2.4, 13.2 Hz, 1H), 2.90–2.87 (m, 2H), 2.20–2.17 (m, 1H), 2.08 (s, 3H); ^13^C NMR (150 MHz, CDCl_3_): *δ* 170.3, 167.9, 167.3, 136.9, 134.4, 131.8, 123.7, 118.4, 50.1, 37.5, 32.1, 22.1, 15.3. HRMS (ESI, *m*/*z*): calcd for C_17_H_17_N_2_O_4_S^+^, [M + H]^+^, 345.0904, found 345.0905.

To a flame-dried sealable 3-dram vial equipped with a stir bar was added pomalidomide 10 (0.3 mmol, 1.0 equiv.), NaOAc·3H_2_O (0.45 mmol, 1.5 equiv.), subsequently treated DMF (3 mL, *c* = 0.1 M) was added to vial *via* syringe, the reaction mixture was stirred for 10 min at 22 °C. Then propargyl sulfonium salt 2a (0.45 mmol, 1.5 equiv.) was added in one portion. The reaction was stirred at 50 °C for 12 h until pomalidomide 10 was fully consumed (monitored by TLC). The organic solvent was removed under reduced pressure and purified through column chromatography (eluent: petroleum ether and EtOAc) to afford the desired product 11.

#### 3-Amino-2-(1-(3-(methylthio)prop-1-en-2-yl)-2,6-dioxopiperidin-3-yl)isoindoline-1,3-dione (11)

The reaction was carried out on a 0.3 mmol scale by following the general procedure. The product 11 was purified through column chromatography (PE/EtOAc from 8 : 1 to 1 : 1) as colorless oil (58 mg, 80% yield). IR *ν*_max_ (neat)/cm^−1^: 3432, 3397, 2958, 2925, 2851, 1725, 1681, 1378, 1256, 1191, 1107, 718; ^1^H NMR (600 MHz, CDCl_3_): *δ* 7.43 (t, *J* = 7.8 Hz, 1H), 7.16 (d, *J* = 7.2 Hz, 1H), 6.88 (d, *J* = 8.4 Hz, 1H), 5.57 (s, 1H), 5.28 (s, 2H), 5.23 (s, 1H), 5.31 (dd, *J* = 4.8, 12.0 Hz, 1H), 3.33 (dd, *J* = 15.0, 21.6 Hz, 2H), 3.06–2.98 (m, 1H), 2.90–2.82 (m, 2H), 2.16–2.15 (m, 1H), 2.09 (s, 3H); ^13^C NMR (150 MHz, CDCl_3_): *δ* 170.5, 168.9, 168.3, 167.5, 145.6, 136.9, 135.5, 132.3, 121.4, 118.4, 113.1, 110.8, 49.6, 37.5, 32.1, 22.1, 15.3. HRMS (ESI, *m*/*z*): calcd for C_17_H_18_N_3_O_4_S^+^, [M + H]^+^, 360.1013, found 360.1016.

To a flame-dried 100 mL round bottom flask equipped with a stir bar was added phthalimide 1a (1.01 g, 6.8 mmol, 1.0 equiv.), NaOAc·3H_2_O (1.39 g, 10.2 mmol, 1.5 equiv.), subsequently treated CH_3_CN (68 mL, *c* = 0.1 M) was added to vial *via* syringe, the reaction mixture was stirred for 10 min at 22 °C. Then propargyl sulfonium salt 2a (1.85 g, 10.2 mmol, 1.5 equiv.) was added in one portion. The reaction was stirred at 50 °C for 10 h until phthalimide 1a was fully consumed (monitored by TLC). The organic solvent was removed under reduced pressure and purified through column chromatography (eluent: petroleum ether and EtOAc) to afford the desired product 3 with yield of 65% (1.03 g).

To a flame-dried sealable 2-dram vial equipped with a stir bar was added phthalimide 3a (50 mg, 0.2 mmol, 1.0 equiv.), Na_2_CO_3_ (42.4 mg, 0.4 mmol, 2.0 equiv.) and CH_2_Cl_2_ (3 mL, *c* = 0.2 M). After stirred at 0 °C for 2 min, *m*-CPBA (103.5 mg, 0.6 mmol, 75%, 3.0 equiv.) was added in portions to the mixture. The reaction mixture was kept stirring for 10 min at 0 °C until 3a was fully consumed (monitored by TLC). The reaction was quenched with aqueous Na_2_CO_3_ and extracted with CH_2_Cl_2_ (5 mL *×* 3). The combined organic solvent was dried with anhydrous Na_2_SO_4_, removed under reduced pressure and purified through column chromatography (eluent: petroleum ether and EtOAc) to afford the desired product 12 (50 mg, 94%).

#### 2-(3-(Methylsulfonyl)prop-1-en-2-yl)isoindoline-1,3-dione (12)

The product 12 was purified through column chromatography (eluent: petroleum ether and EtOAc) as colorless oil (50 mg, 94% yield). IR *ν*_max_ (neat)/cm^−1^: 3486, 3416, 3129, 3023, 2995, 2925, 1776, 1720, 1648, 1362, 1311, 1123, 1080, 877, 722, 485; ^1^H NMR (600 MHz, CDCl_3_): *δ* 7.91 (d, *J* = 3.0 Hz, 2H), 7.79 (dd, *J* = 3.0, 5.4 Hz, 2H), 5.77 (s, 1H), 5.66 (s, 1H), 4.27 (s, 2H), 3.00 (s, 3H); ^13^C NMR (150 MHz, CDCl_3_): *δ* 166.7, 134.6, 131.5, 127.9, 124.0, 122.8, 57.5, 39.3. HRMS (ESI, *m*/*z*): calcd for C_12_H_12_NO_4_S^+^, [M + H]^+^, 266.0482, found 266.0481.

## Conflicts of interest

The authors declare no conflicts of interest.

## Supplementary Material

RA-012-D2RA01117D-s001
