# Supplementary material for: Construction of sulfur-containing N-vinylimides: N-addition of imides to propargyl sulfonium salts
Source: RSC Adv. 2022 Apr 26;12(20):12663–71. doi: 10.1039/d2ra01117d (PMC9039989; doi:10.1039/d2ra01117d)

Electronic Supplementary Material (ESI) for RSC Advances.
This journal is © The Royal Society of Chemistry 2022

Construction of Sulfur-containing N-Vinylimides: N-Addition of Imides with Propargyl Sulfonium Salts

Shou-Jie Shen,*[,a] Le-Mei Wang,[a] Guo-Mei Gong,[a] Yan-Jiao Wang,[a] Jin-Yan Liang*[,b] and Jun-Wen Wang*[,a]

† Key Laboratory of Magnetic Molecules, Magnetic Information Materials Ministry of Education, The School of Chemical and Material Science, Shanxi Normal University, Linfen, 041004, China

‡ College of Life Science, Shanxi Normal University, Linfen, 041004, China

Email: shoujie_shen@outlook.com, jinyan_liang@outlook.com, wangjunwen2013@126.com

Supporting Material

A. General Information……………………………………………………..……..S2

B. Effect of Parameters…………………………..…………………..………....S3

C. NMR Spectra…………………………………………..……….….…S4-S32

A.General Information

General Procedures. All reactions were performed in oven-dried or flame-dried round-bottom flasks and vials. Stainless steel syringes and cannula were used to transfer air- and moisture-sensitive liquids. Flash chromatography was performed using silica gel 60 (230−400 mesh) from Aladdin.

Materials. Commercial reagents were purchased from TCI, Aladdin and J&K and used as received. All solvents were used after being freshly distilled unless otherwise noted. Analytical thin layer chromatography (TLC) was performed on percolated glass backed plates (silica gel 60 F254; 0.25 mm thickness). The TLC plates were visualized by UV illumination and by staining.

Instrumentation. Proton nuclear magnetic resonance (^{1}H NMR) spectra and carbon nuclear magnetic resonance (^{13}C NMR) spectra were recorded on Bruker UltraShield–600 (600 MHz). Chemical shifts for protons are reported in parts per million downfield from tetramethylsilane and are referenced to the NMR solvent residual peak ($CHCl_3$ δ 7.26). Chemical shifts for carbons are reported in parts per million downfield from tetramethylsilane and are referenced to the carbon resonances of the NMR solvent ($CDCl_3$ δ 77.0). Data are represented as follows: chemical shift, multiplicity (br = broad, s = singlet, d = doublet, t = triplet, q = quartet, m = multiplet), coupling constants in Hertz (Hz), and integration. The mass spectroscopic data were obtained using a Micromass Platform II single quadrupole instrument. Infrared (IR) spectra were obtained as thin films on KBr plates by dissolving the compound in CH_2Cl_2 followed by evaporation. Data are represented as follows: frequency of absorption (cm^{-1}) and absorption strength (s = strong, m = medium, w = weak).

Abbreviations Used: THF–tetrahydrofuran, TEA–triethylamine, MeCN–acetonitrile, MS–molecular sieves, DMF–N,N′-Dimethylformamide, TLC–thin layer chromatography, DCE−1,2-dichloroethane, EtOAc–ethyl acetate.

B. Effect of Parameters

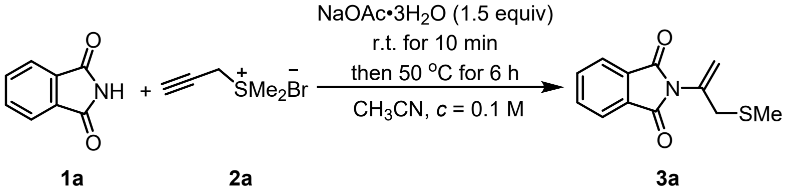

General Procedure : To a flame-dried sealable 3-dram vial equipped with a stir bar was added imides **1a** (0.3 mmol, 1.0 equiv), $NaOAc\cdot 3H_2O$ (0.45 mmol, 1.5 equiv), subsequently treated CH_3CN (3.0 mL, c = 0.1 M) was added to vial via syringe, the reaction mixture was stirred for 10 min at 22 °C. Then propargyl sulfonium salt **2a** (0.45 mmol, 1.5 equiv) was added in one portion. The reaction was stirred at 50 °C for 6 h until imide **1a** was fully consumed (monitored by TLC). The organic solvent was removed under reduced pressure and purified through column chromatography (eluent: petroleum ether and EtOAc) to afford the desired product **3a**.We probed the influence of the ratio of reactants and reaction temperature(Table 1).

Table 1. Optimization of the Reaction Conditions

Entry	Base	Solvent	1a:2a:base	Temp (°C)	Yield(%)
**1**	$NaOAc\cdot 3H_2O$	CH_3CN	1: 1.3: 2.5	50	48
**2**	$NaOAc\cdot 3H_2O$	CH_3CN	1: 1.5: 2.5	50	57
**3**	$NaOAc\cdot 3H_2O$	CH_3CN	1: 1.5: 1.5	50	71
**4**	$NaOAc\cdot 3H_2O$	CH_3CN	1: 1.5: 2.0	50	49
**5**	$NaOAc\cdot 3H_2O$	CH_3CN	1: 2.0: 1.5	50	61

C. NMR Spectra

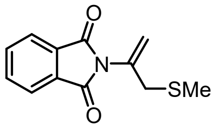

3a
$CDCl_3$, 600 MHz

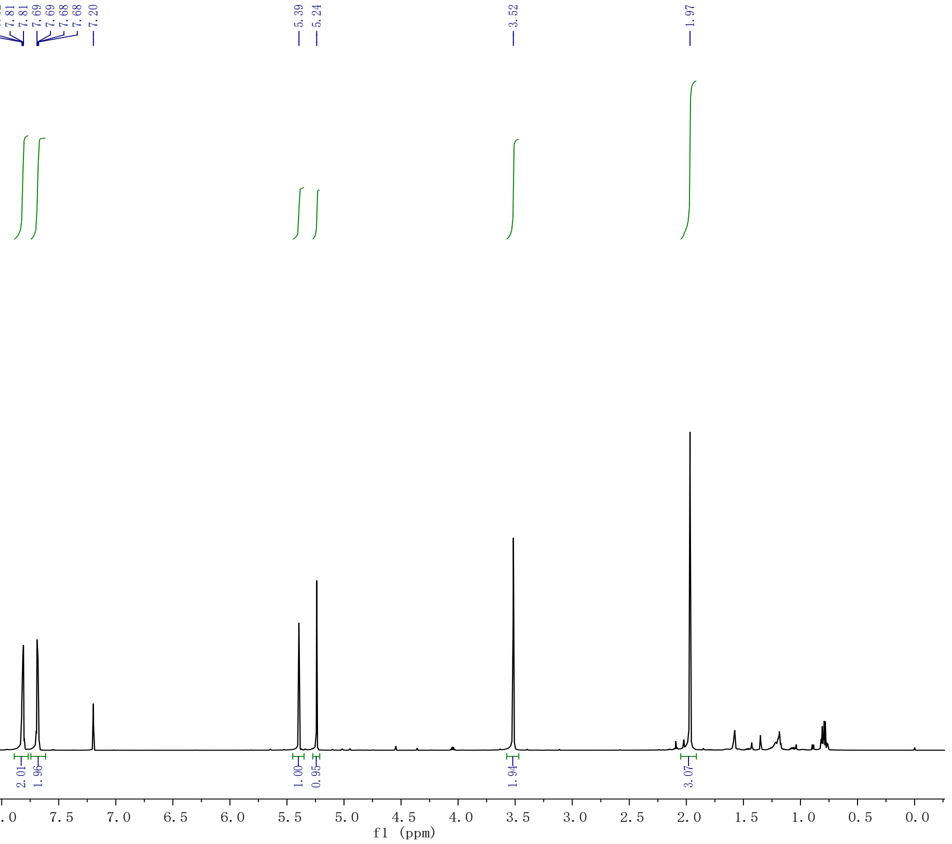

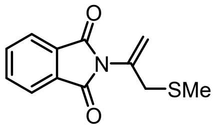

3a
$CDCl_3$, 150 MHz

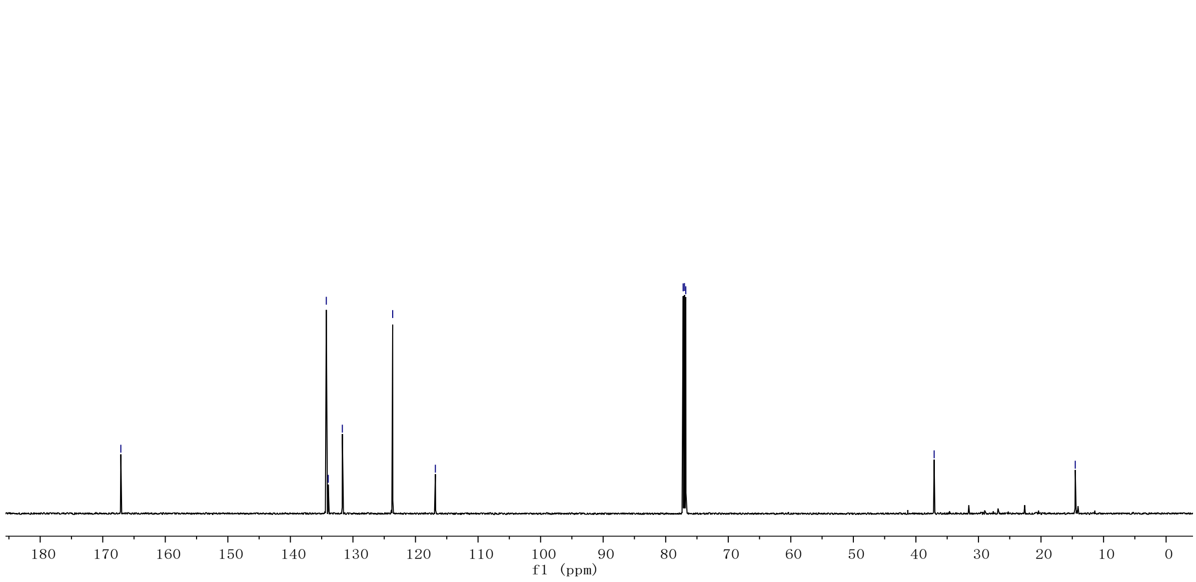

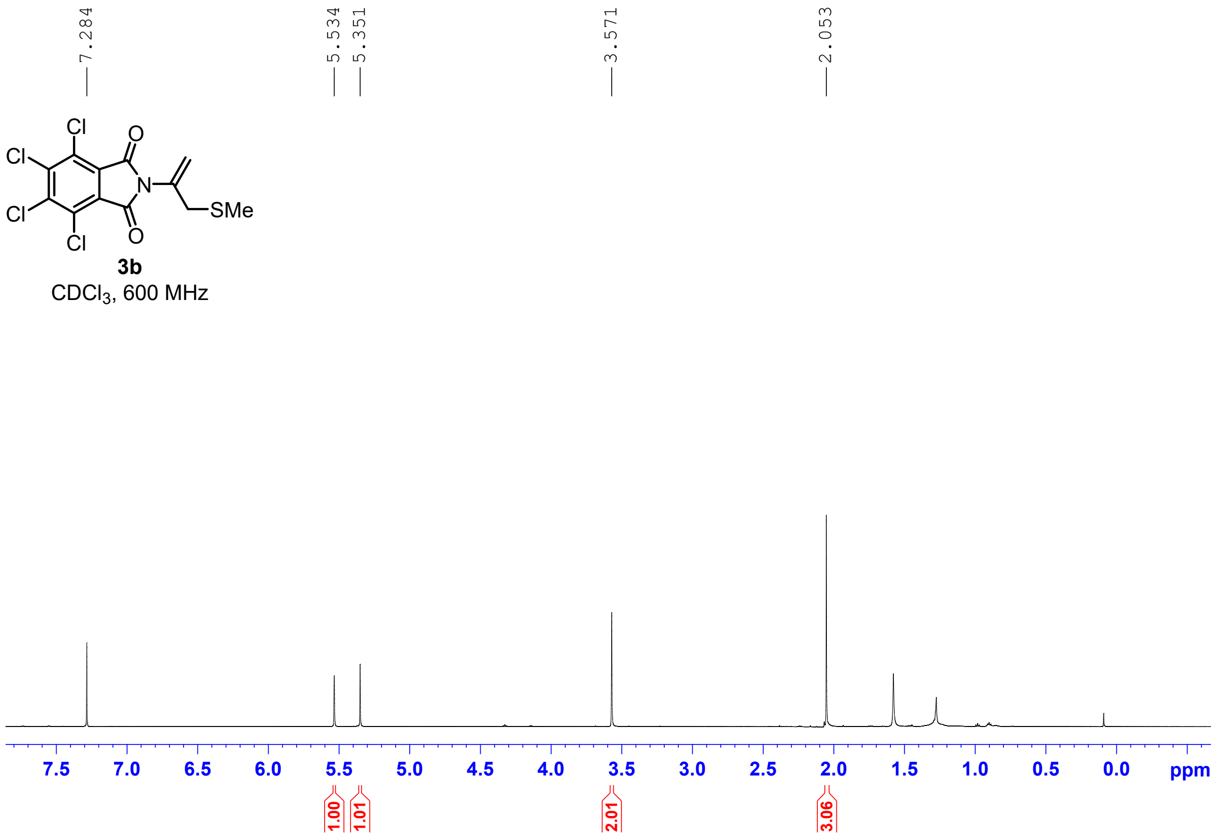
7.284
5.534
5.351
3.571
2.053
Cl
Cl
Cl
Cl
O
O
N
SMe
3b
CDCl3, 600 MHz
7.5
7.0
6.5
6.0
5.5
5.0
4.5
4.0
3.5
3.0
2.5
2.0
1.5
1.0
0.5
0.0
ppm
1.00
1.01
2.01
3.06

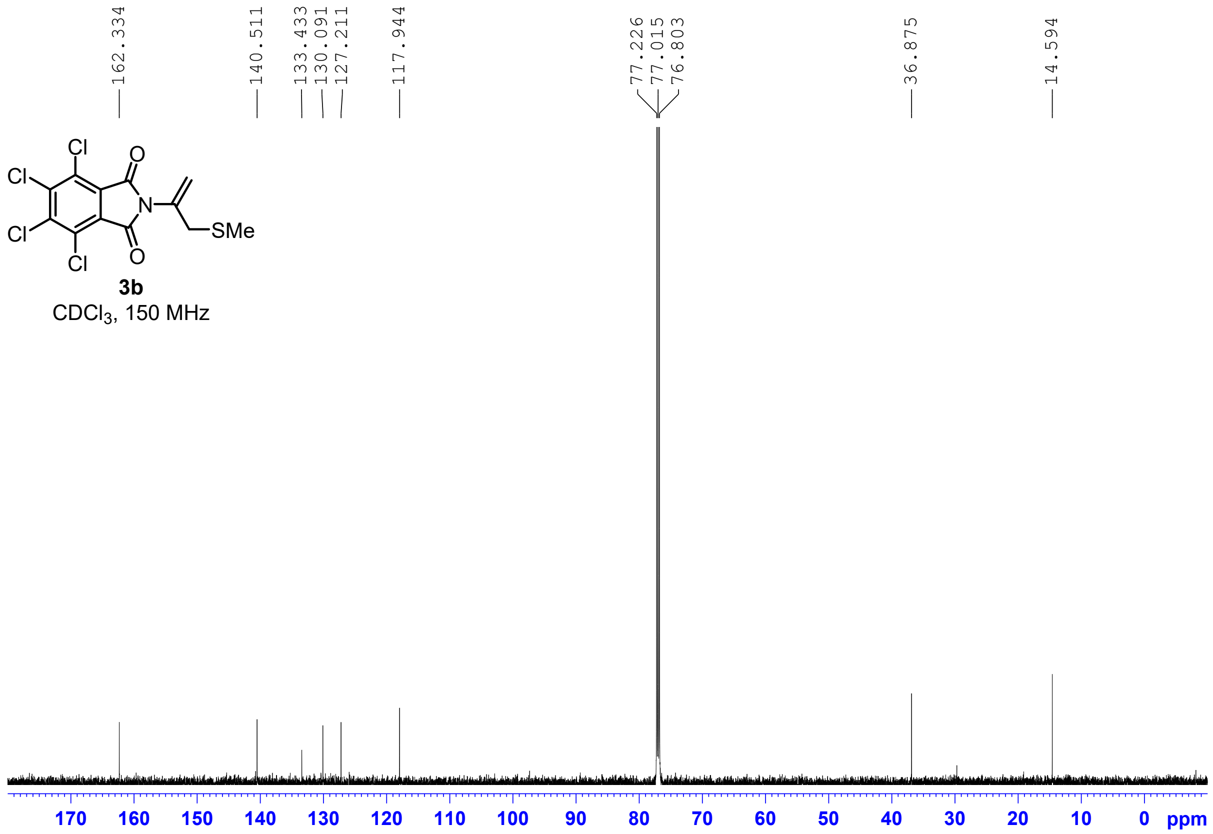
162.334
140.511
133.433
130.091
127.211
117.944
77.226
77.015
76.803
36.875
14.594
Cl
Cl
Cl
Cl
O
O
N
SMe
3b
CDCl3, 150 MHz
170
160
150
140
130
120
110
100
90
80
70
60
50
40
30
20
10
0
ppm

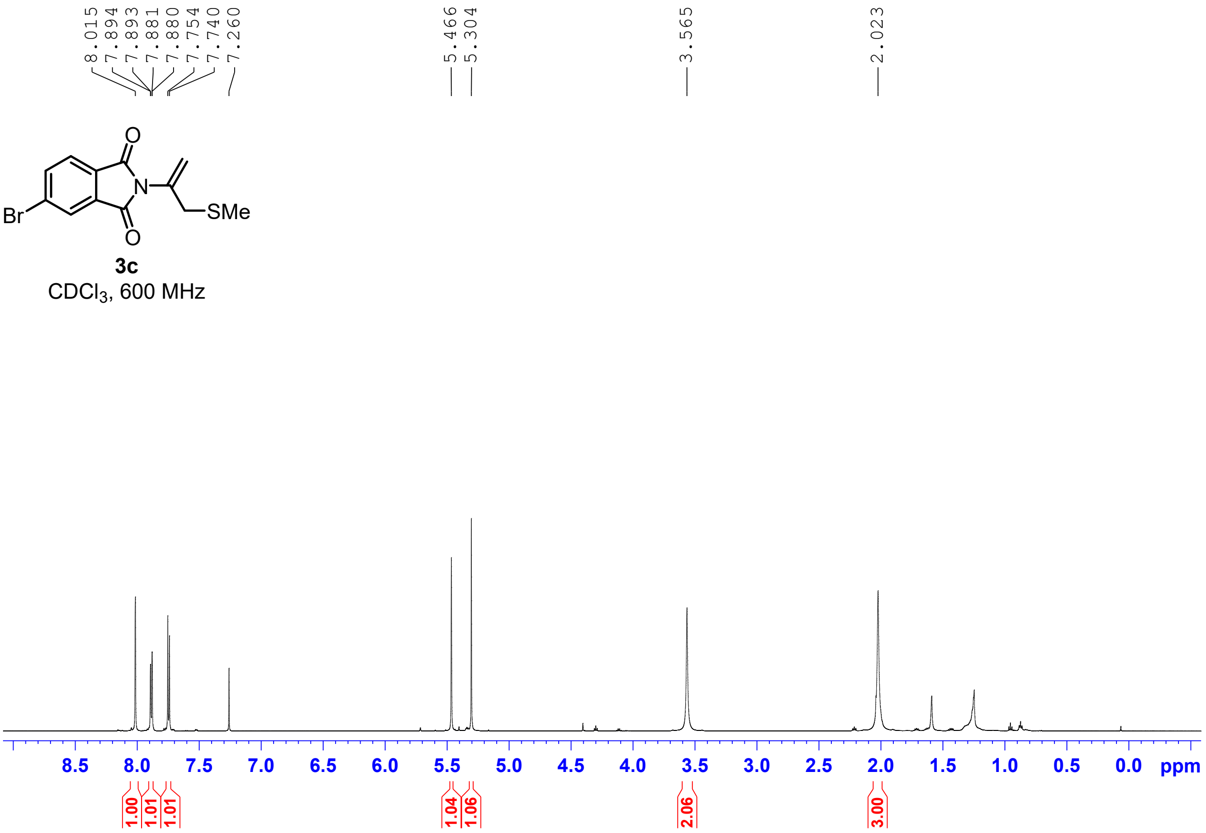
8.015
7.894
7.893
7.881
7.880
7.754
7.740
7.260
5.466
5.304
3.565
2.023
Br
O
N
SMe
3c
CDCl3, 600 MHz
1.00
1.01
1.01
1.04
1.06
2.06
3.00
ppm

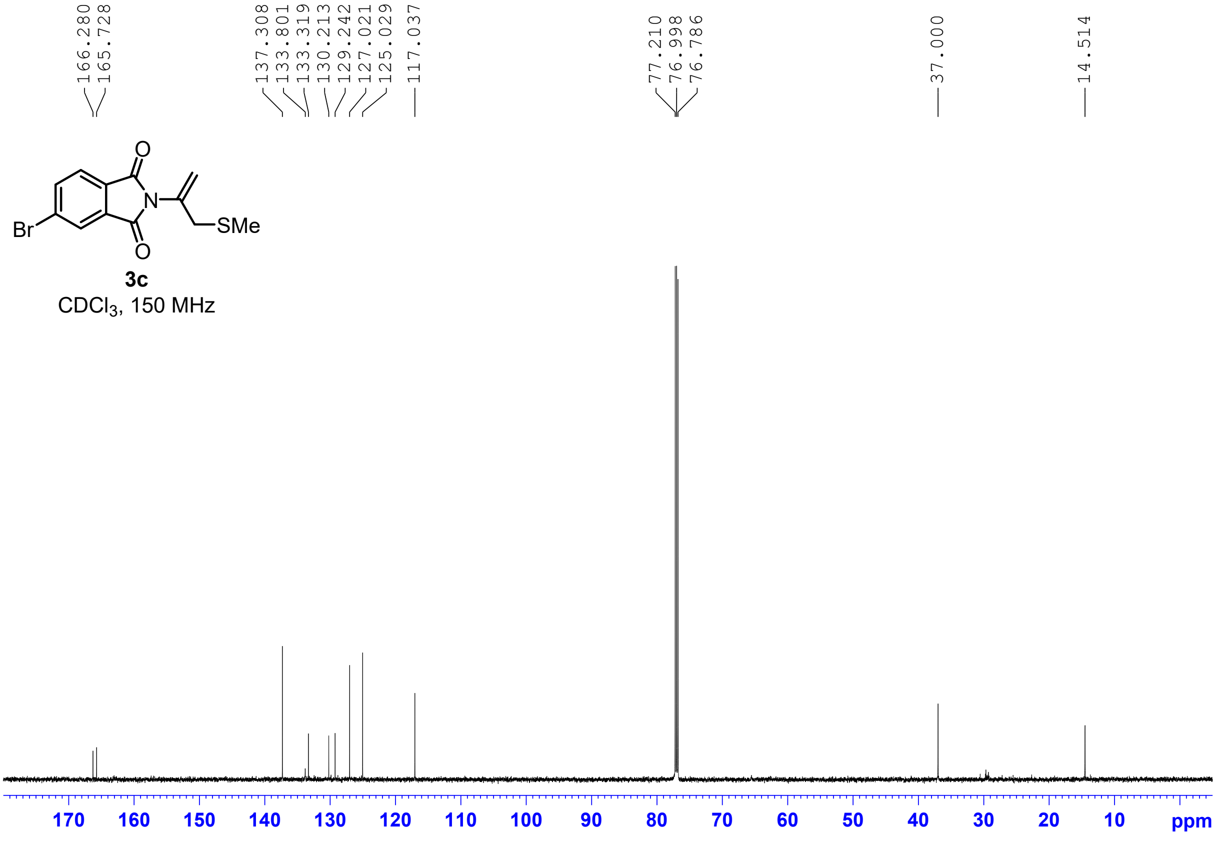
166.280
165.728
137.308
133.801
133.319
130.213
129.242
127.021
125.029
117.037
77.210
76.998
76.786
37.000
14.514
Br
O
N
SMe
3c
CDCl3, 150 MHz
ppm

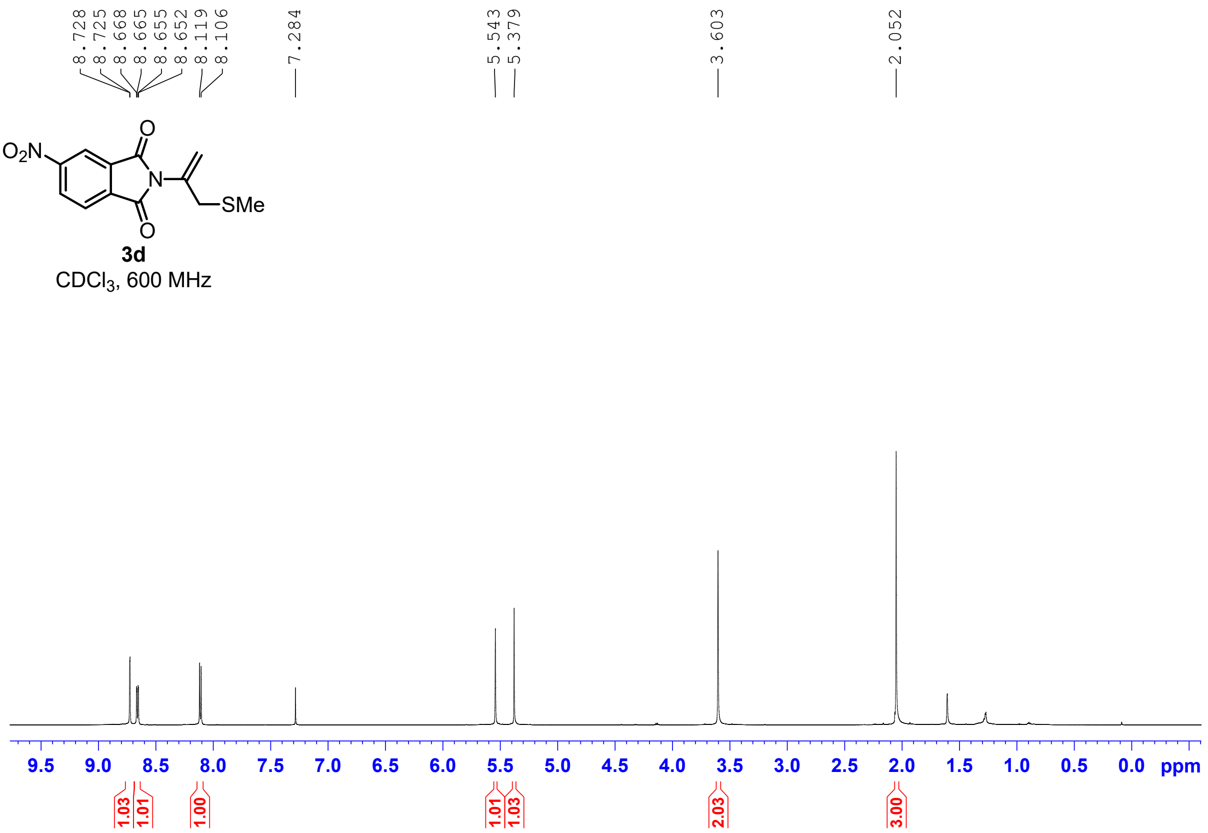
8.728
8.725
8.668
8.665
8.655
8.652
8.119
8.106
7.284
5.543
5.379
3.603
2.052
O_2N
N
O
O
SMe
3d
$CDCl_3$, 600 MHz
1.03
1.01
1.00
1.01
1.03
2.03
3.00
9.5 9.0 8.5 8.0 7.5 7.0 6.5 6.0 5.5 5.0 4.5 4.0 3.5 3.0 2.5 2.0 1.5 1.0 0.5 0.0 ppm

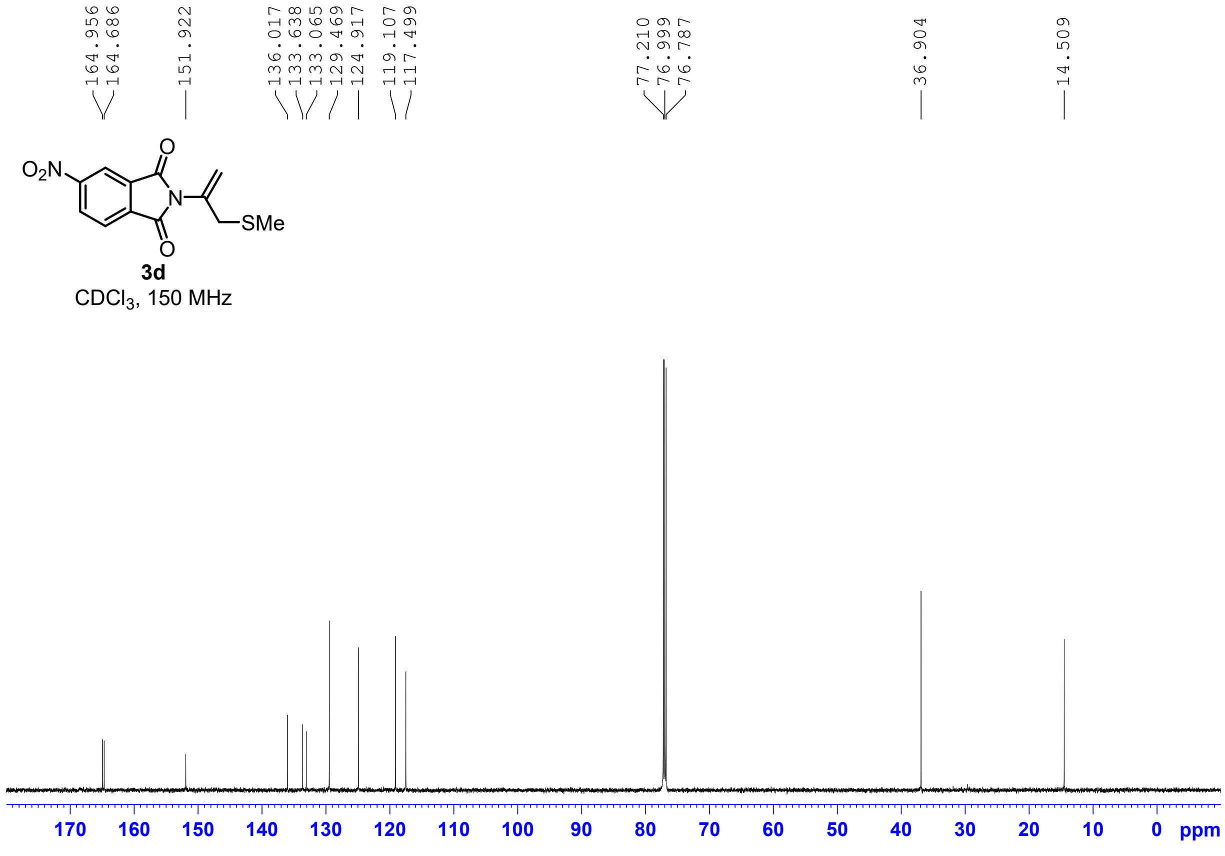
164.956
164.686
151.922
136.017
133.638
133.065
129.469
124.917
119.107
117.499
77.210
76.999
76.787
36.904
14.509
O_2N
N
O
O
SMe
3d
$CDCl_3$, 150 MHz
170 160 150 140 130 120 110 100 90 80 70 60 50 40 30 20 10 0 ppm

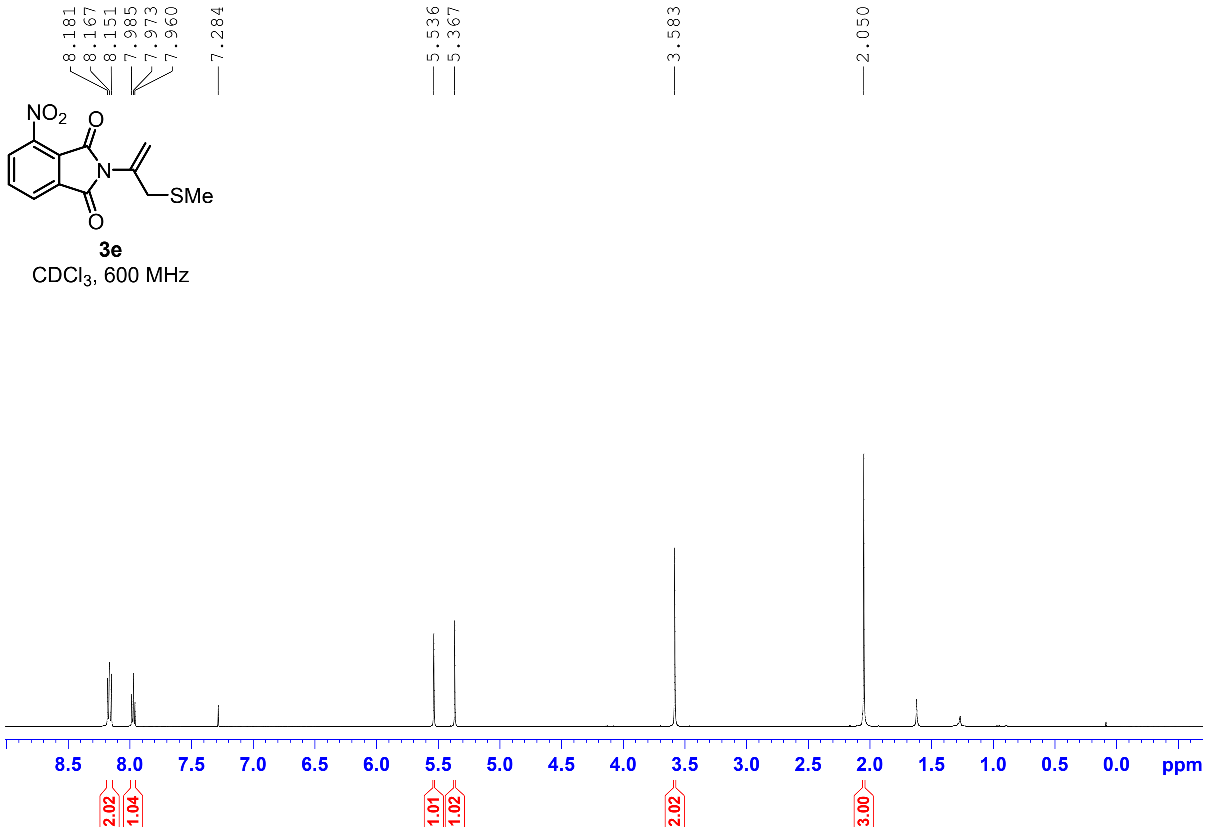
8.181
8.167
8.151
7.985
7.973
7.960
7.284
5.536
5.367
3.583
2.050
NO2
O
N
SMe
O
3e
CDCl3, 600 MHz
8.5 8.0 7.5 7.0 6.5 6.0 5.5 5.0 4.5 4.0 3.5 3.0 2.5 2.0 1.5 1.0 0.5 0.0 ppm
2.02
1.04
1.01
1.02
2.02
3.00

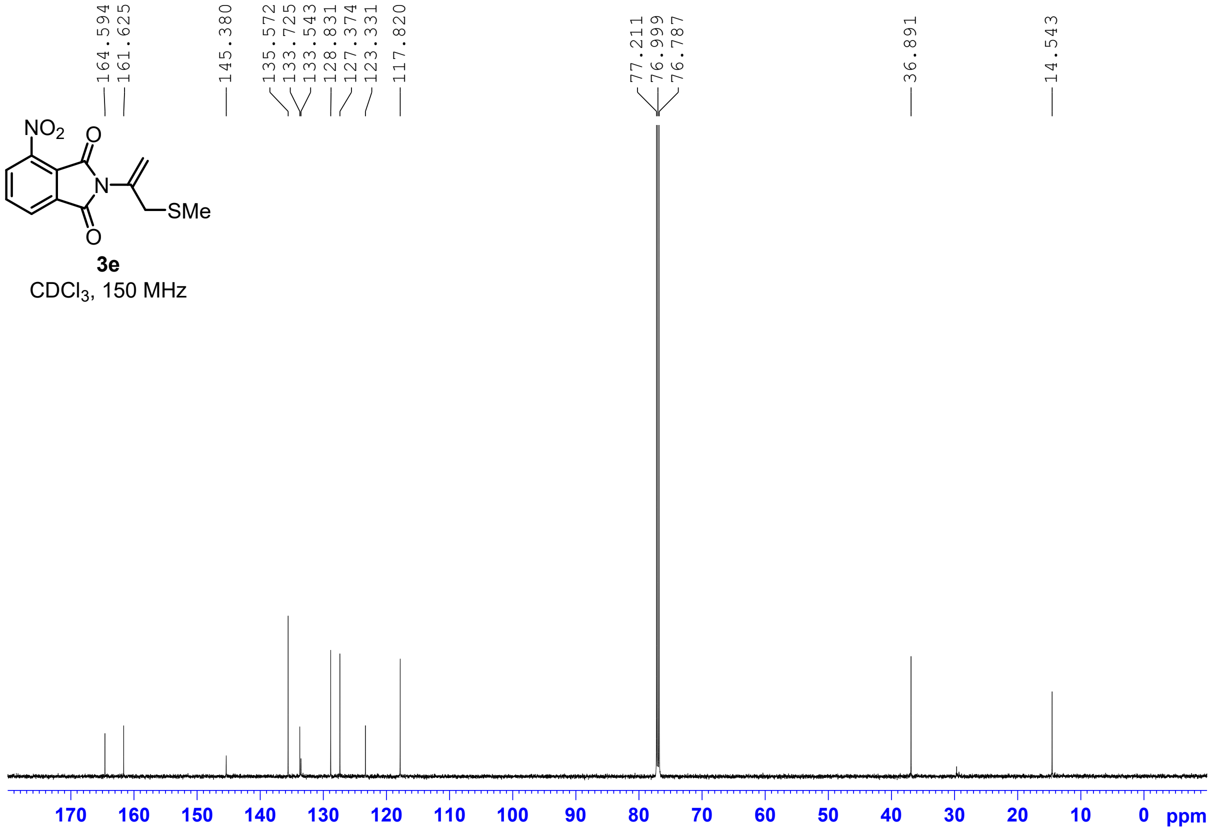
164.594
161.625
145.380
135.572
133.725
133.543
128.831
127.374
123.331
117.820
77.211
76.999
76.787
36.891
14.543
NO2
O
N
SMe
O
3e
CDCl3, 150 MHz
170 160 150 140 130 120 110 100 90 80 70 60 50 40 30 20 10 0 ppm

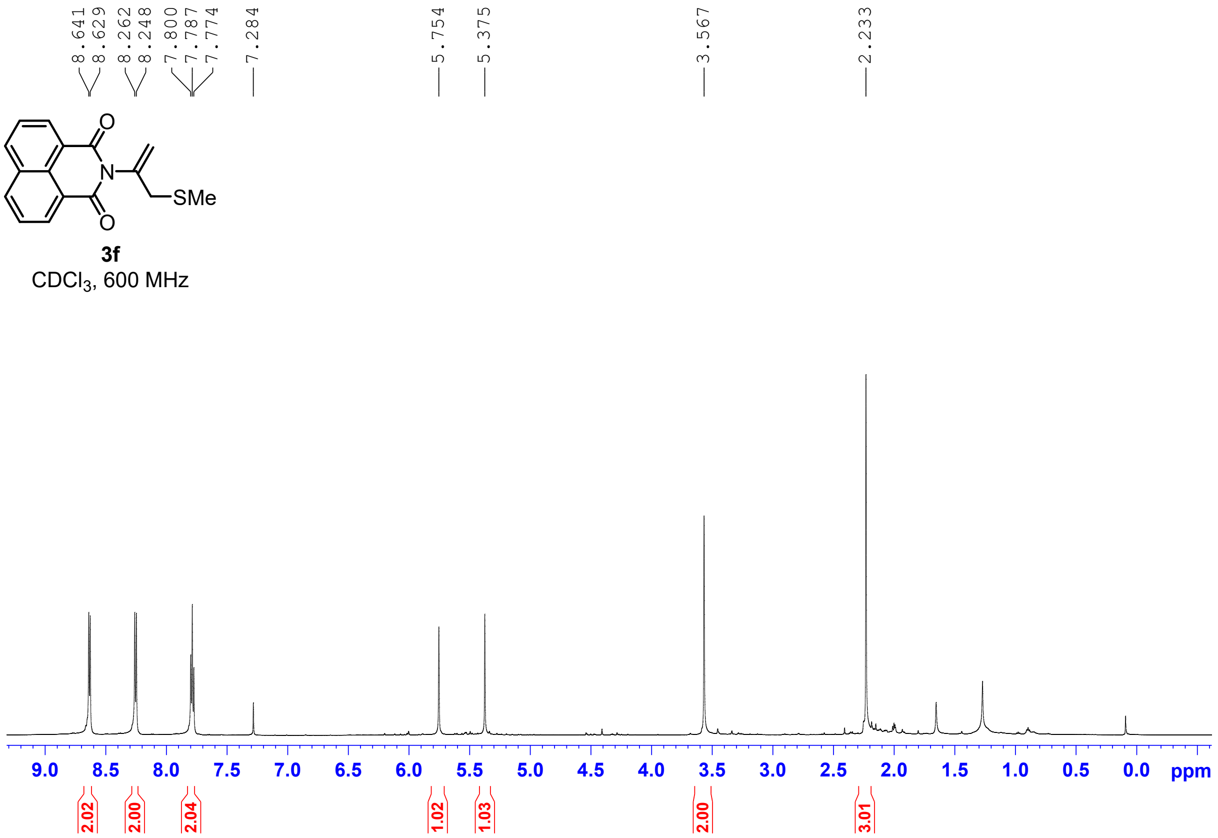
8.641
8.629
8.262
8.248
7.800
7.787
7.774
7.284
5.754
5.375
3.567
2.233
SMe
O
N
O
3f
CDCl3, 600 MHz
9.0 8.5 8.0 7.5 7.0 6.5 6.0 5.5 5.0 4.5 4.0 3.5 3.0 2.5 2.0 1.5 1.0 0.5 0.0 ppm
2.02
2.00
2.04
1.02
1.03
2.00
3.01

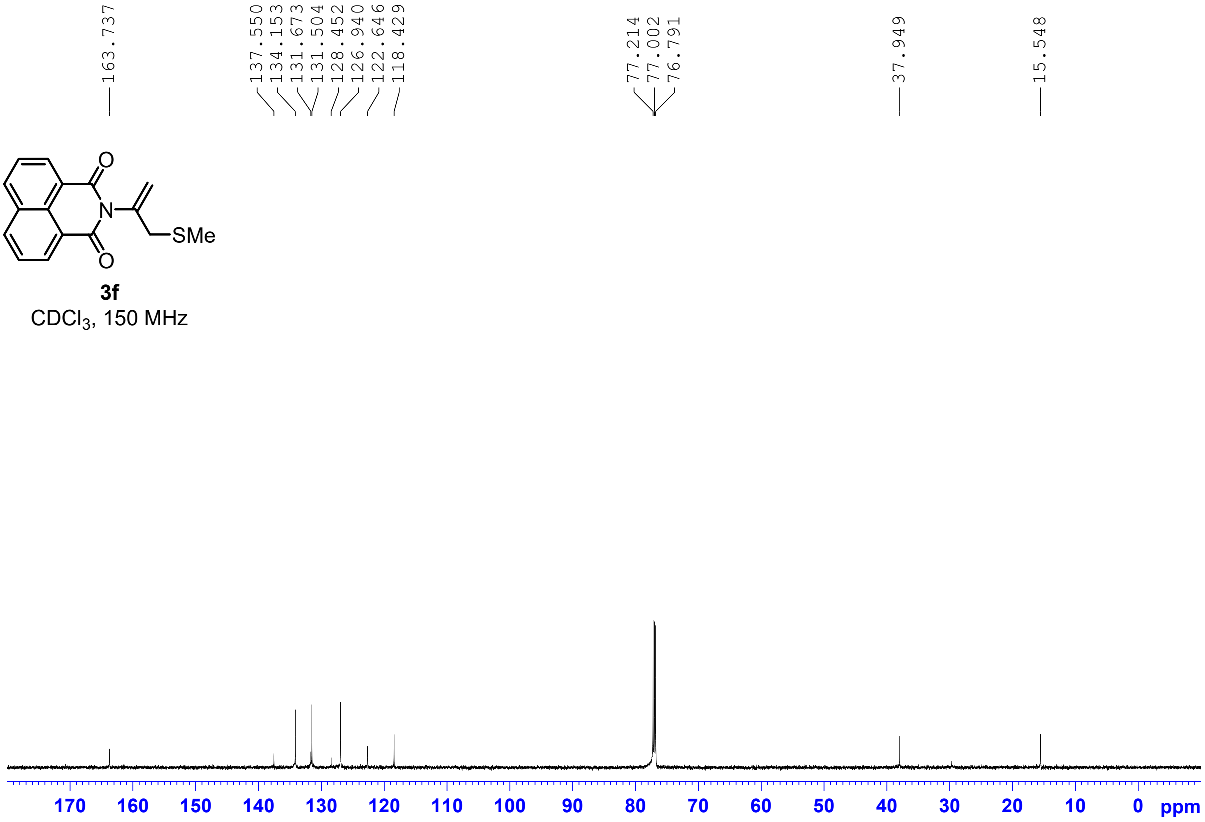
163.737
137.550
134.153
131.673
131.504
128.452
126.940
122.646
118.429
77.214
77.002
76.791
37.949
15.548
SMe
O
N
O
3f
CDCl3, 150 MHz
170 160 150 140 130 120 110 100 90 80 70 60 50 40 30 20 10 0 ppm

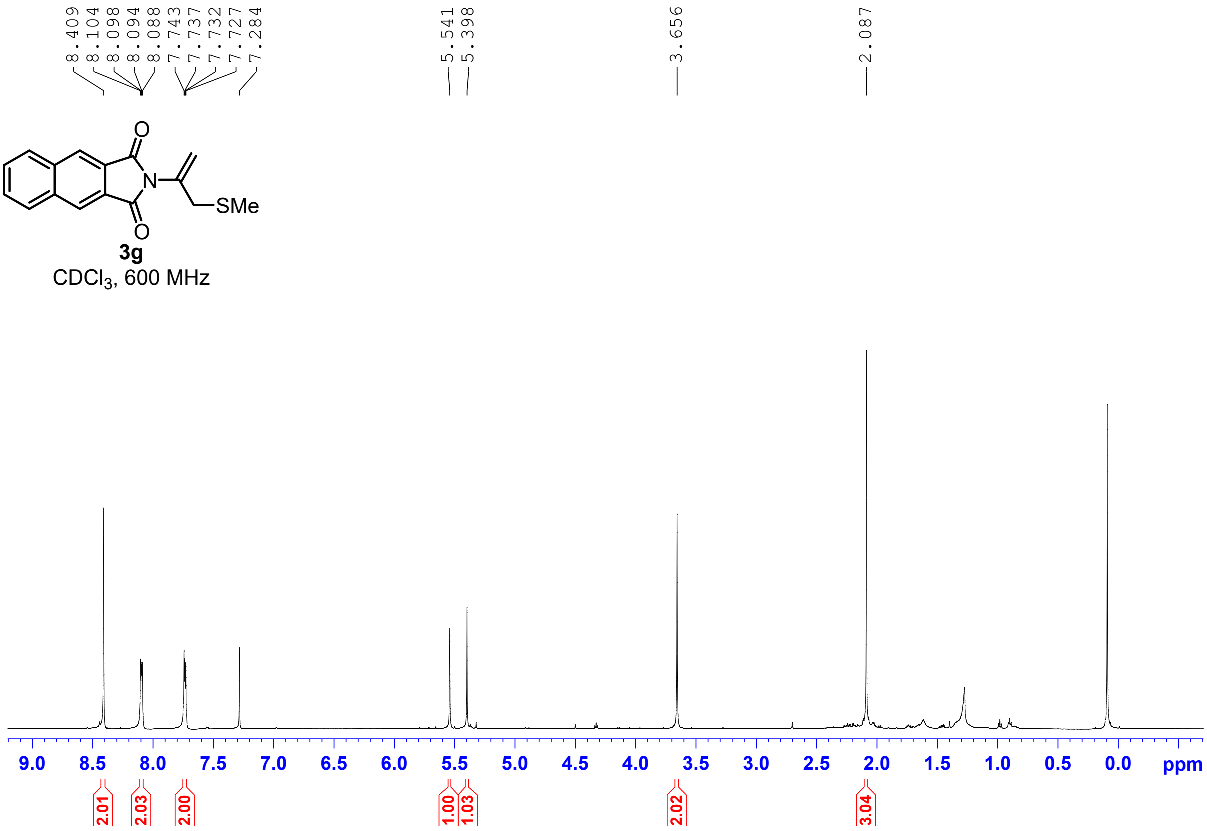
8.409
8.104
8.098
8.094
8.088
7.743
7.737
7.732
7.727
7.284
5.541
5.398
3.656
2.087
O
N
SMe
O
3g
CDCl3, 600 MHz
9.0 8.5 8.0 7.5 7.0 6.5 6.0 5.5 5.0 4.5 4.0 3.5 3.0 2.5 2.0 1.5 1.0 0.5 0.0 ppm
2.01
2.03
2.00
1.00
1.03
2.02
3.04

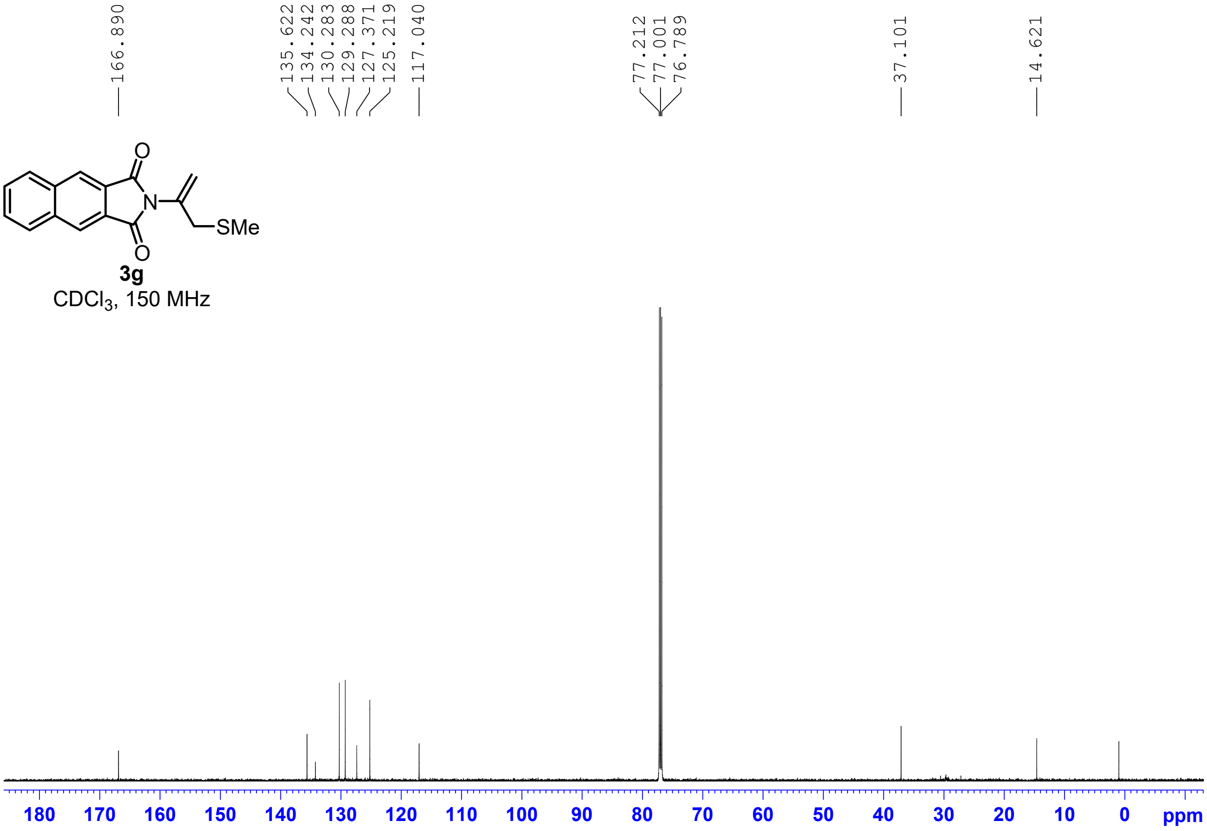
166.890
135.622
134.242
130.283
129.288
127.371
125.219
117.040
77.212
77.001
76.789
37.101
14.621
O
N
SMe
O
3g
CDCl3, 150 MHz
180 170 160 150 140 130 120 110 100 90 80 70 60 50 40 30 20 10 0 ppm

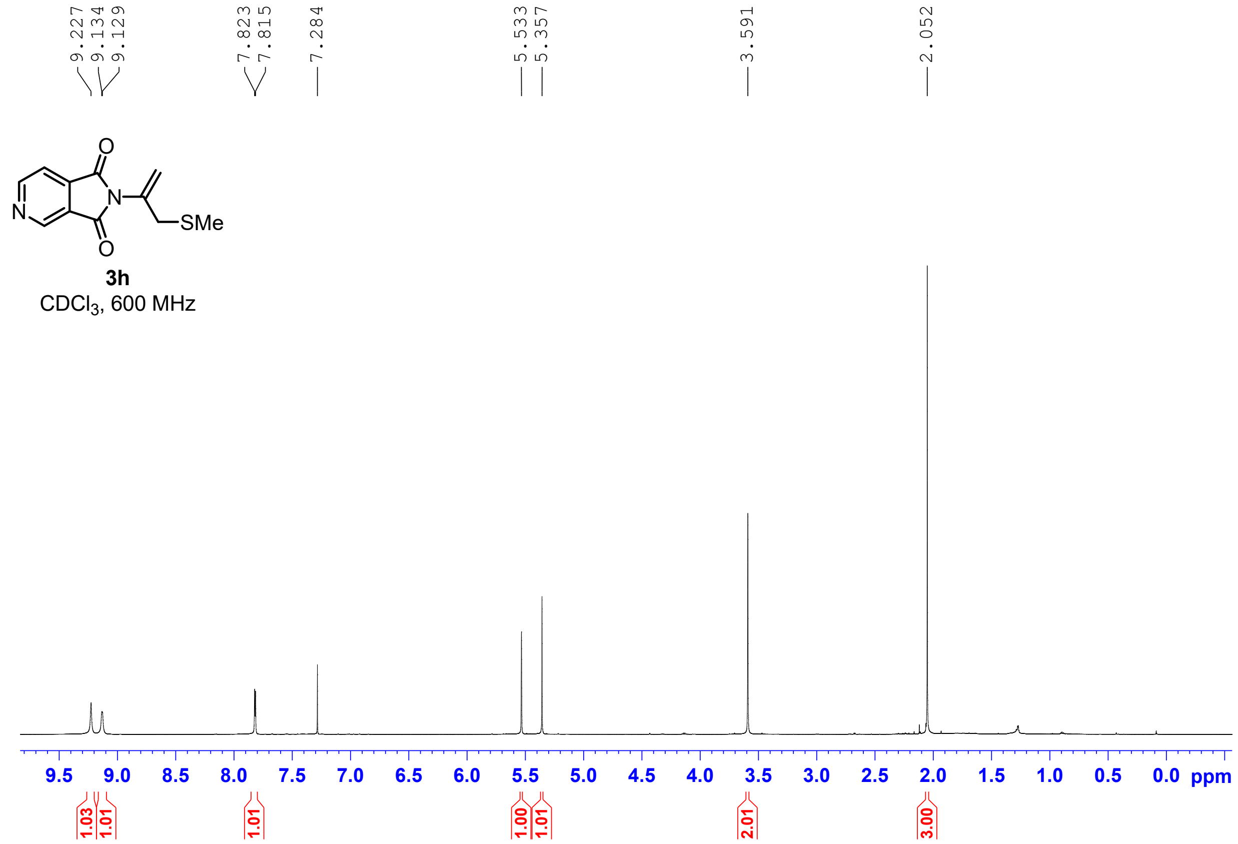

9.227
9.134
9.129
7.823
7.815
7.284
5.533
5.357
3.591
2.052
N
O
N
O
SMe
3h
CDCl3, 600 MHz
9.5 9.0 8.5 8.0 7.5 7.0 6.5 6.0 5.5 5.0 4.5 4.0 3.5 3.0 2.5 2.0 1.5 1.0 0.5 0.0 ppm
1.03
1.01
1.01
1.00
1.01
2.01
3.00

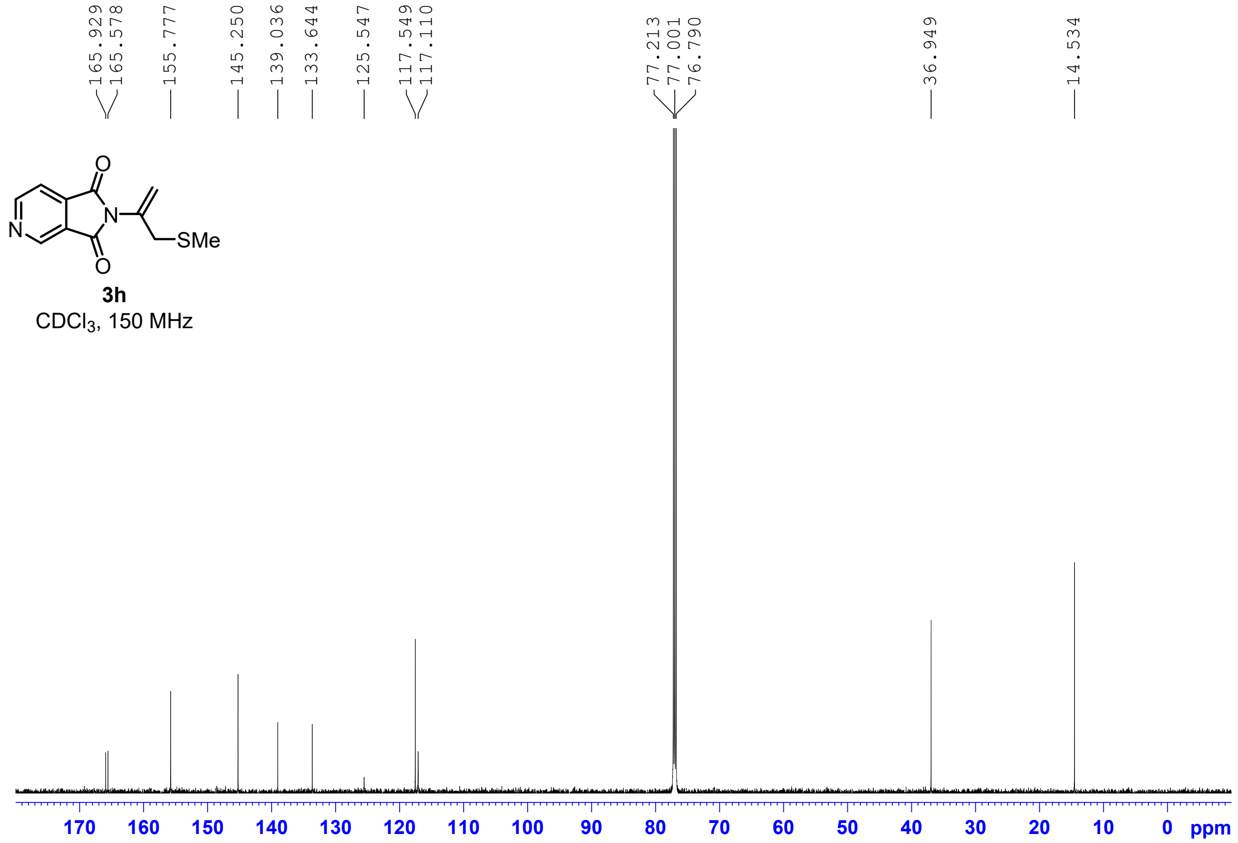

165.929
165.578
155.777
145.250
139.036
133.644
125.547
117.549
117.110
77.213
77.001
76.790
36.949
14.534
N
O
N
O
SMe
3h
CDCl3, 150 MHz
170 160 150 140 130 120 110 100 90 80 70 60 50 40 30 20 10 0 ppm

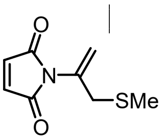

3i

$CDCl_3$, 600 MHz

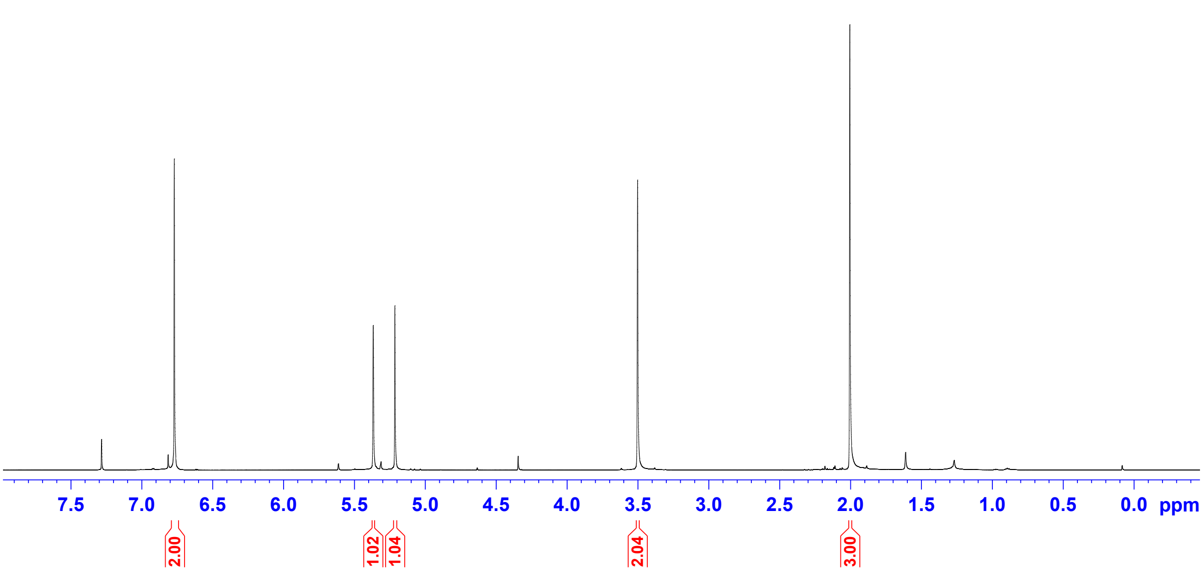

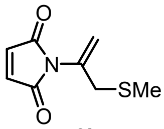

3i

$CDCl_3$, 150 MHz

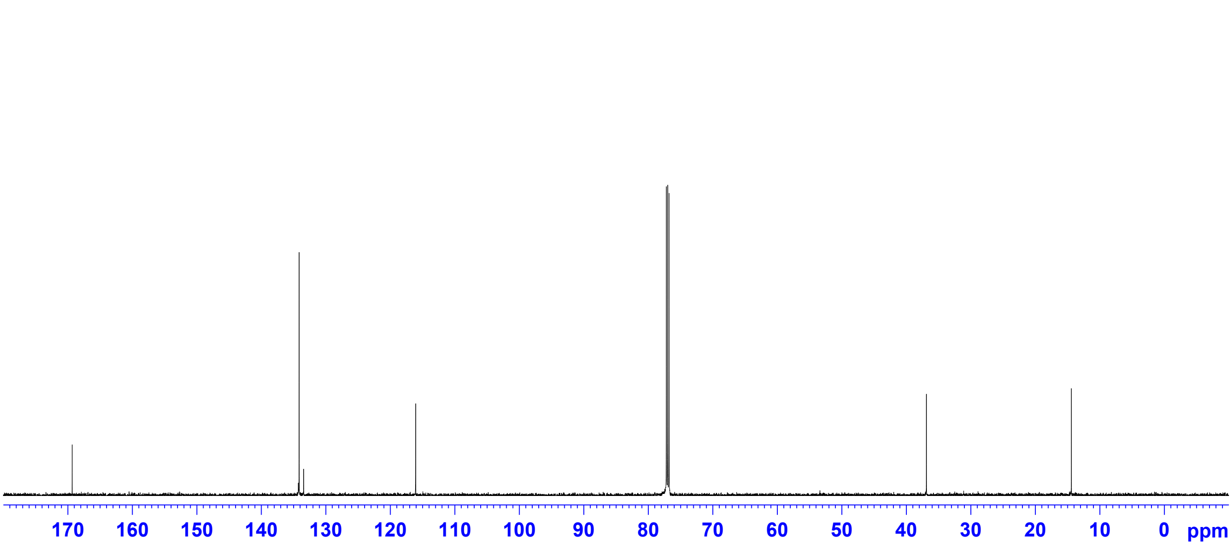

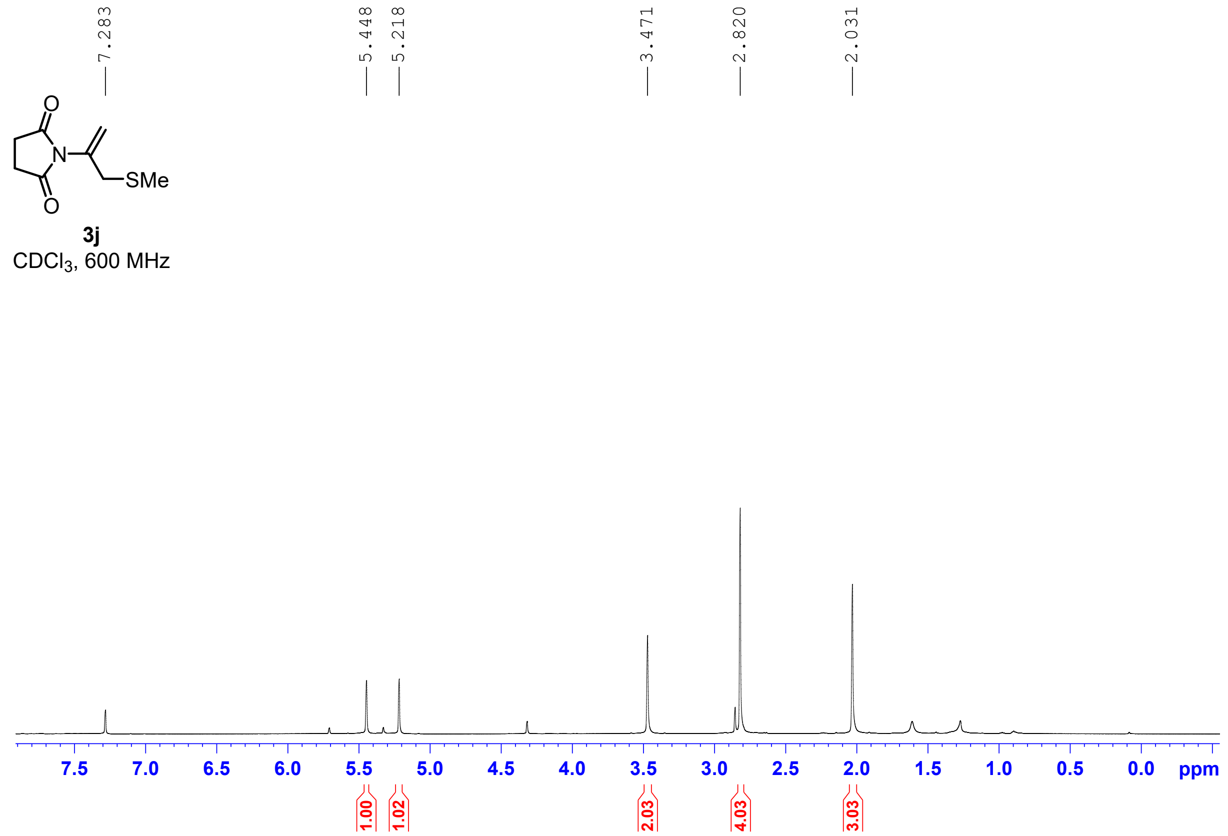
7.283
5.448
5.218
3.471
2.820
2.031
SMe
3j
CDCl3, 600 MHz
1.00
1.02
2.03
4.03
3.03
ppm

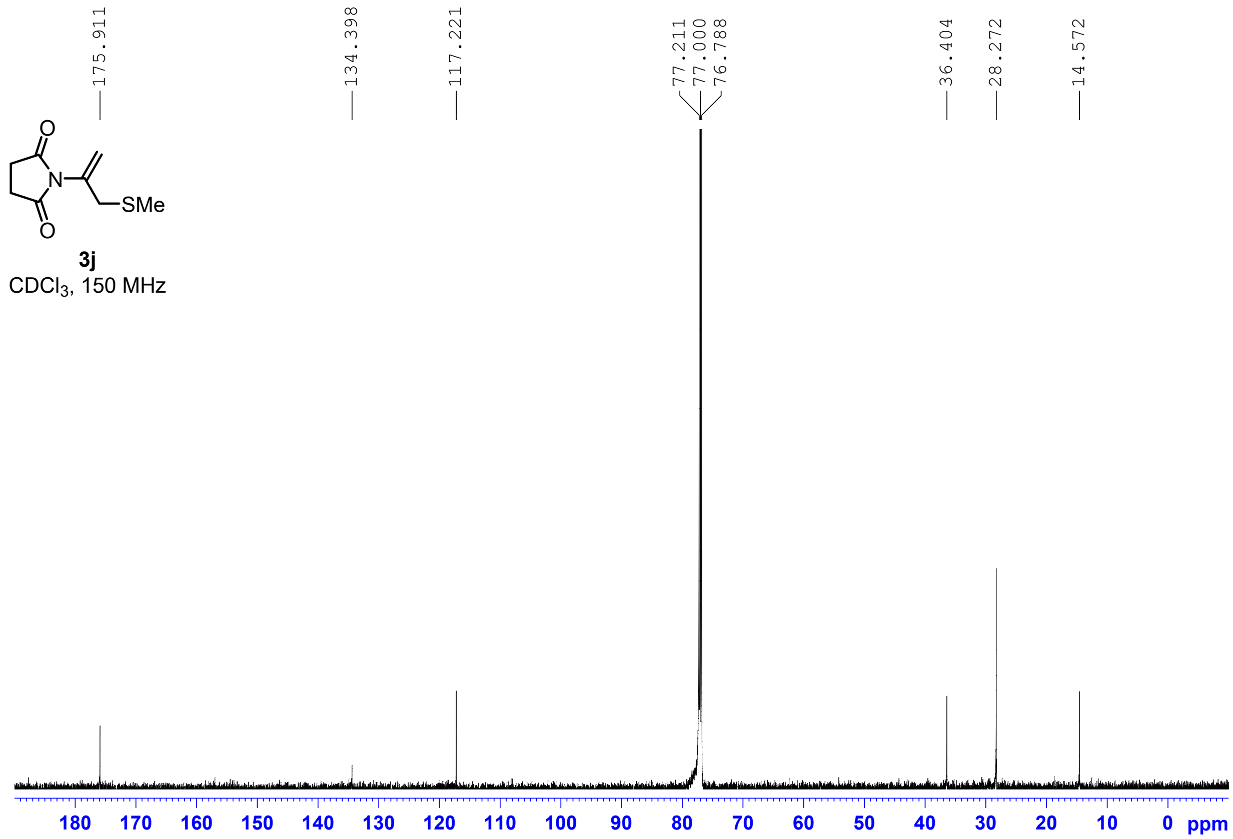
175.911
134.398
117.221
77.211
77.000
76.788
36.404
28.272
14.572
SMe
3j
CDCl3, 150 MHz
ppm

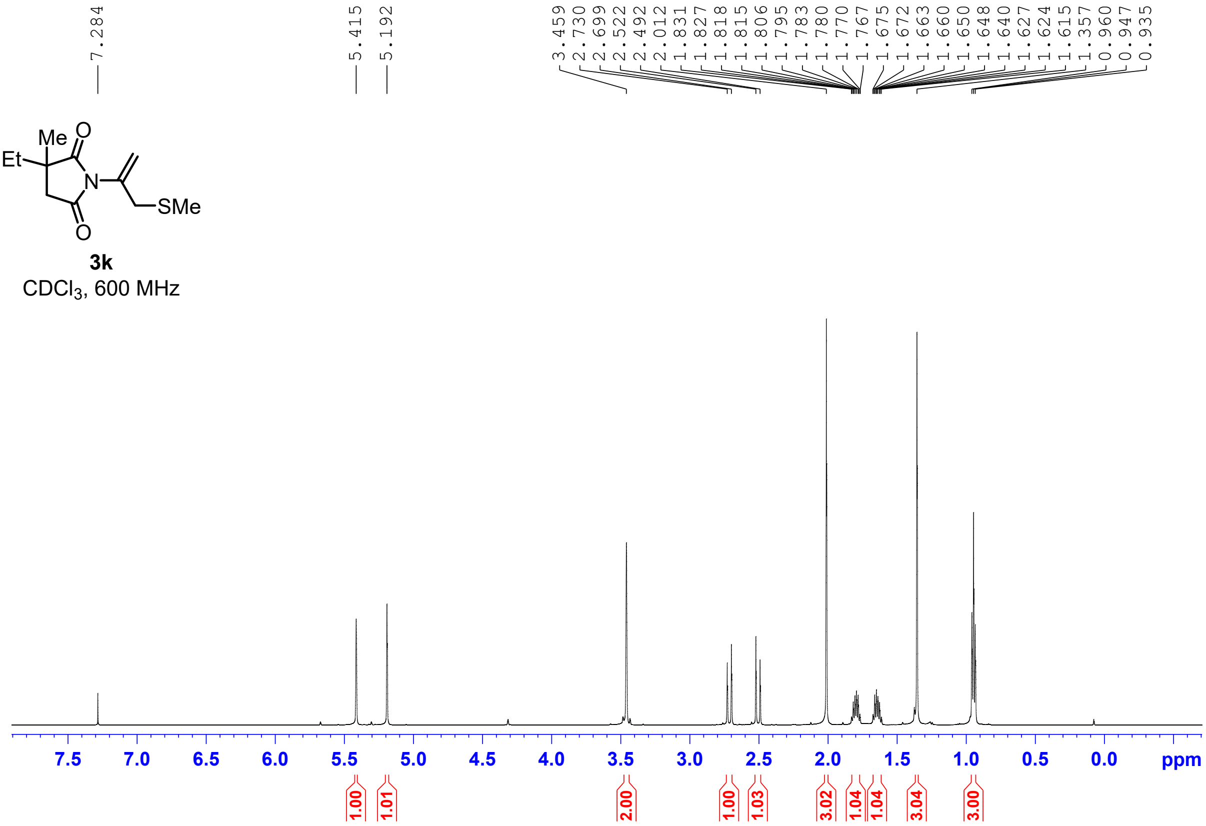

3k

$CDCl_3$, 600 MHz

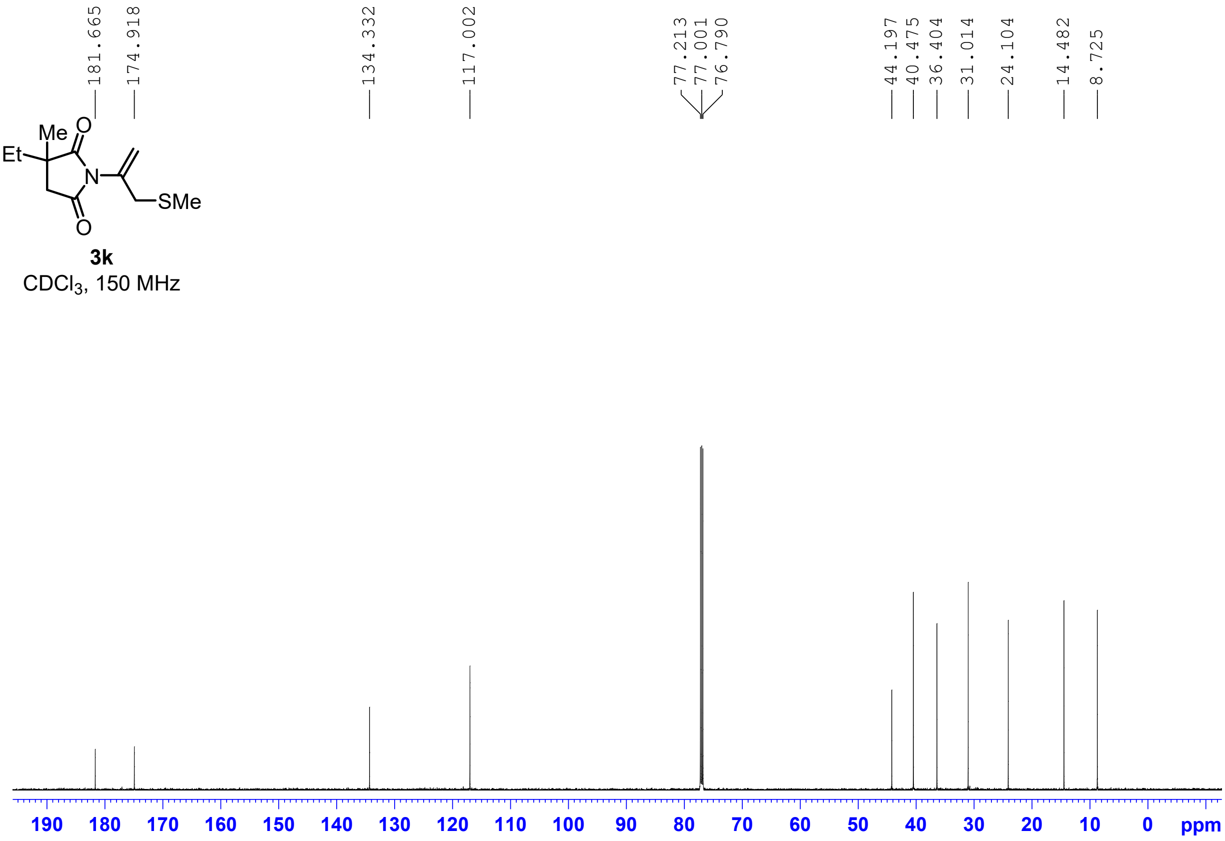

3k

$CDCl_3$, 150 MHz

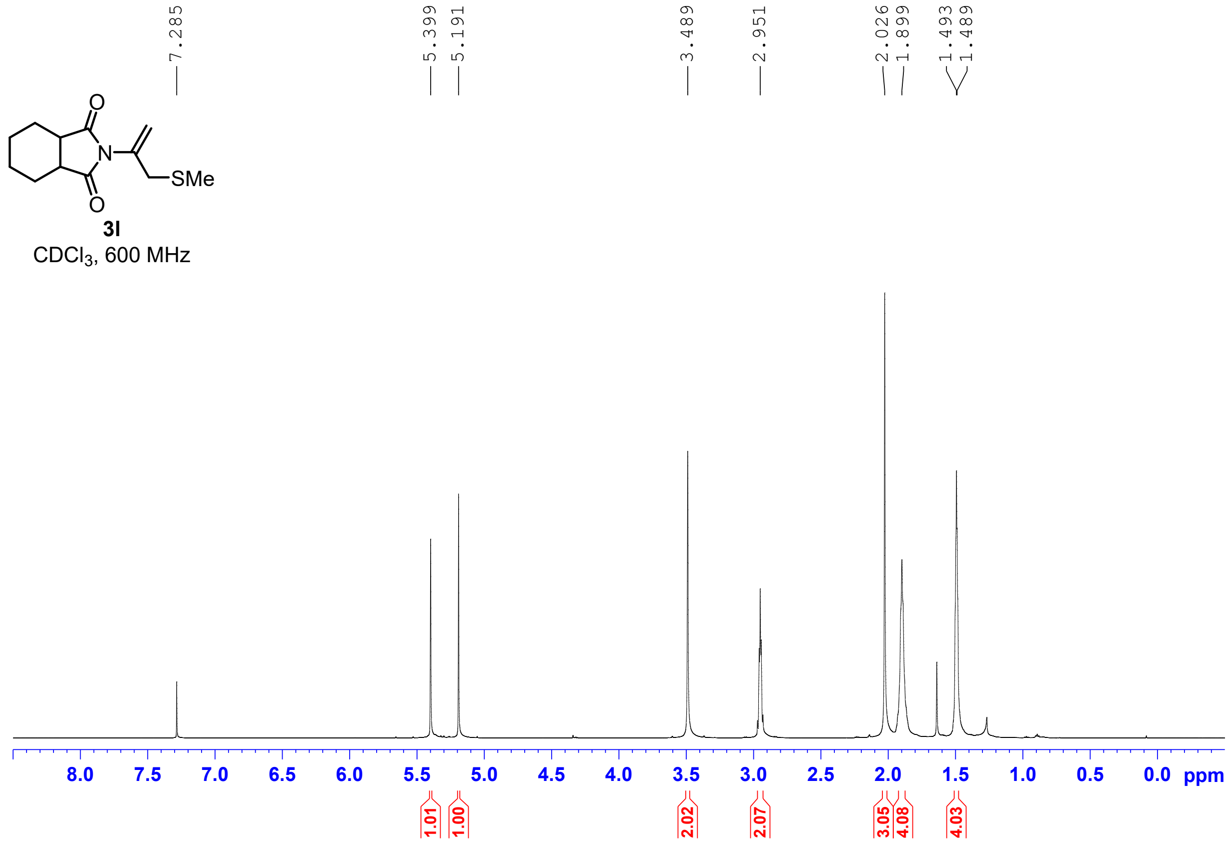
O
N
SMe
O
3l
CDCl3, 600 MHz
7.285
5.399
5.191
3.489
2.951
2.026
1.899
1.493
1.489
8.0 7.5 7.0 6.5 6.0 5.5 5.0 4.5 4.0 3.5 3.0 2.5 2.0 1.5 1.0 0.5 0.0 ppm
1.01
1.00
2.02
2.07
3.05
4.08
4.03

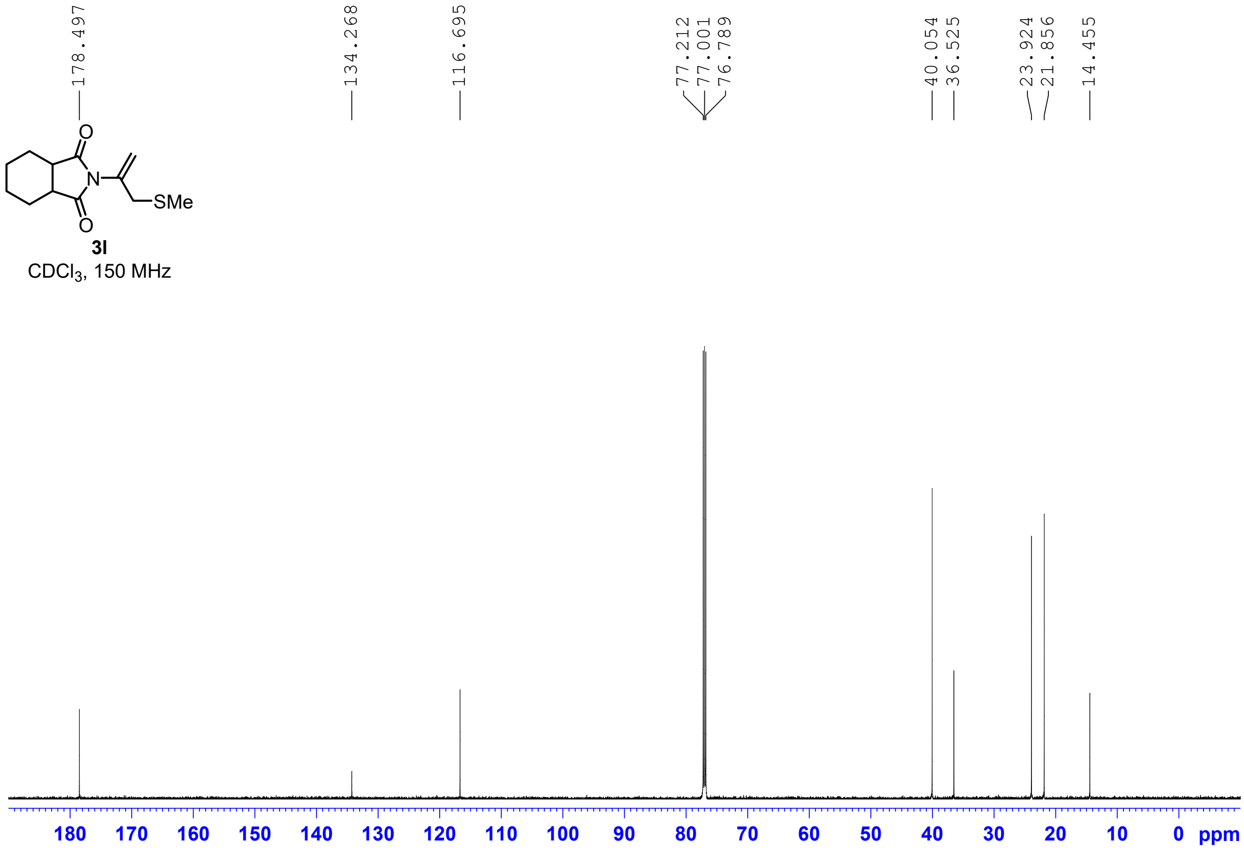
O
N
SMe
O
3l
CDCl3, 150 MHz
178.497
134.268
116.695
77.212
77.001
76.789
40.054
36.525
23.924
21.856
14.455
180 170 160 150 140 130 120 110 100 90 80 70 60 50 40 30 20 10 0 ppm

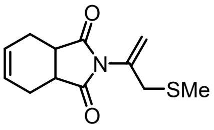

3m
$CDCl_3$, 600 MHz

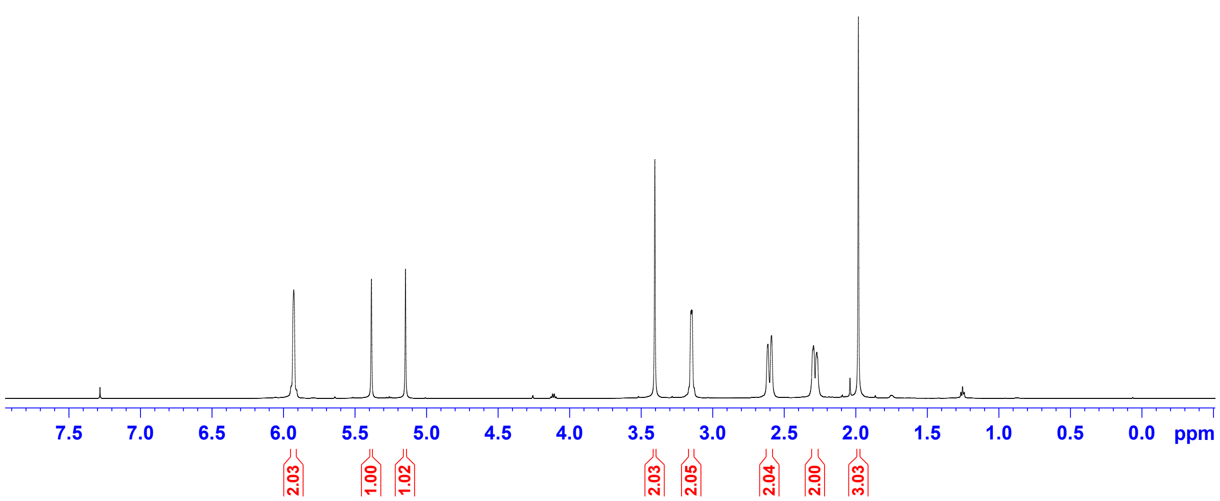

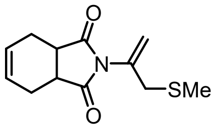

3m
$CDCl_3$, 150 MHz

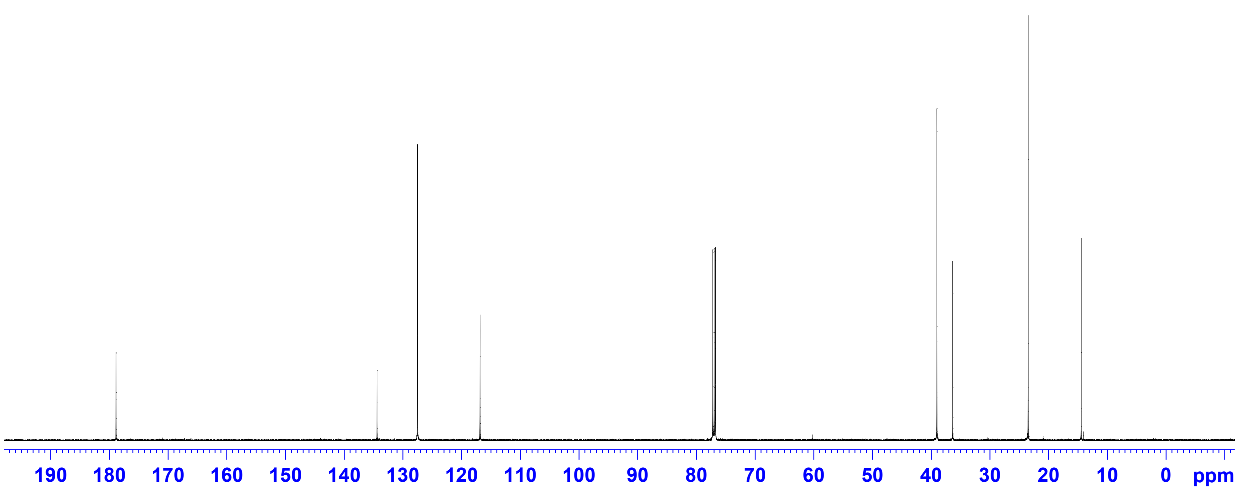

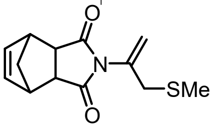

3n
$CDCl_3$, 600 MHz

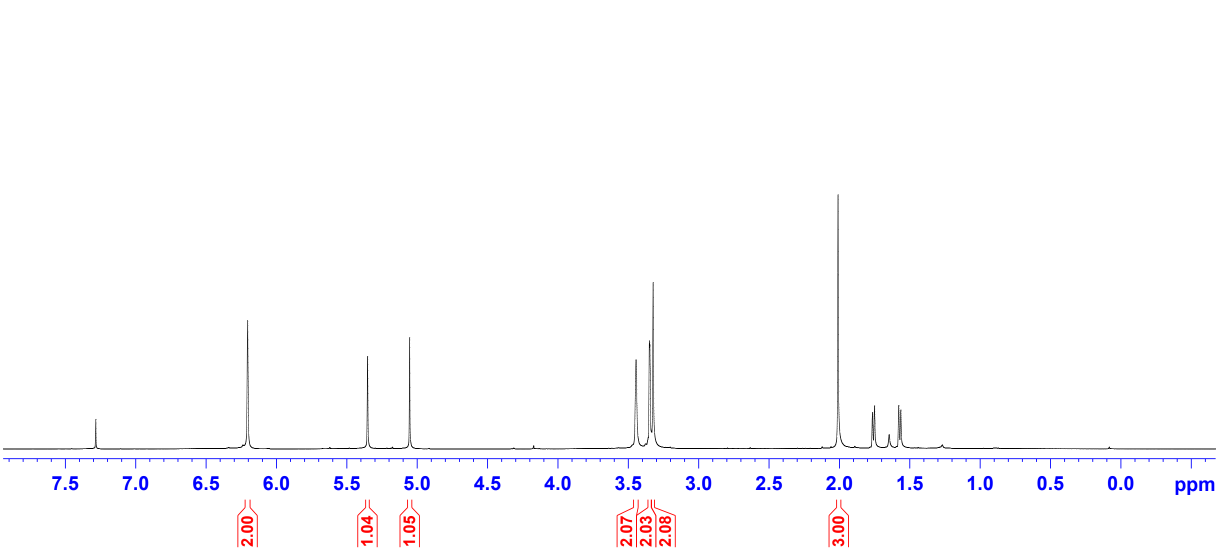

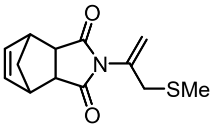

3n
$CDCl_3$, 150 MHz

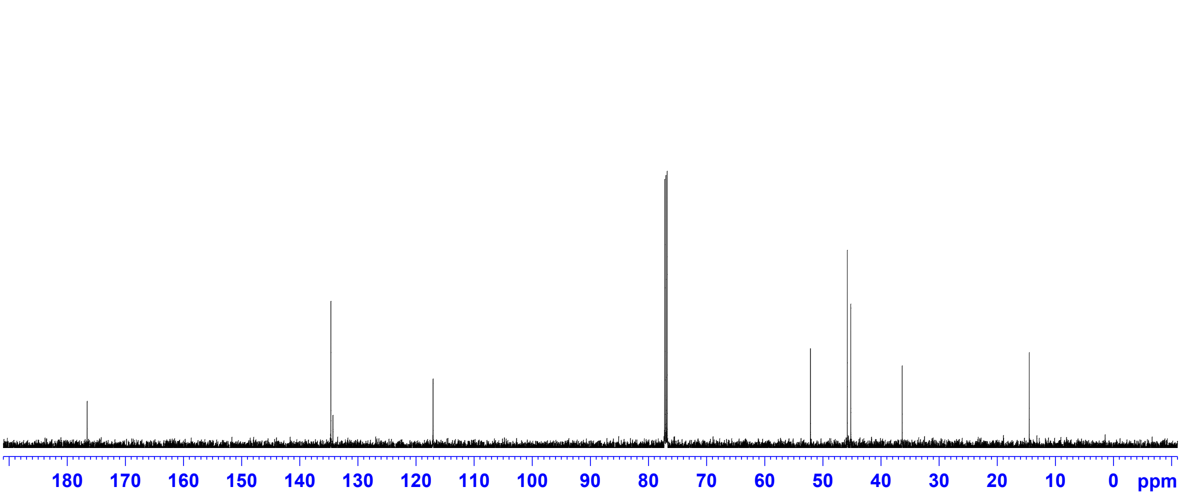

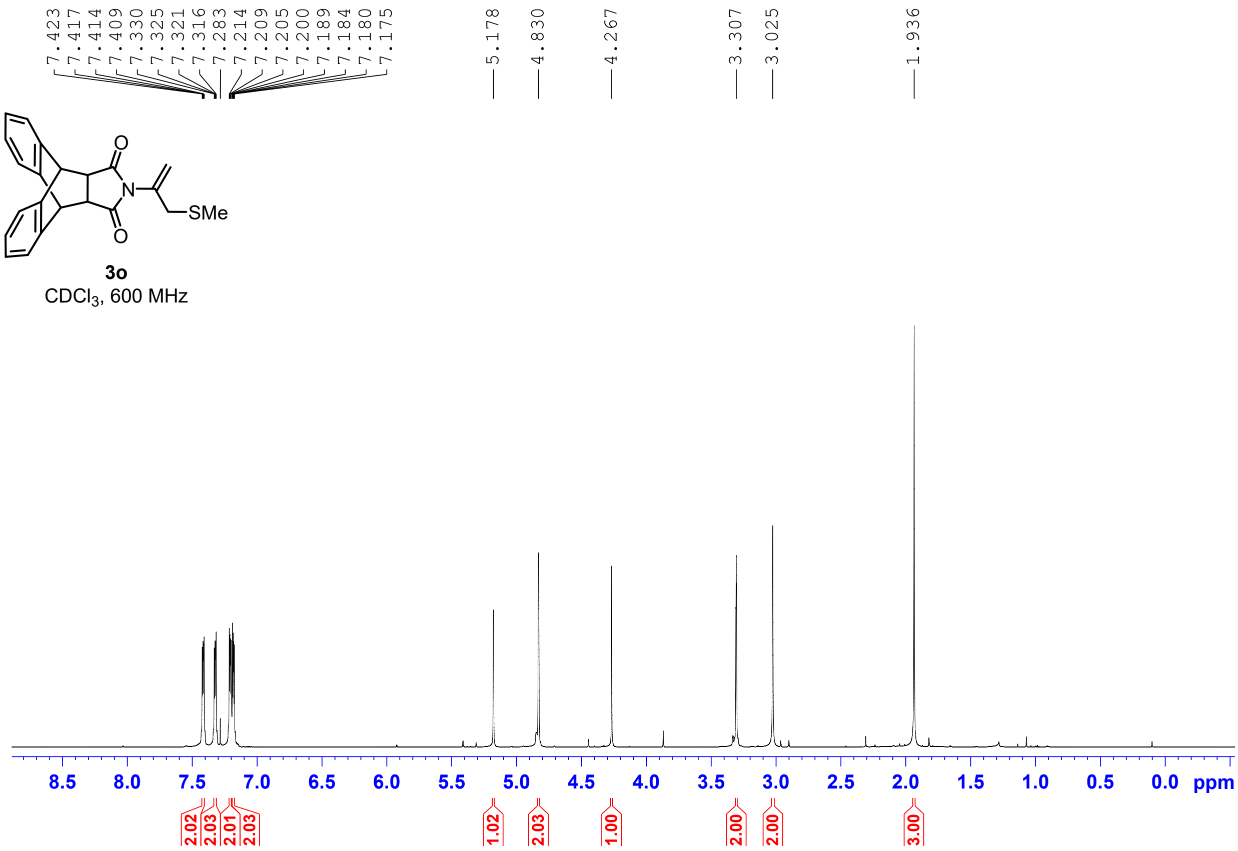
7.423
7.417
7.414
7.409
7.330
7.325
7.321
7.316
7.283
7.214
7.209
7.205
7.200
7.189
7.184
7.180
7.175
5.178
4.830
4.267
3.307
3.025
1.936
O
N
SMe
O
3o
CDCl3, 600 MHz
2.02
2.03
2.01
2.03
1.02
2.03
1.00
2.00
2.00
3.00
ppm

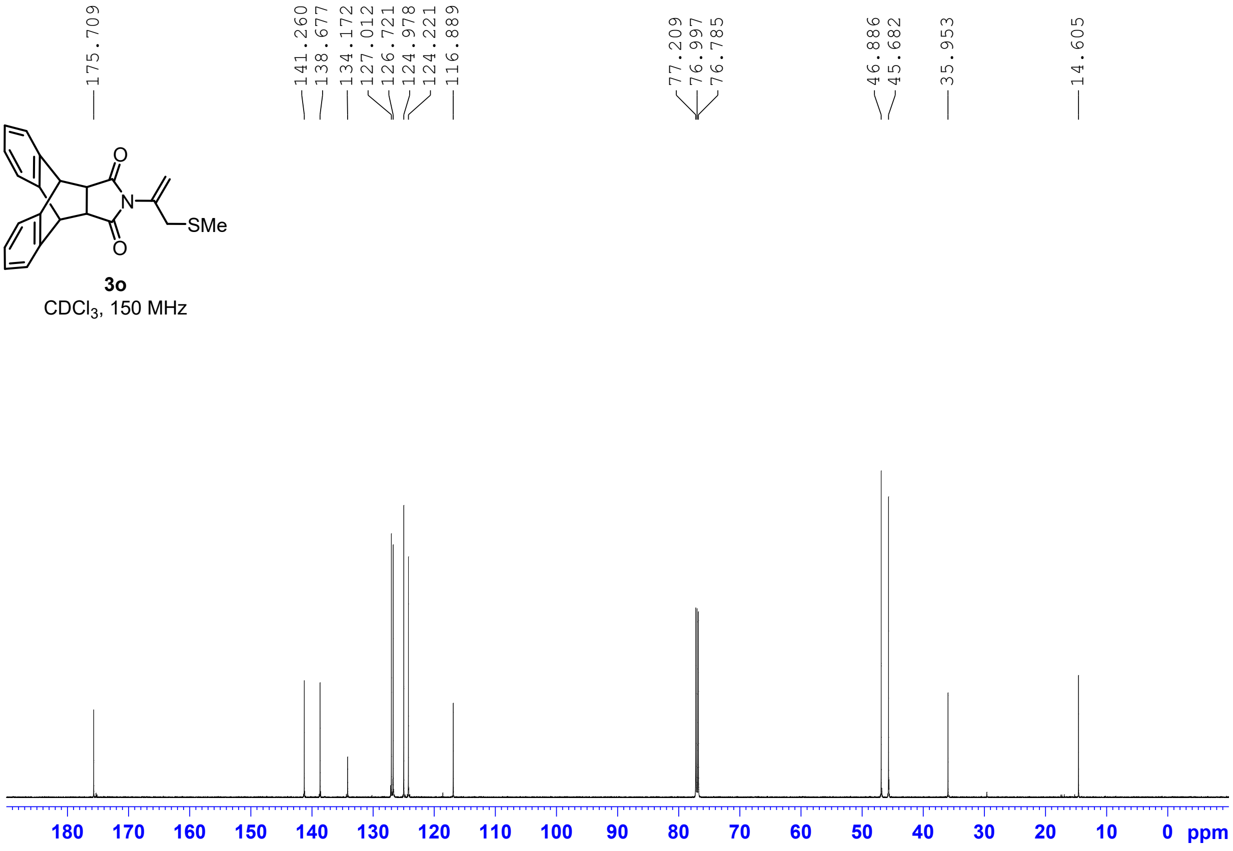
175.709
141.260
138.677
134.172
127.012
126.721
124.978
124.221
116.889
77.209
76.997
76.785
46.886
45.682
35.953
14.605
O
N
SMe
O
3o
CDCl3, 150 MHz
ppm

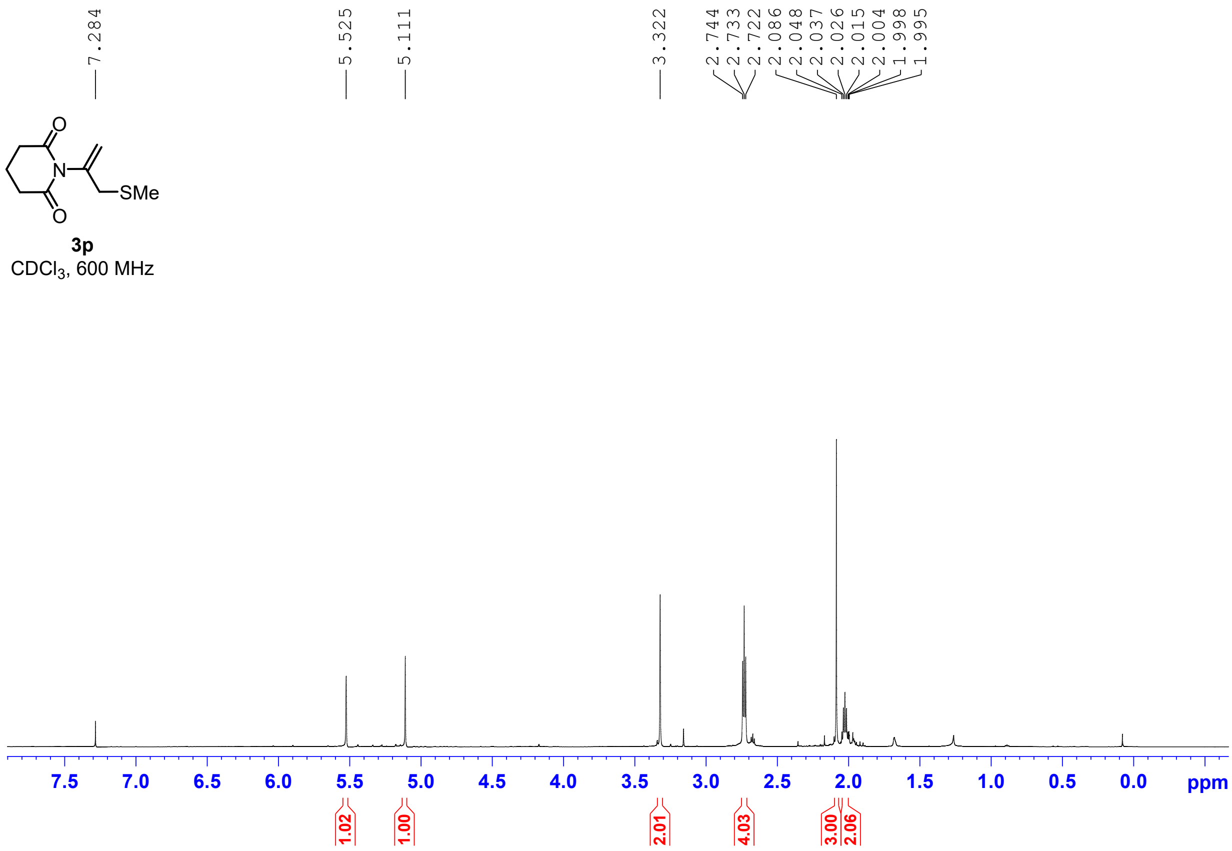
7.284
5.525
5.111
3.322
2.744
2.733
2.722
2.086
2.048
2.037
2.026
2.015
2.004
1.998
1.995
O
N
SMe
O
3p
CDCl3, 600 MHz
7.5 7.0 6.5 6.0 5.5 5.0 4.5 4.0 3.5 3.0 2.5 2.0 1.5 1.0 0.5 0.0 ppm
1.02
1.00
2.01
4.03
3.00
2.06

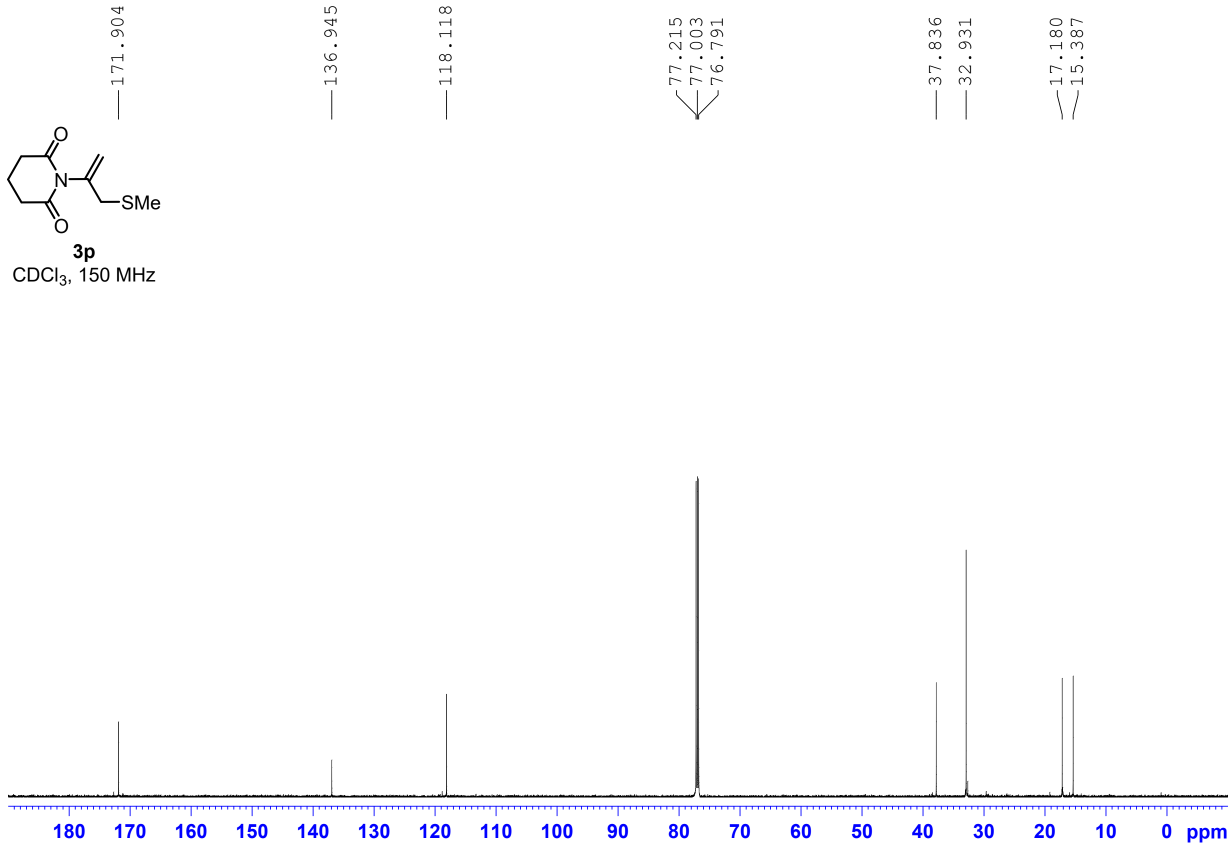
171.904
136.945
118.118
77.215
77.003
76.791
37.836
32.931
17.180
15.387
O
N
SMe
O
3p
CDCl3, 150 MHz
180 170 160 150 140 130 120 110 100 90 80 70 60 50 40 30 20 10 0 ppm

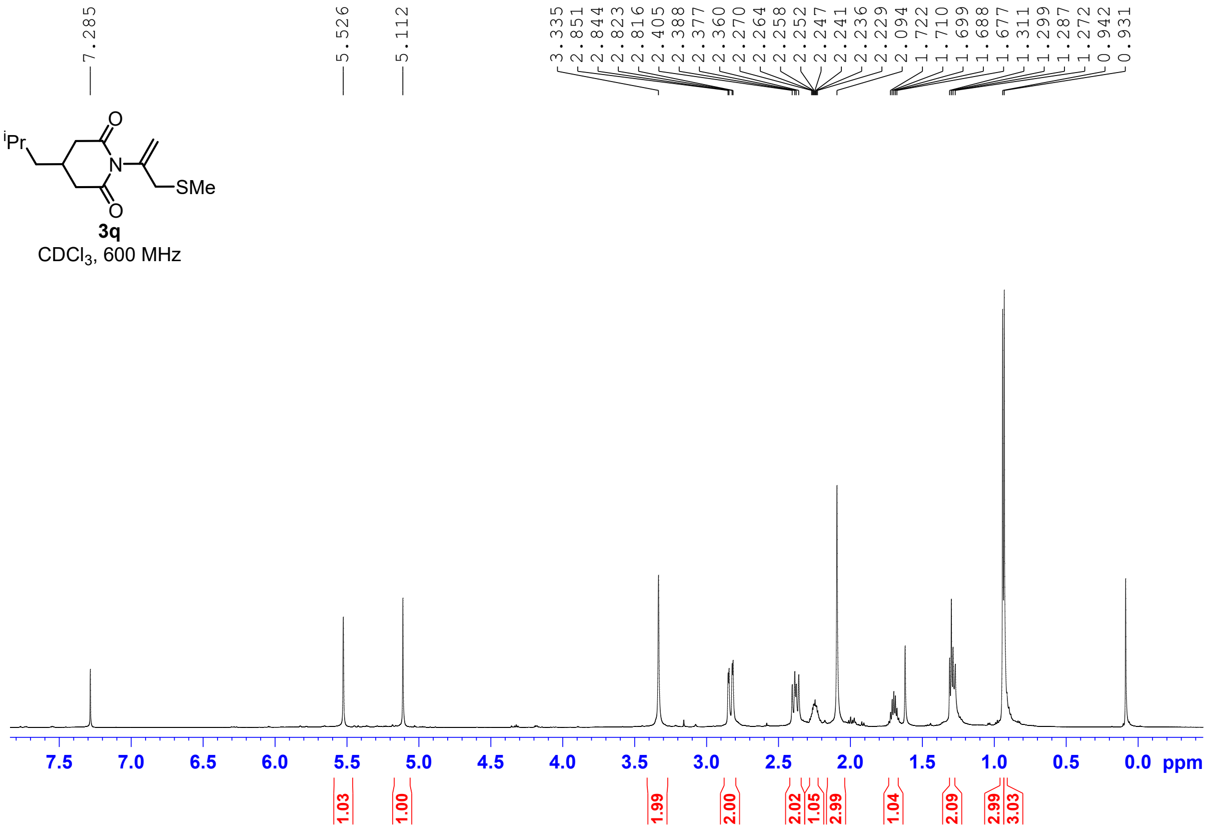
7.285
5.526
5.112
3.335
2.851
2.844
2.823
2.816
2.405
2.388
2.377
2.360
2.270
2.264
2.258
2.252
2.247
2.241
2.236
2.229
2.094
1.722
1.710
1.699
1.688
1.677
1.311
1.299
1.287
1.272
0.942
0.931
iPr
N
O
O
SMe
3q
CDCl3, 600 MHz
7.5
7.0
6.5
6.0
5.5
5.0
4.5
4.0
3.5
3.0
2.5
2.0
1.5
1.0
0.5
0.0 ppm
1.03
1.00
1.99
2.00
2.02
1.05
2.99
1.04
2.09
2.99
3.03

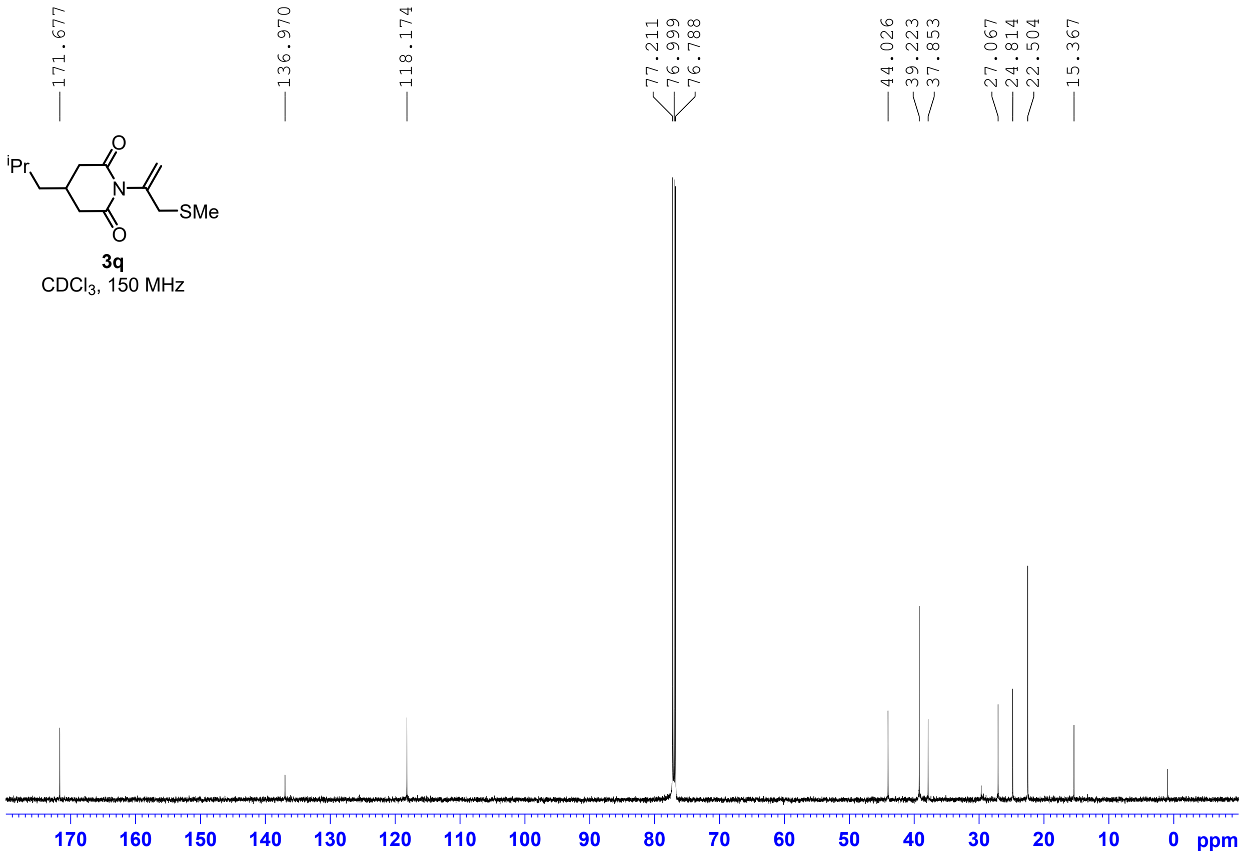
171.677
136.970
118.174
77.211
76.999
76.788
44.026
39.223
37.853
27.067
24.814
22.504
15.367
iPr
N
O
O
SMe
3q
CDCl3, 150 MHz
170
160
150
140
130
120
110
100
90
80
70
60
50
40
30
20
10
0 ppm

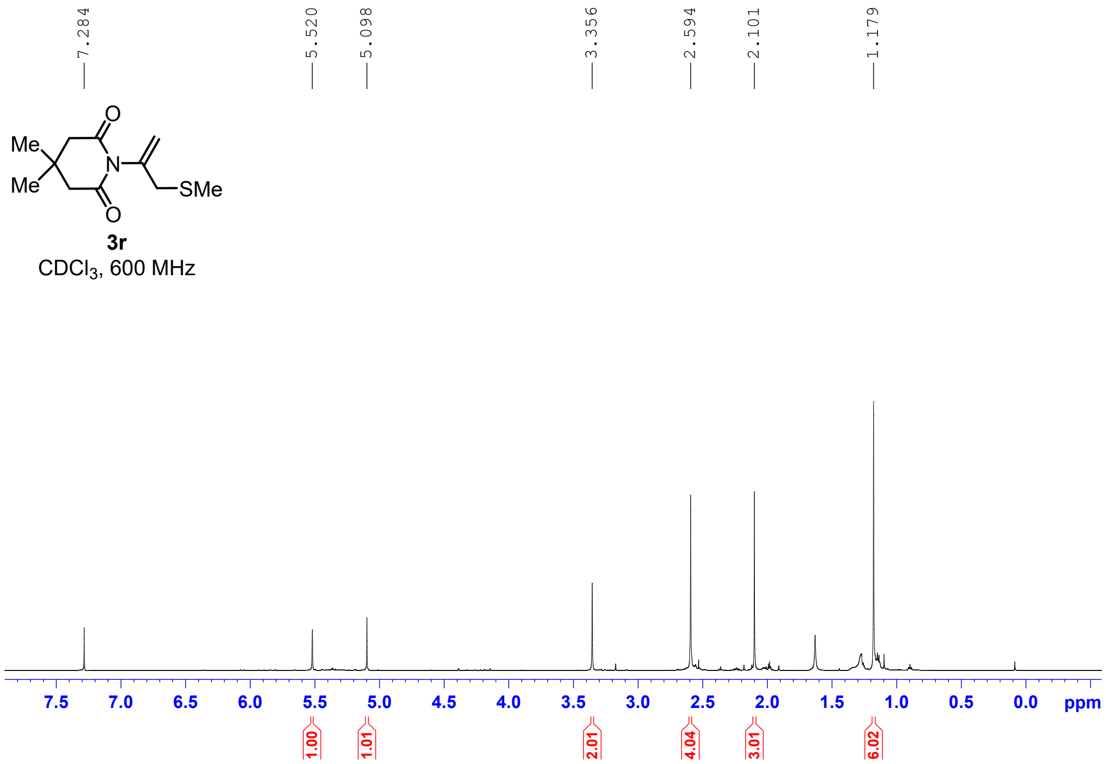
7.284
5.520
5.098
3.356
2.594
2.101
1.179
Me
Me
O
O
N
SMe
3r
CDCl$_3$, 600 MHz
7.5 7.0 6.5 6.0 5.5 5.0 4.5 4.0 3.5 3.0 2.5 2.0 1.5 1.0 0.5 0.0 ppm
1.00
1.01
2.01
4.04
3.01
6.02

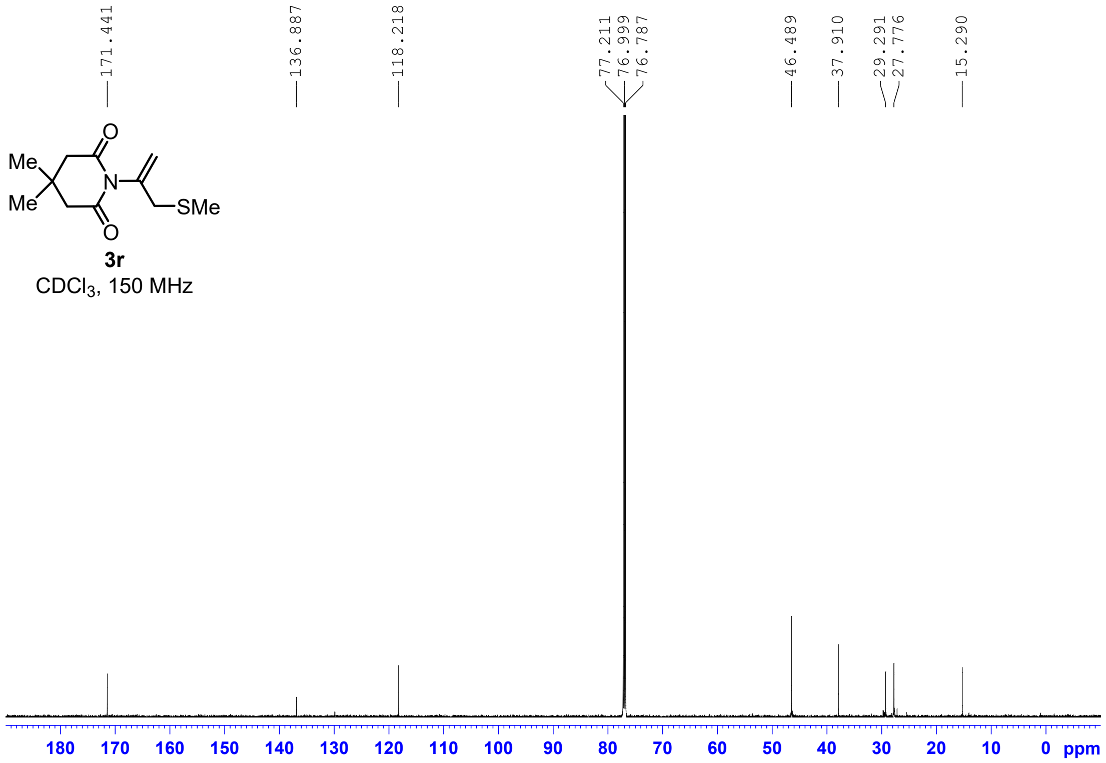
171.441
136.887
118.218
77.211
76.999
76.787
46.489
37.910
29.291
27.776
15.290
Me
Me
O
O
N
SMe
3r
CDCl$_3$, 150 MHz
180 170 160 150 140 130 120 110 100 90 80 70 60 50 40 30 20 10 0 ppm

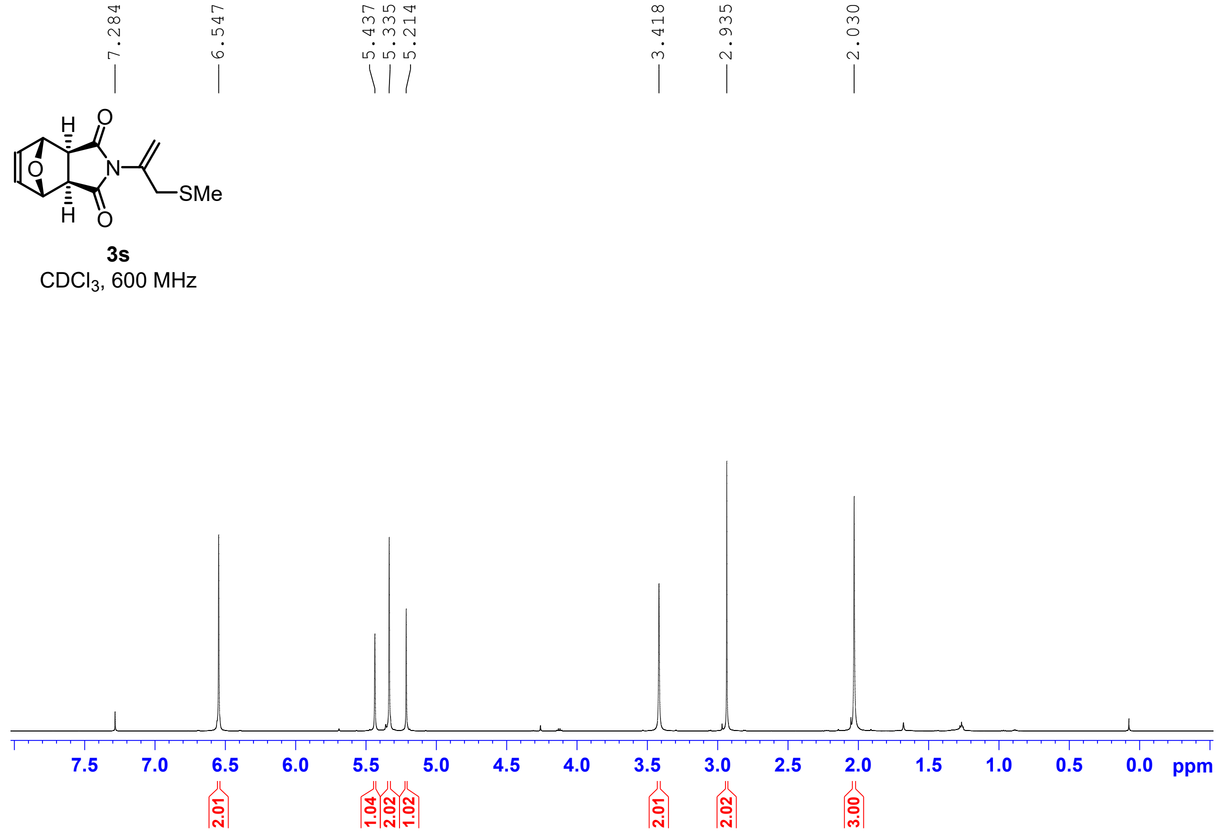

3s

$CDCl_3$, 600 MHz

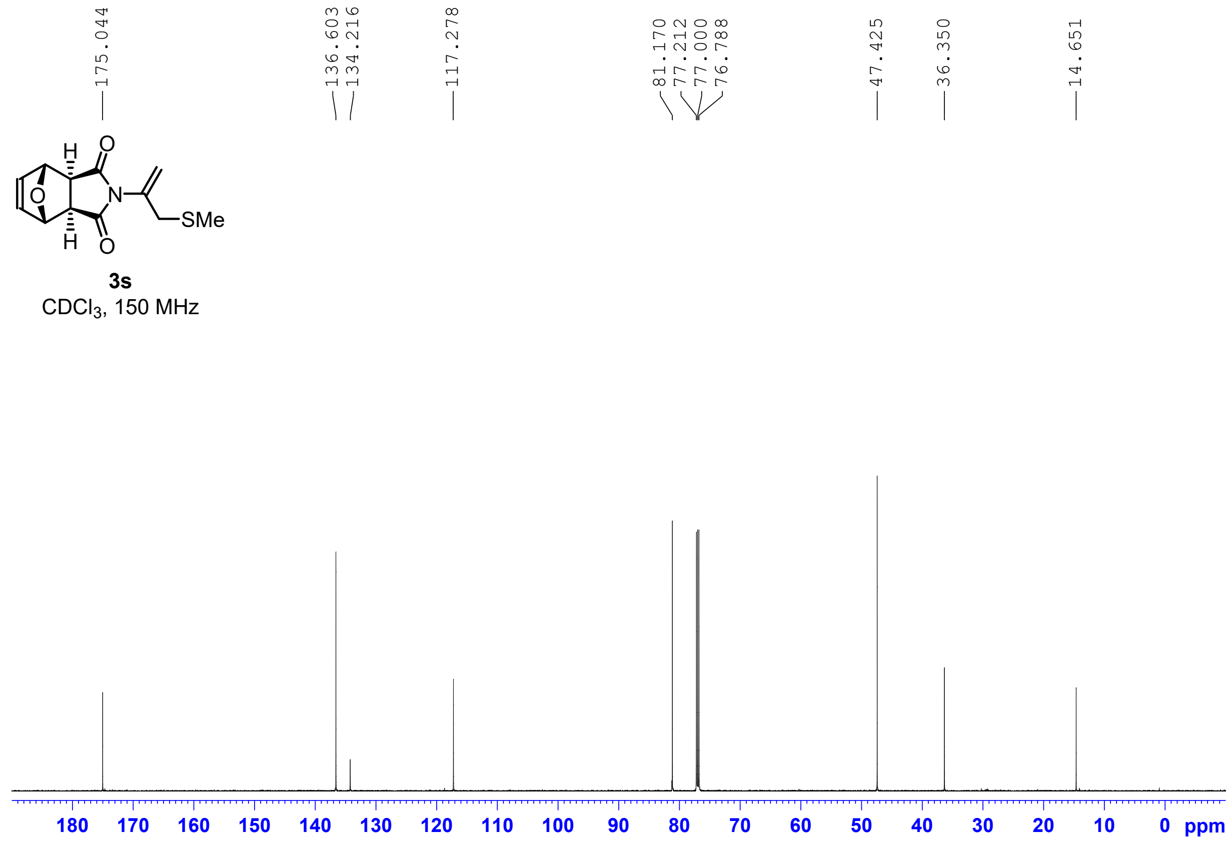

3s

$CDCl_3$, 150 MHz

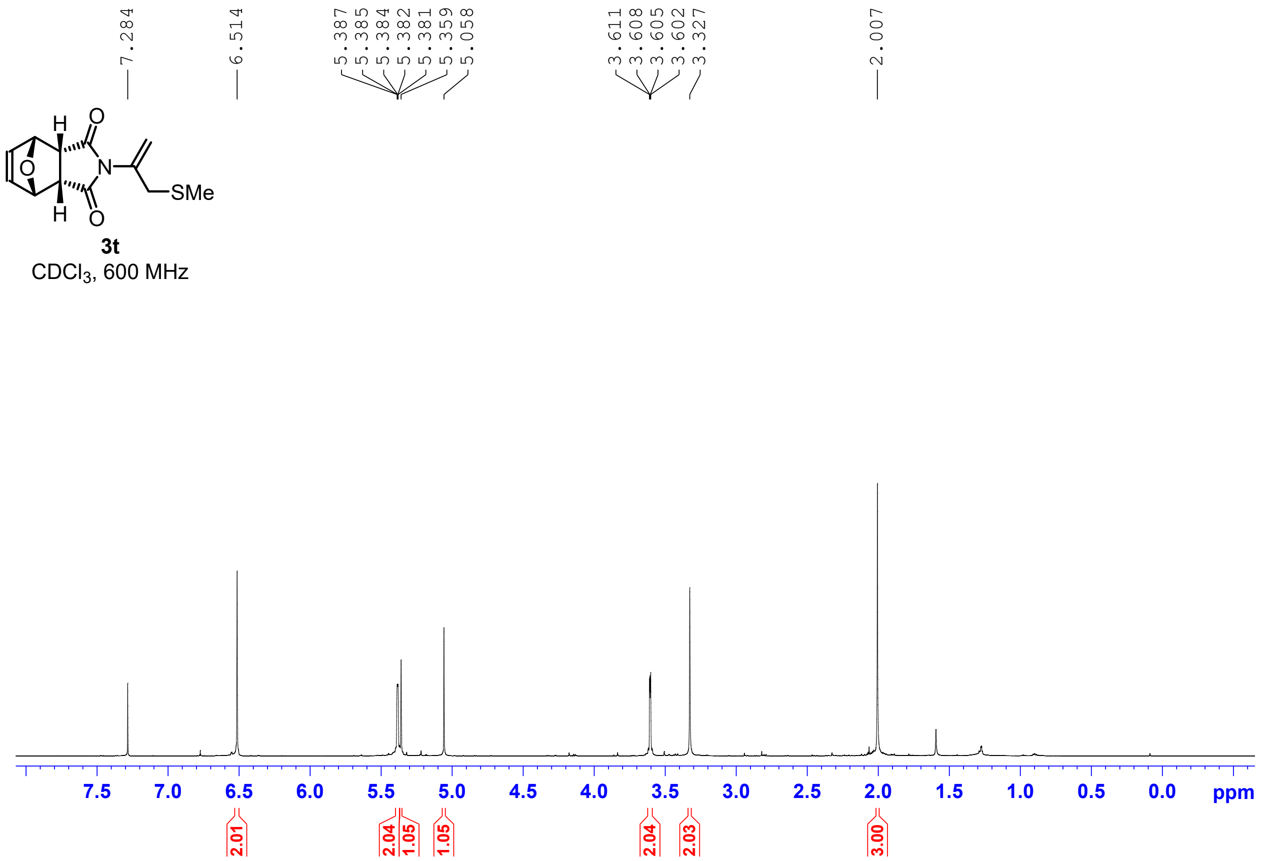
7.284
6.514
5.387
5.385
5.384
5.382
5.381
5.359
5.058
3.611
3.608
3.605
3.602
3.327
2.007
H
O
O
N
SMe
H
O
3t
CDCl3, 600 MHz
7.5
7.0
6.5
6.0
5.5
5.0
4.5
4.0
3.5
3.0
2.5
2.0
1.5
1.0
0.5
0.0
ppm
2.01
2.04
1.05
1.05
2.04
2.03
3.00

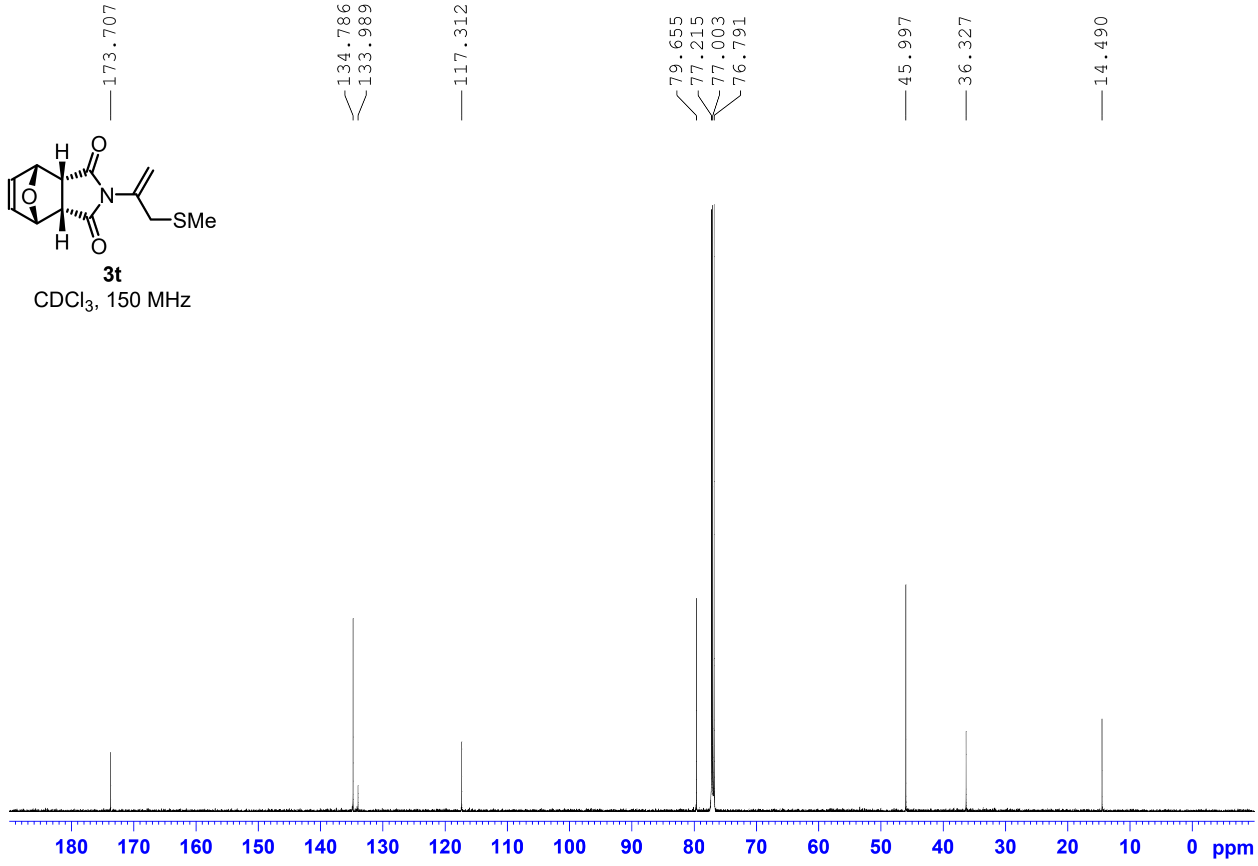
173.707
134.786
133.989
117.312
79.655
77.215
77.003
76.791
45.997
36.327
14.490
H
O
O
N
SMe
H
O
3t
CDCl3, 150 MHz
180
170
160
150
140
130
120
110
100
90
80
70
60
50
40
30
20
10
0
ppm

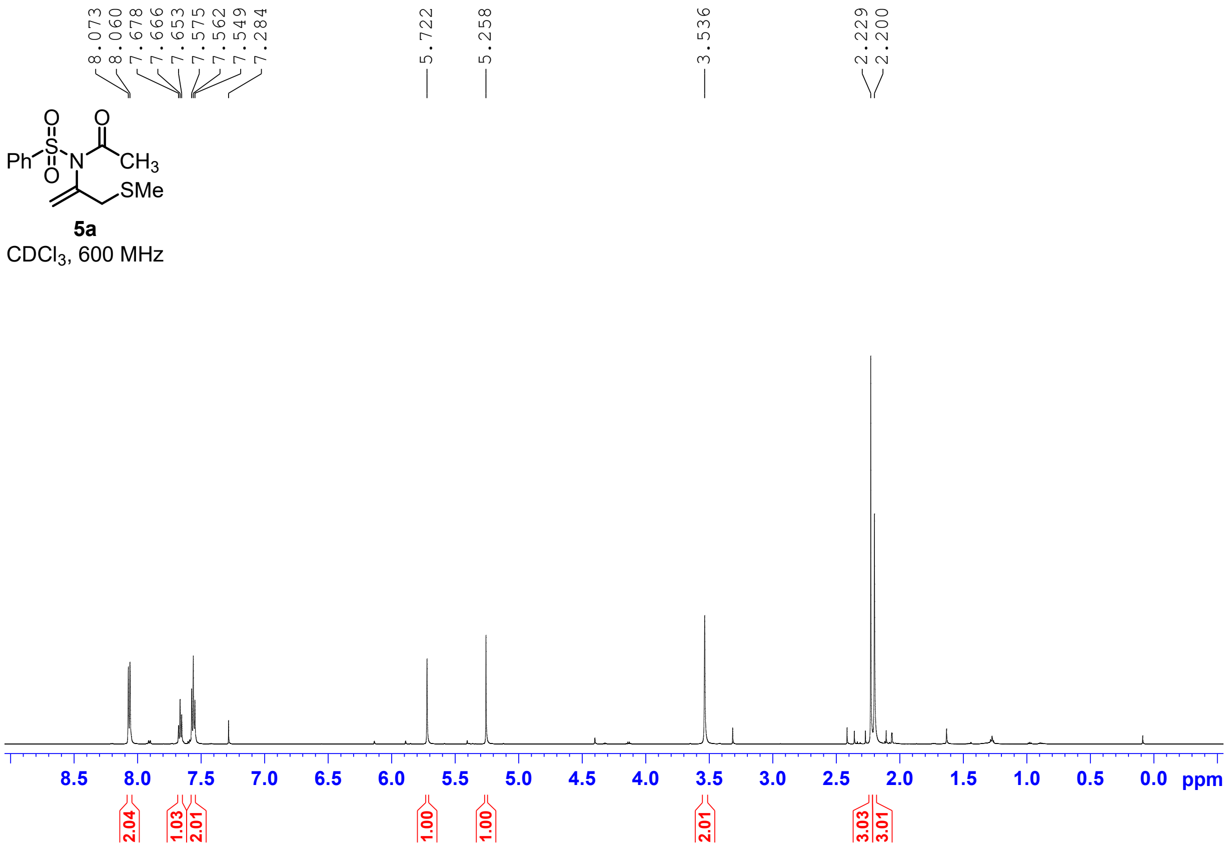
8.073
8.060
7.678
7.666
7.653
7.575
7.562
7.549
7.284
5.722
5.258
3.536
2.229
2.200
Ph
O
O
S
N
O
CH3
SMe
5a
CDCl3, 600 MHz
8.5
8.0
7.5
7.0
6.5
6.0
5.5
5.0
4.5
4.0
3.5
3.0
2.5
2.0
1.5
1.0
0.5
0.0
ppm
2.04
1.03
2.01
1.00
1.00
2.01
3.03
3.01

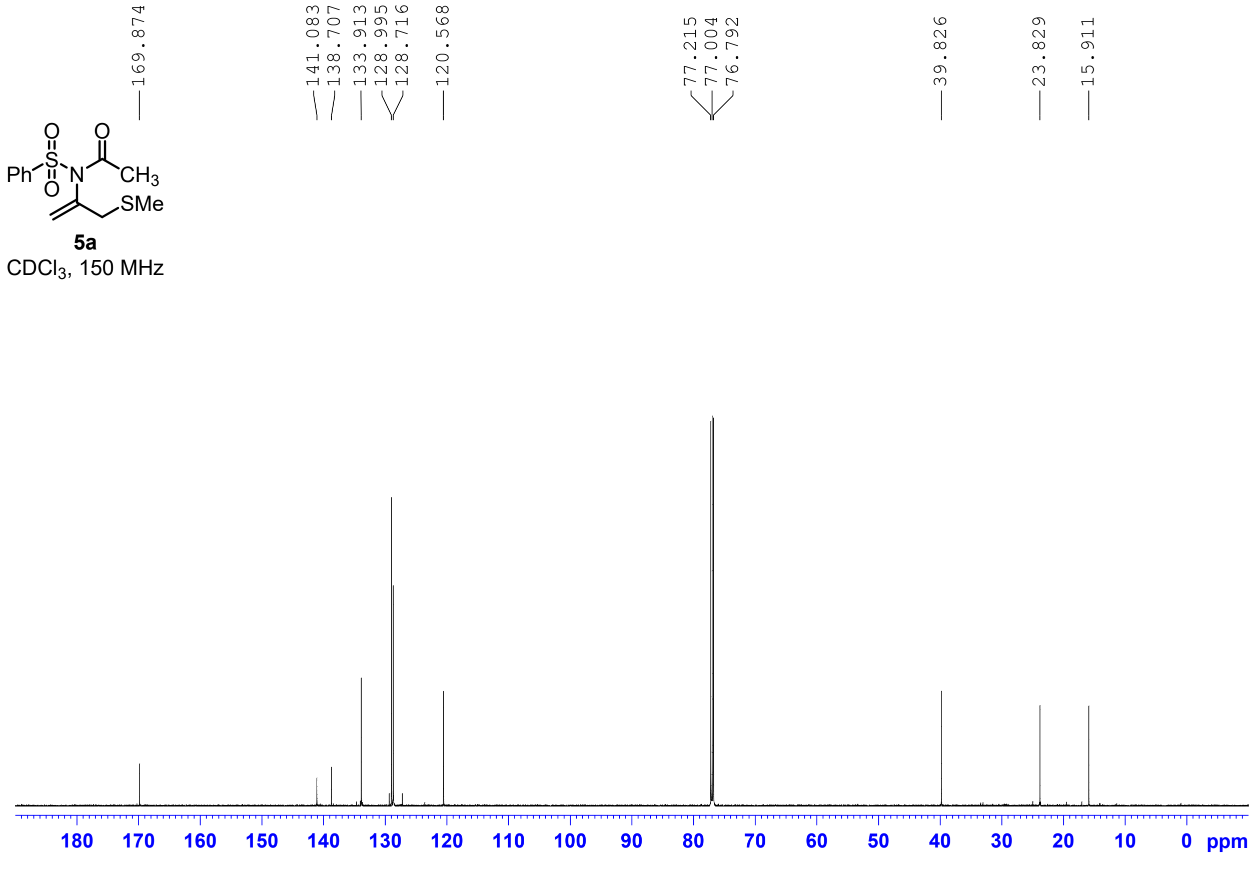
169.874
141.083
138.707
133.913
128.995
128.716
120.568
77.215
77.004
76.792
39.826
23.829
15.911
Ph
O
O
S
N
O
CH3
SMe
5a
CDCl3, 150 MHz
180
170
160
150
140
130
120
110
100
90
80
70
60
50
40
30
20
10
0
ppm

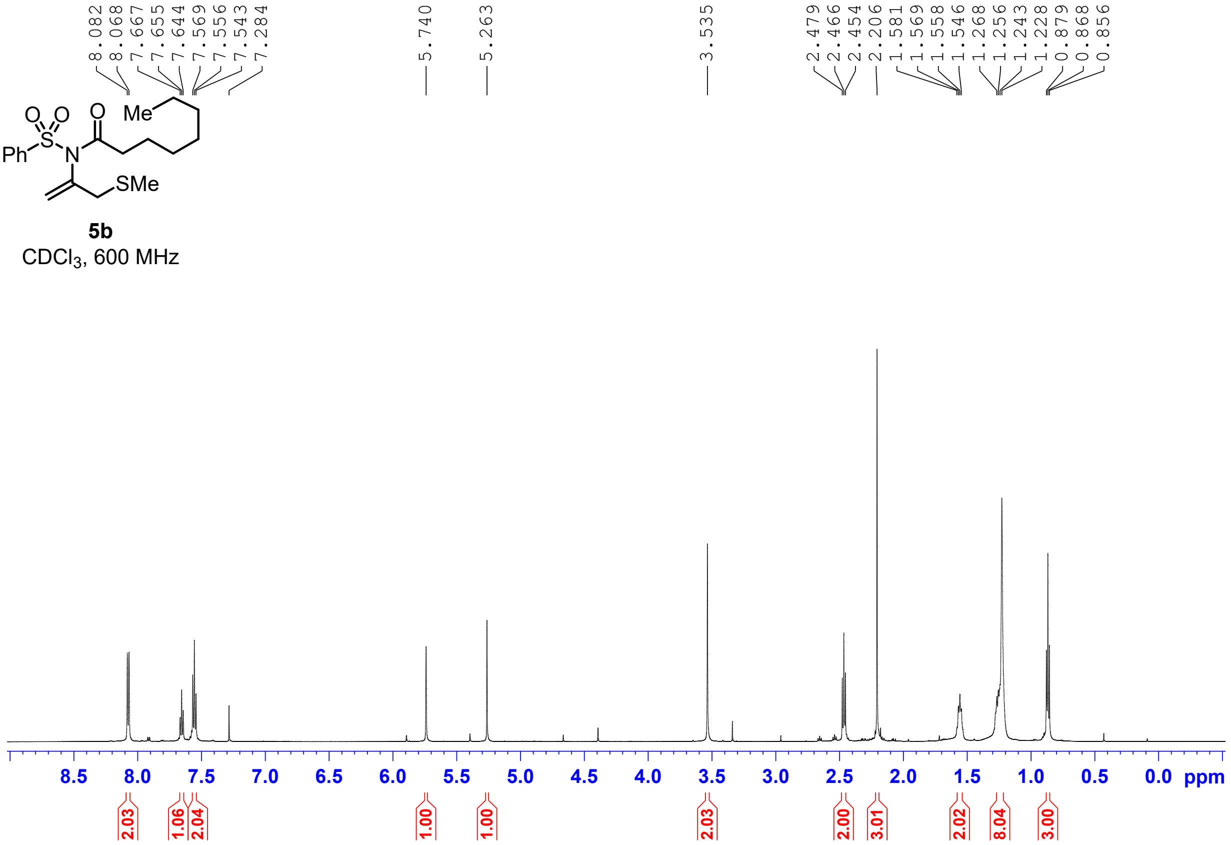
8.082
8.068
7.667
7.655
7.644
7.569
7.556
7.543
7.284
5.740
5.263
3.535
2.479
2.466
2.454
2.206
1.581
1.569
1.558
1.546
1.268
1.256
1.243
1.228
0.879
0.868
0.856
Me
Ph
S
N
O
SMe
5b
CDCl3, 600 MHz
2.03
1.06
2.04
1.00
1.00
2.03
2.00
3.01
2.02
8.04
3.00
ppm

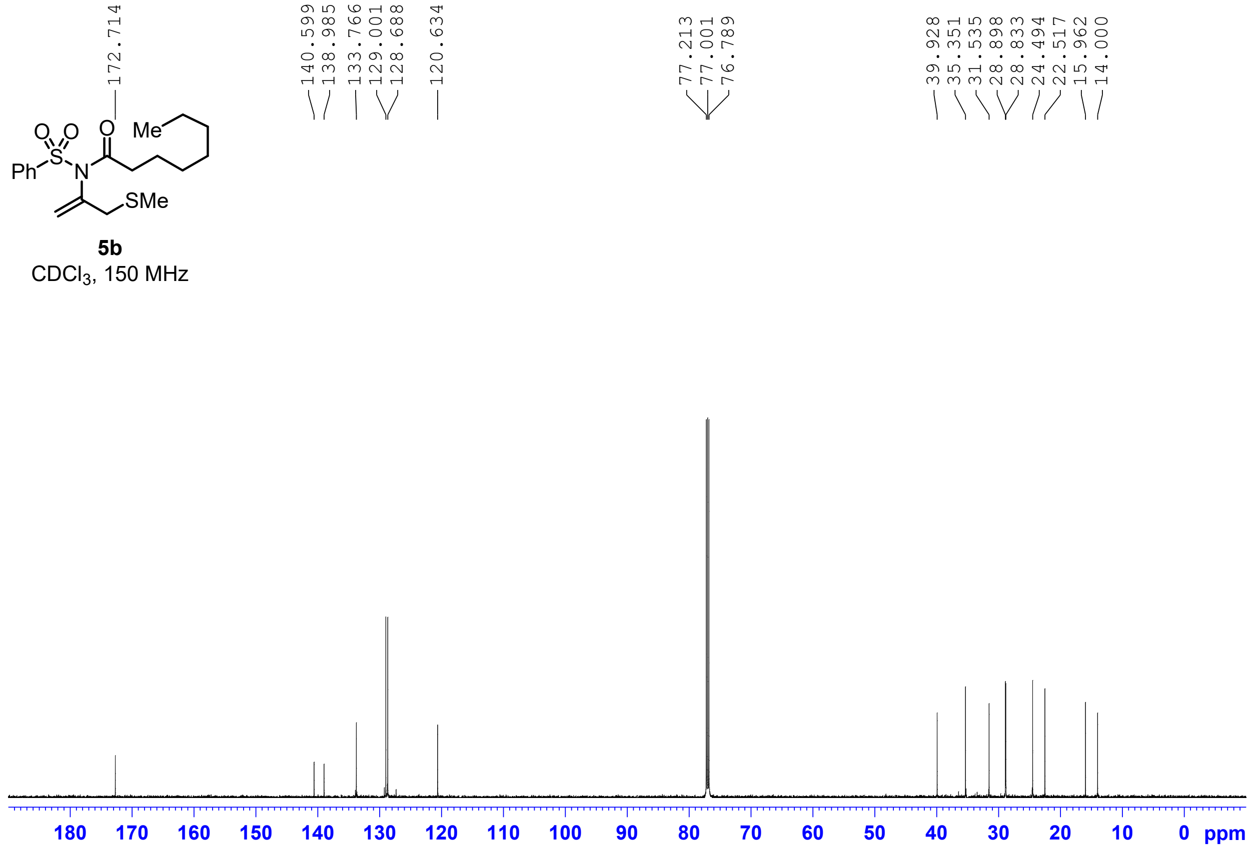
172.714
140.599
138.985
133.766
129.001
128.688
120.634
77.213
77.001
76.789
39.928
35.351
31.535
28.898
28.833
24.494
22.517
15.962
14.000
Me
Ph
S
N
O
SMe
5b
CDCl3, 150 MHz
ppm

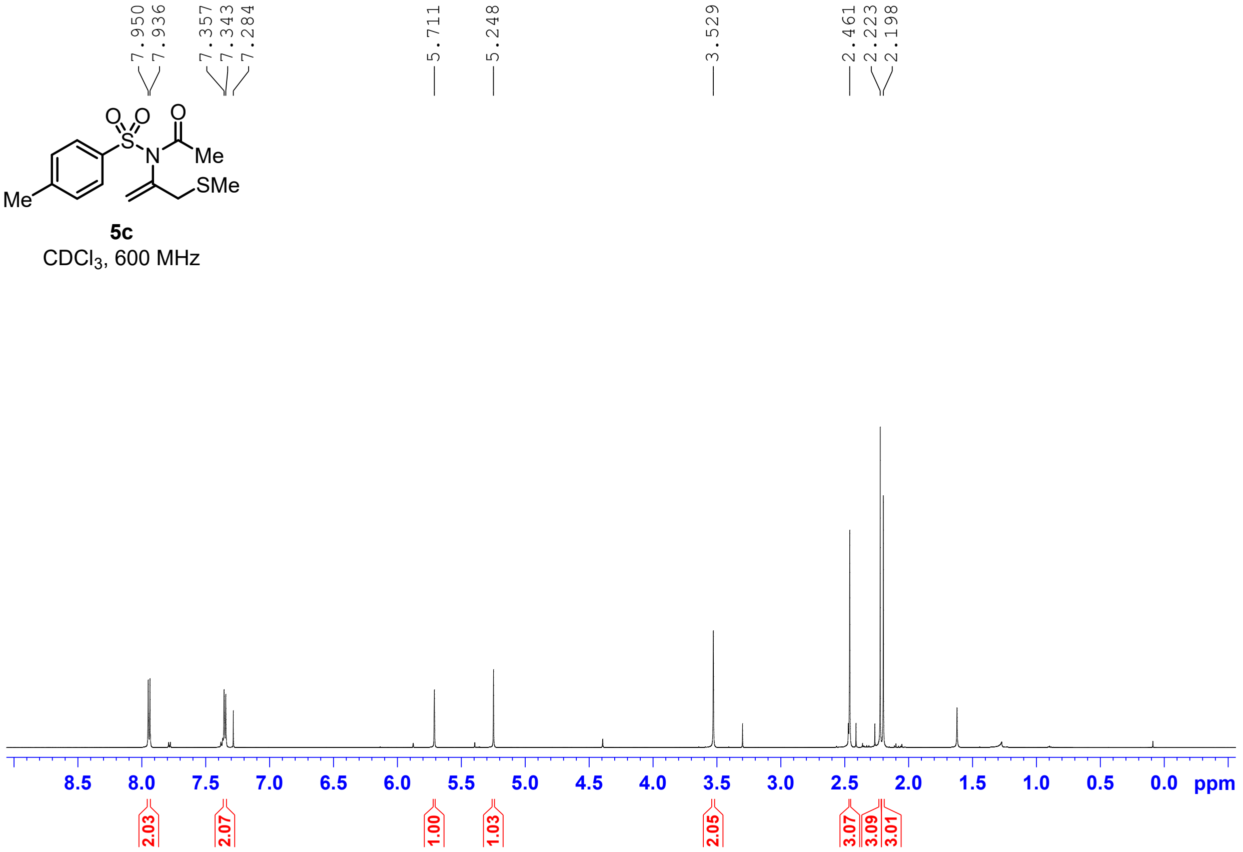
7.950
7.936
7.357
7.343
7.284
5.711
5.248
3.529
2.461
2.223
2.198
Me
SMe
5c
CDCl$_3$, 600 MHz
2.03
2.07
1.00
1.03
2.05
3.07
3.09
3.01
ppm

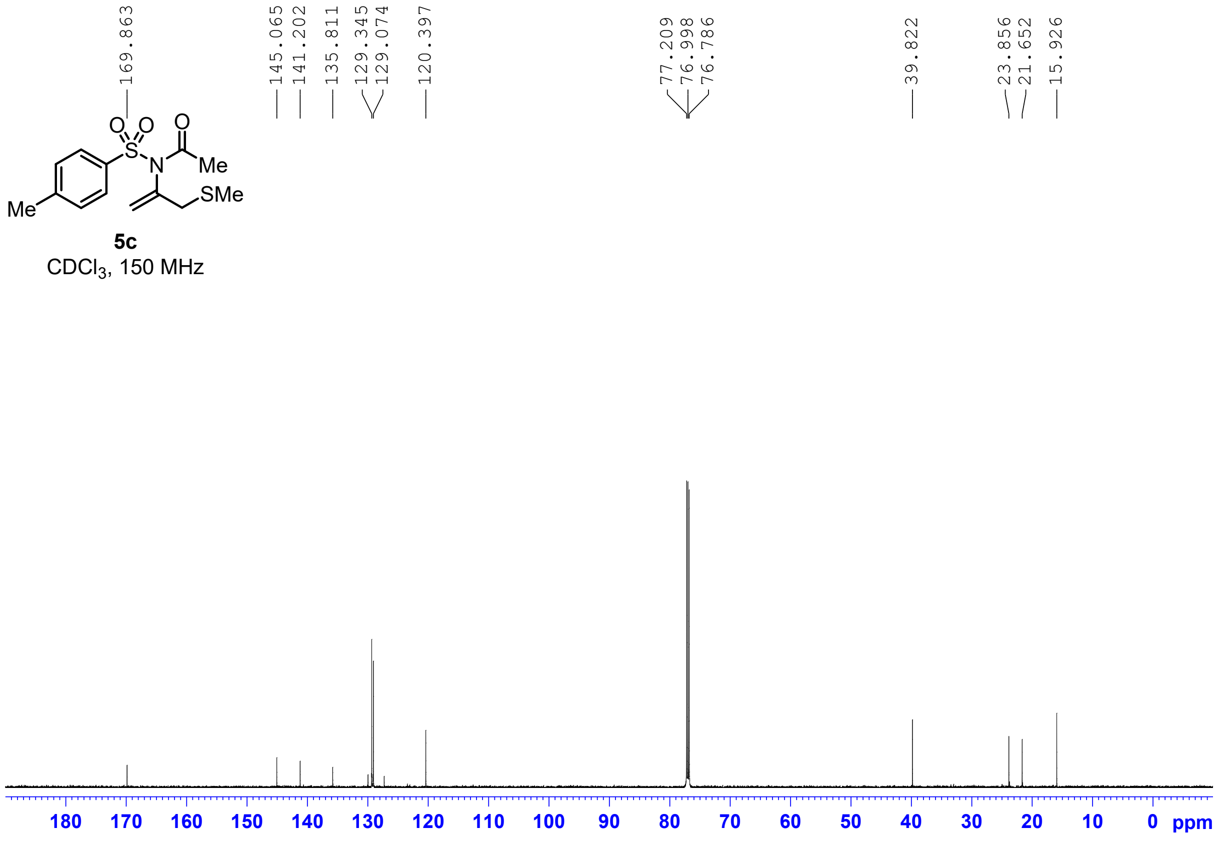
169.863
145.065
141.202
135.811
129.345
129.074
120.397
77.209
76.998
76.786
39.822
23.856
21.652
15.926
Me
SMe
5c
CDCl$_3$, 150 MHz
ppm

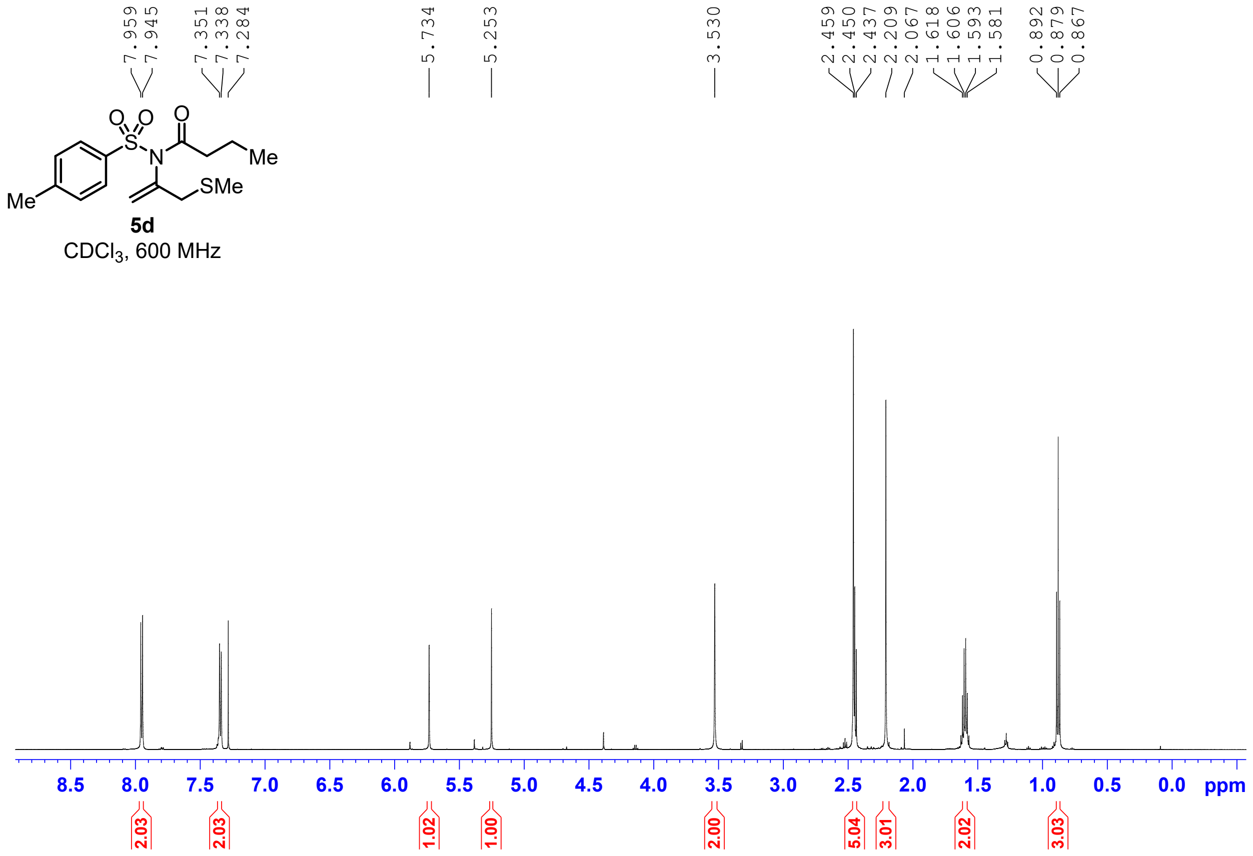
7.959
7.945
7.351
7.338
7.284
5.734
5.253
3.530
2.459
2.450
2.437
2.209
2.067
1.618
1.606
1.593
1.581
0.892
0.879
0.867
Me
SMe
5d
CDCl3, 600 MHz
8.5 8.0 7.5 7.0 6.5 6.0 5.5 5.0 4.5 4.0 3.5 3.0 2.5 2.0 1.5 1.0 0.5 0.0 ppm
2.03
2.03
1.02
1.00
2.00
5.04
3.01
2.02
3.03

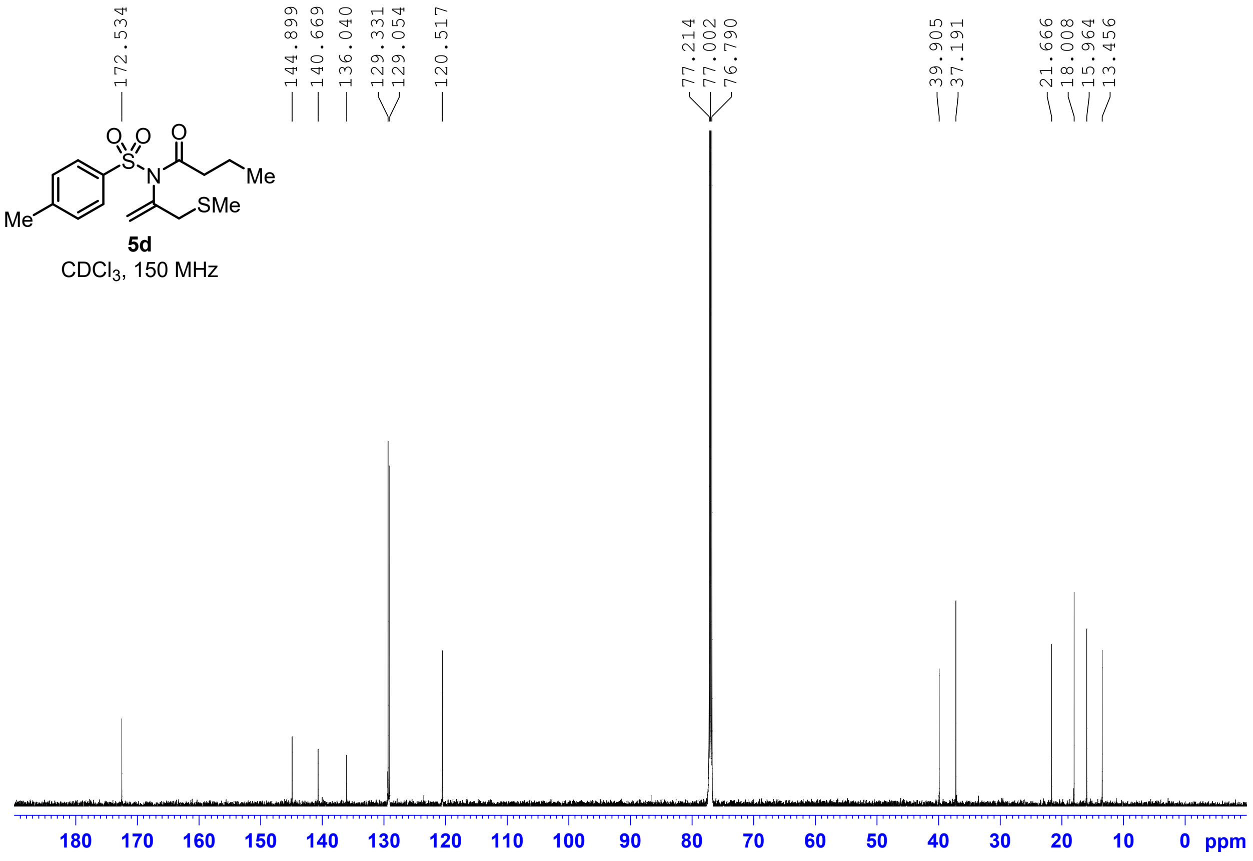
172.534
144.899
140.669
136.040
129.331
129.054
120.517
77.214
77.002
76.790
39.905
37.191
21.666
18.008
15.964
13.456
Me
SMe
5d
CDCl3, 150 MHz
180 170 160 150 140 130 120 110 100 90 80 70 60 50 40 30 20 10 0 ppm

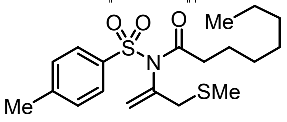

5e
$CDCl_3$, 600 MHz

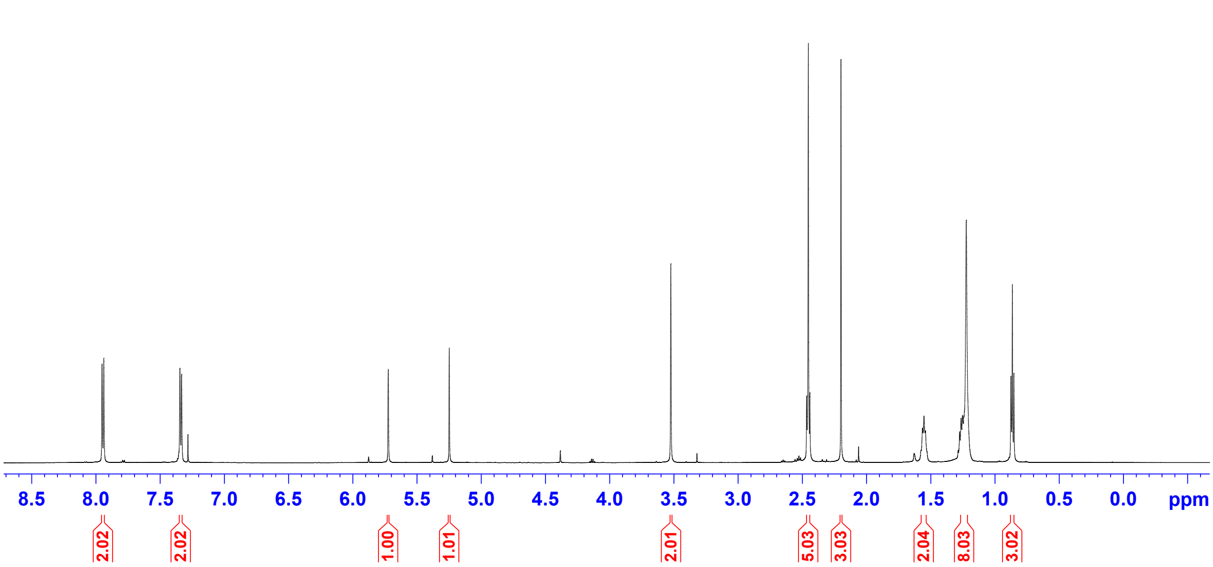

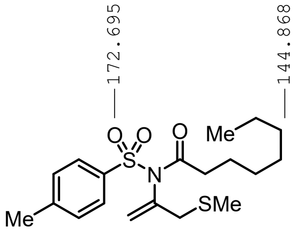

5e
$CDCl_3$, 150 MHz

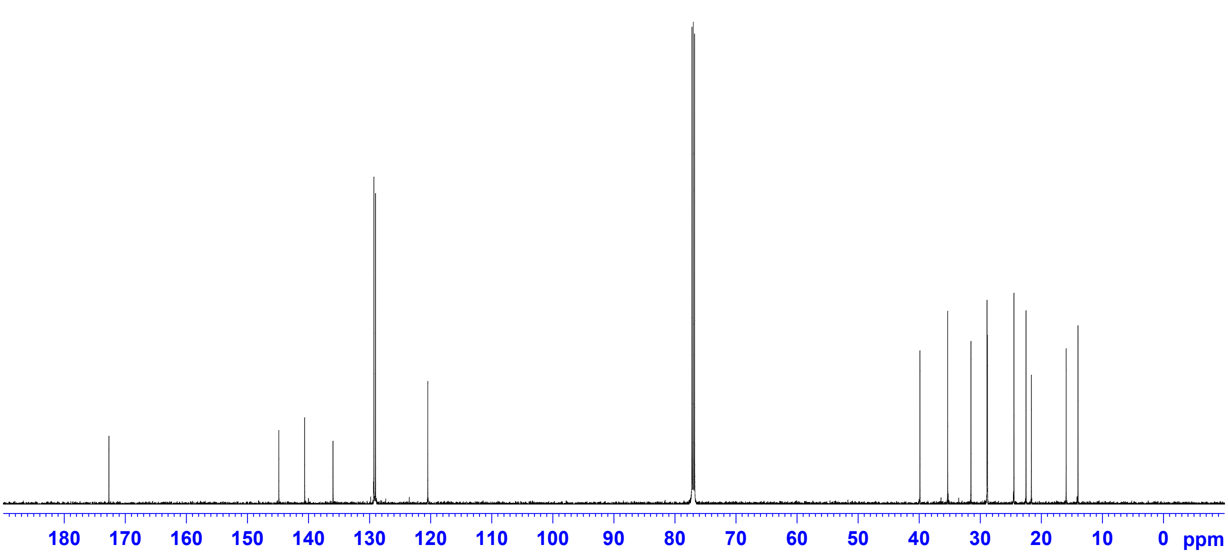

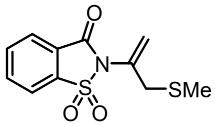

7
$CDCl_3$, 600 MHz

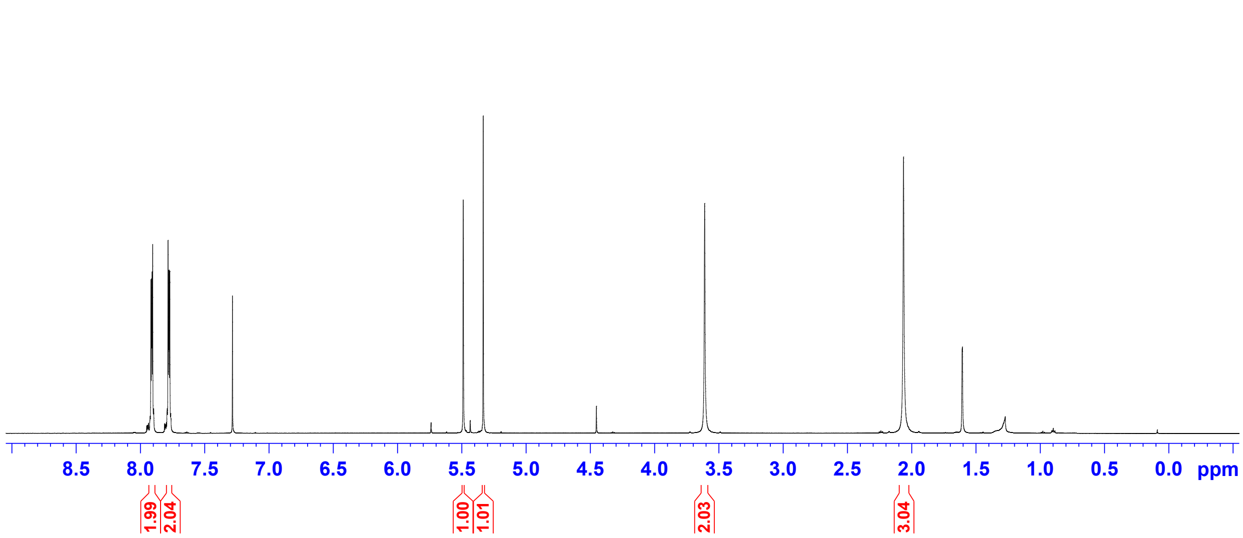

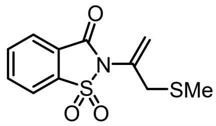

7
$CDCl_3$, 150 MHz

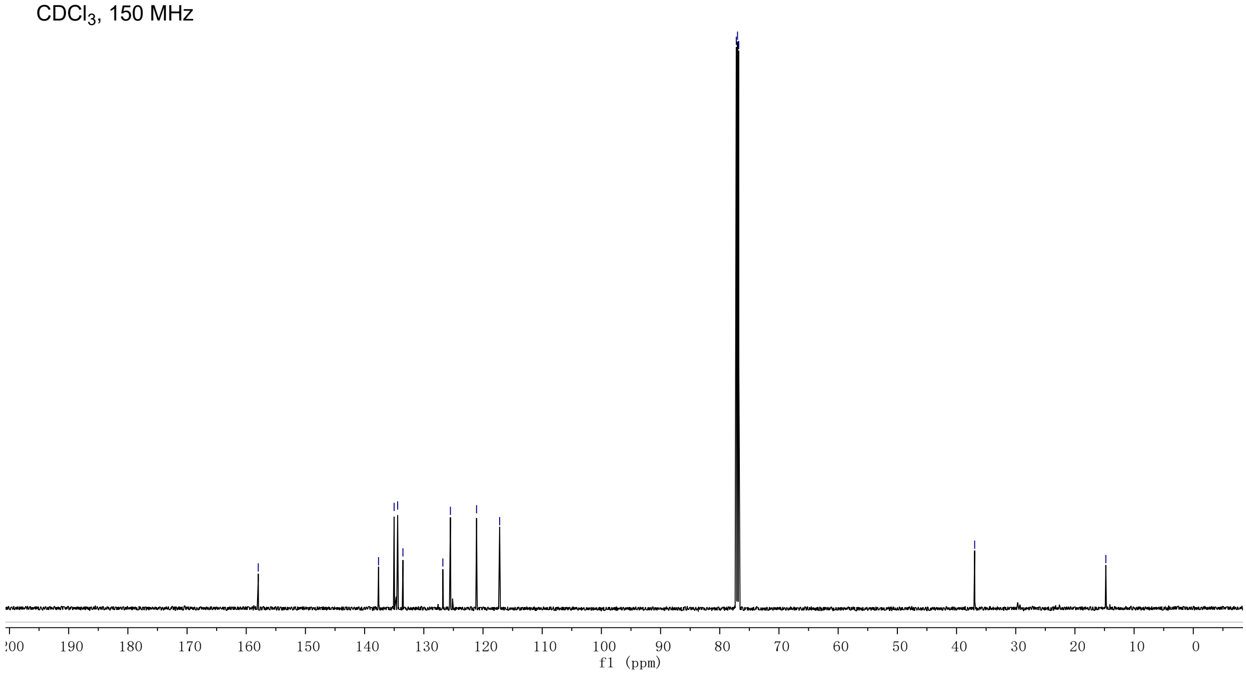

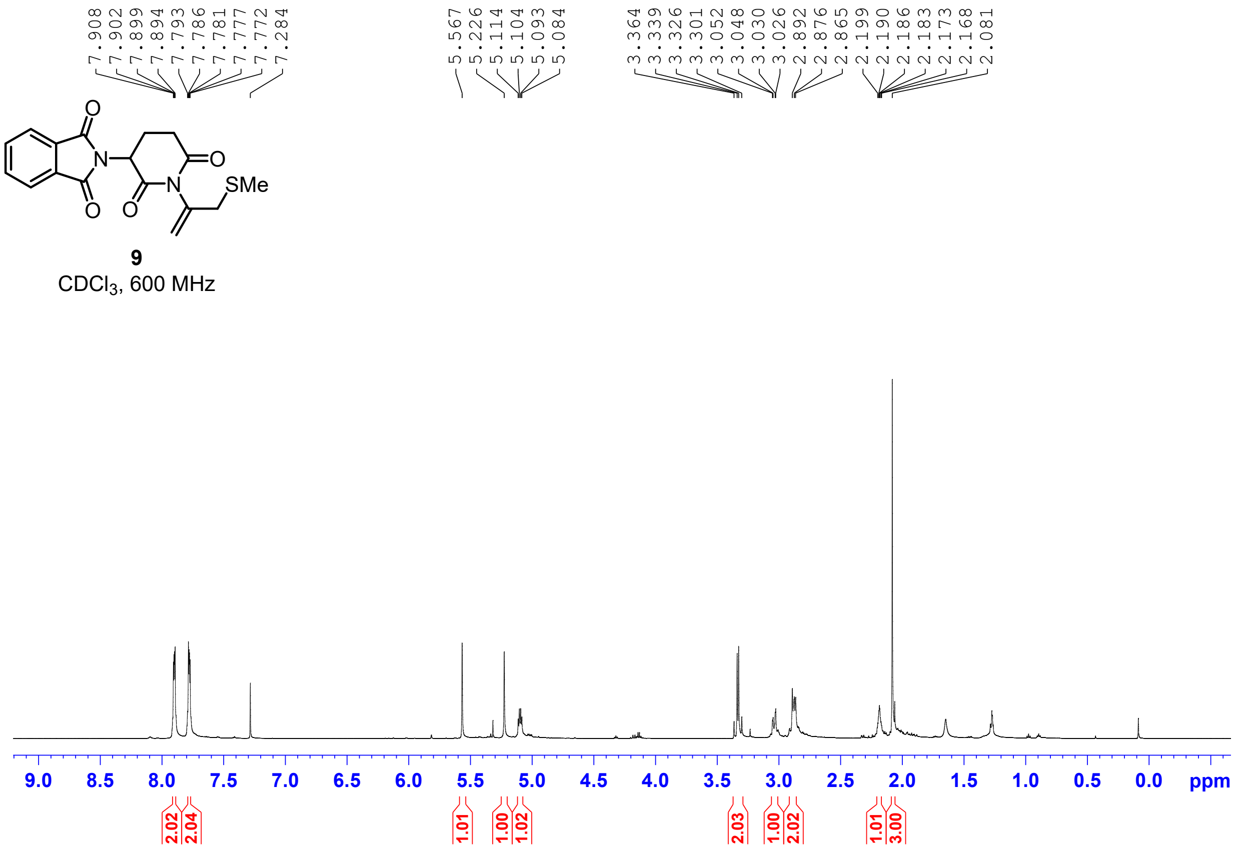
7.908
7.902
7.899
7.894
7.793
7.786
7.781
7.777
7.772
7.284
5.567
5.226
5.114
5.104
5.093
5.084
3.364
3.339
3.326
3.301
3.052
3.048
3.030
3.026
2.892
2.876
2.865
2.199
2.190
2.186
2.183
2.173
2.168
2.081
SMe
9
$CDCl_3$, 600 MHz
9.0 8.5 8.0 7.5 7.0 6.5 6.0 5.5 5.0 4.5 4.0 3.5 3.0 2.5 2.0 1.5 1.0 0.5 0.0 ppm
2.02 2.04 1.01 1.00 1.02 2.03 1.00 2.02 1.01 3.00

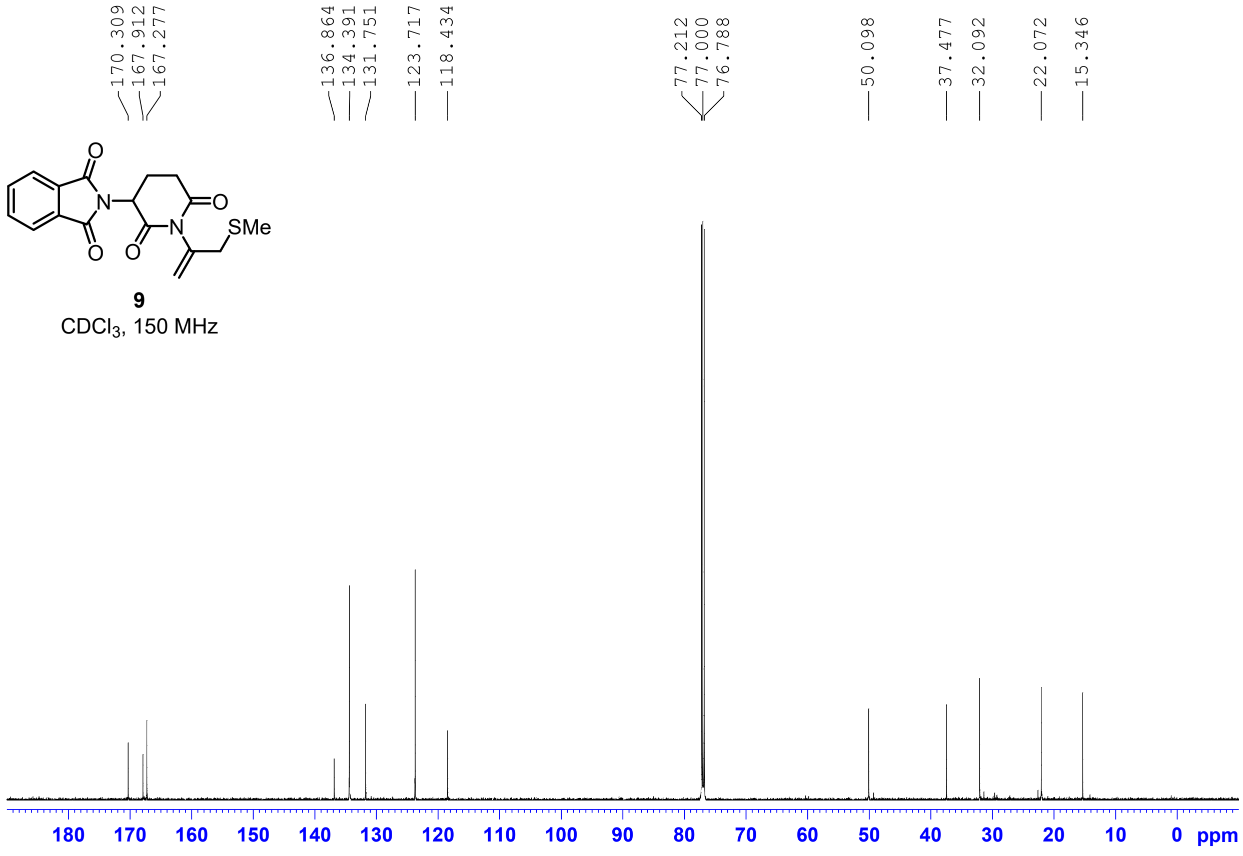
170.309
167.912
167.277
136.864
134.391
131.751
123.717
118.434
77.212
77.000
76.788
50.098
37.477
32.092
22.072
15.346
SMe
9
$CDCl_3$, 150 MHz
180 170 160 150 140 130 120 110 100 90 80 70 60 50 40 30 20 10 0 ppm

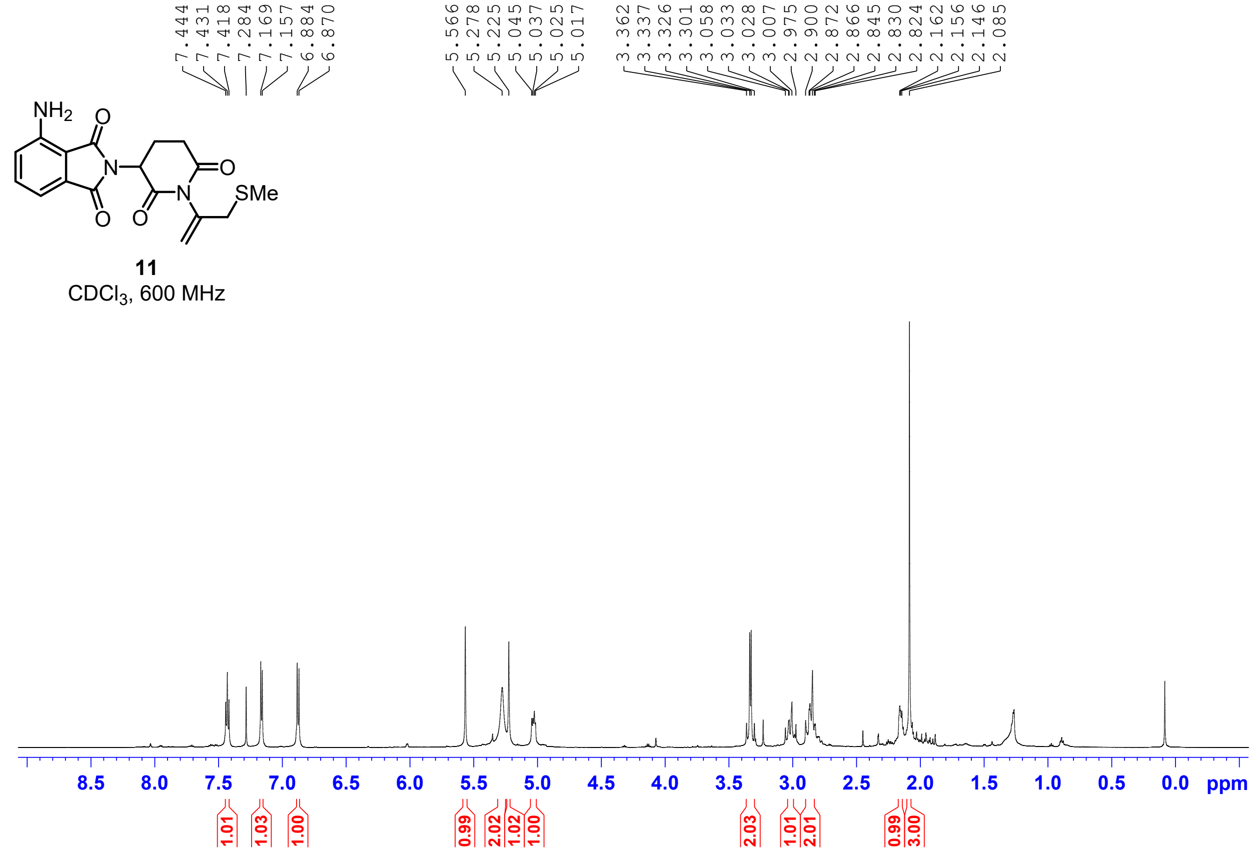

11
$CDCl_3$, 600 MHz

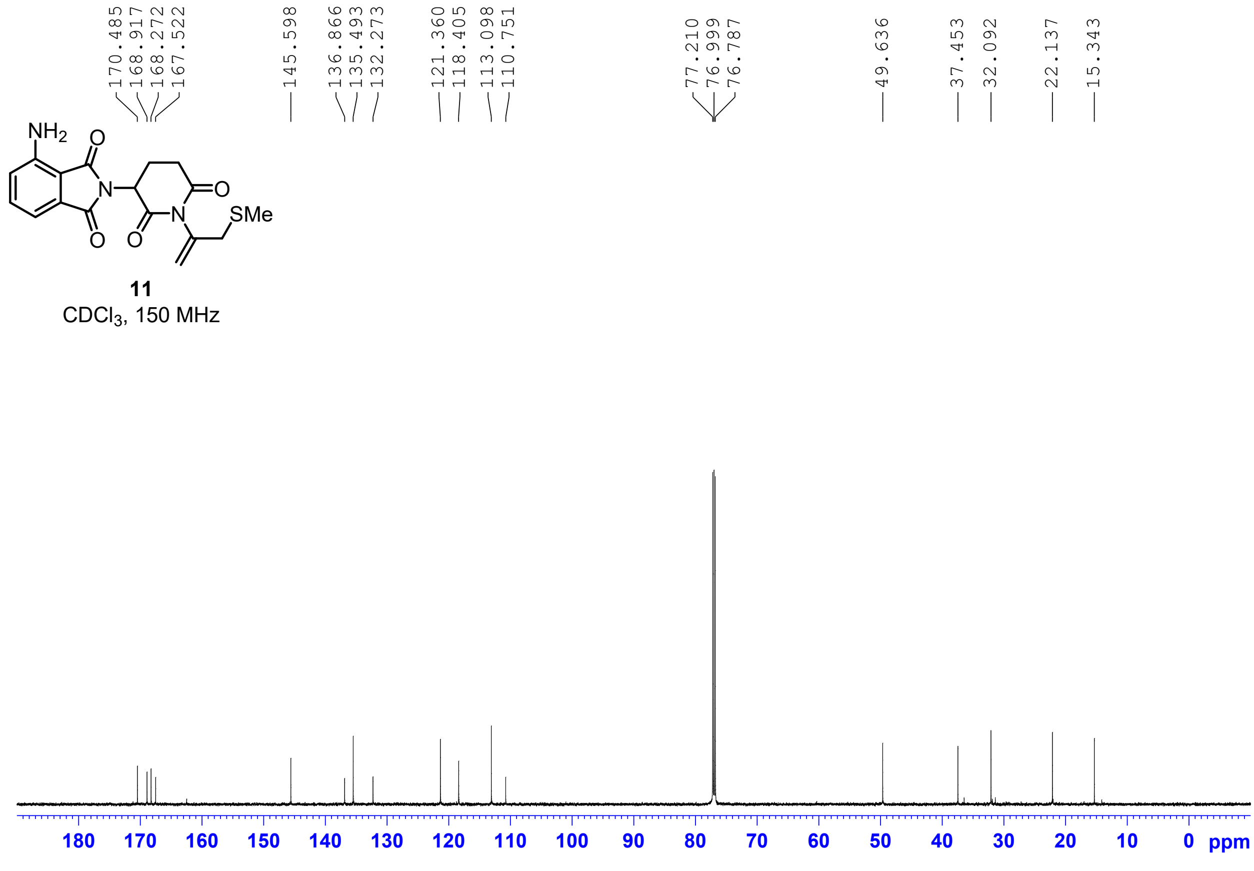

11
$CDCl_3$, 150 MHz

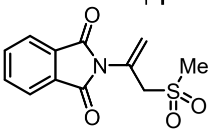

12

$CDCl_3$, 600 MHz

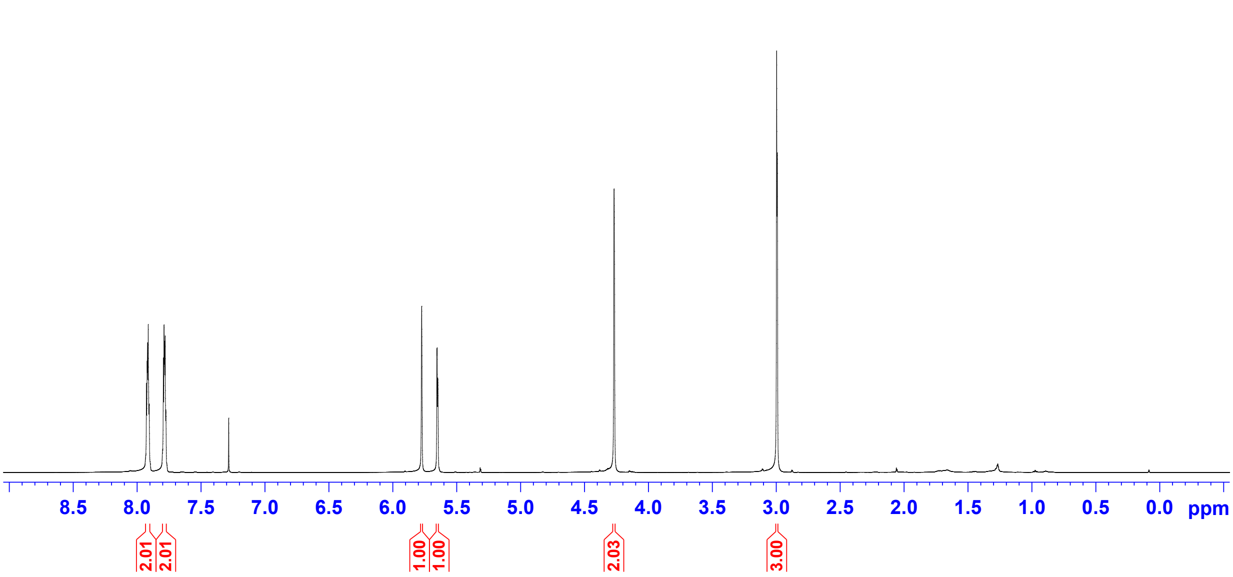

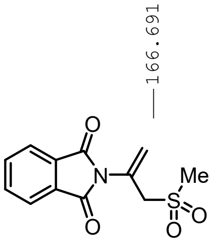

12

$CDCl_3$, 150 MHz

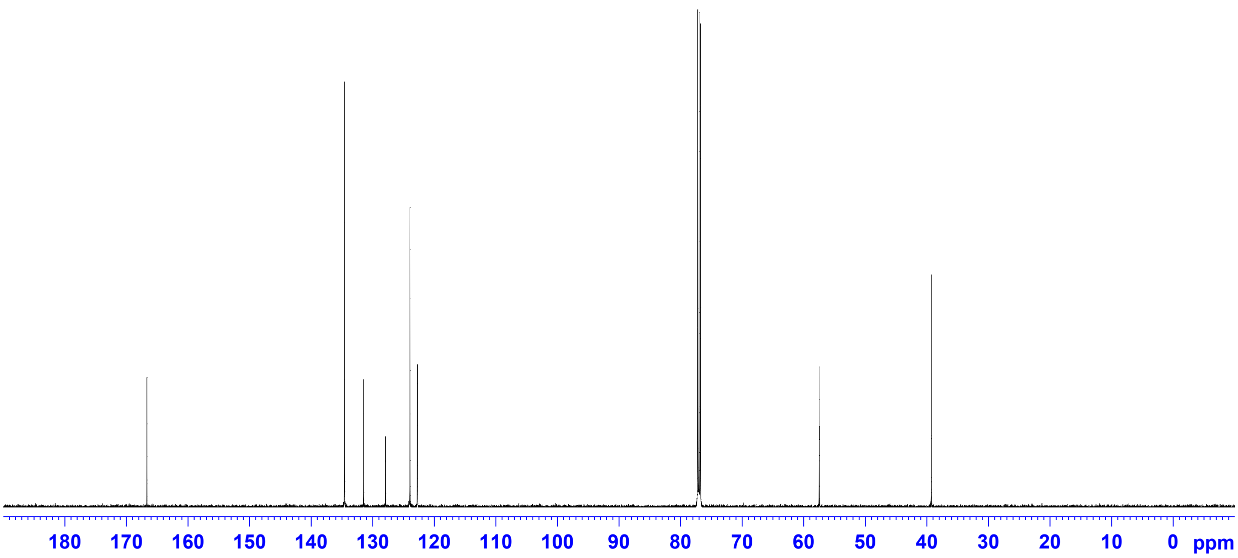

Supplement: RA-012-D2RA01117D-s001 [file RA-012-D2RA01117D-s001.pdf]
